# Comparative Evolution of Sand Fly Salivary Protein Families and Implications for Biomarkers of Vector Exposure and Salivary Vaccine Candidates

**DOI:** 10.3389/fcimb.2018.00290

**Published:** 2018-08-29

**Authors:** Iliano V. Coutinho-Abreu, Jesus G. Valenzuela

**Affiliations:** Vector Molecular Biology Section, Laboratory of Malaria and Vector Research, National Institute of Allergy and Infectious Diseases, National Institutes of Health, Rockville, MD, United States

**Keywords:** salivary proteins, molecular evolution, markers of exposure, vaccines, sand flies, Leishmania, Leishmaniasis

## Abstract

Sand fly salivary proteins that produce a specific antibody response in humans and animal reservoirs have been shown to be promising biomarkers of sand fly exposure. Furthermore, immunity to sand fly salivary proteins were shown to protect rodents and non-human primates against *Leishmania* infection. We are missing critical information regarding the divergence amongst sand fly salivary proteins from different sand fly vectors, a knowledge that will support the search of broad or specific salivary biomarkers of vector exposure and those for vaccines components against leishmaniasis. Here, we compare the molecular evolution of the salivary protein families in New World and Old World sand flies from 14 different sand fly vectors. We found that the protein families unique to OW sand flies are more conserved than those unique to NW sand flies regarding both sequence polymorphisms and copy number variation. In addition, the protein families unique to OW sand flies do not display as many conserved cysteine residues as the one unique to the NW group (28.5% in OW vs. 62.5% in NW). Moreover, the expression of specific protein families is restricted to the salivary glands of unique sand fly taxon. For instance, the ParSP15 family is unique to the Larroussius subgenus whereas phospholipase A2 is only expressed in member of Larroussius and Adlerius subgenera. The SP2.5-like family is only expressed in members of the Phlebotomus and Paraphlebotomus subgenera. The sequences shared between OW and NW sand flies have diverged at similar rates (38.7 and 45.3% amino acid divergence, respectively), yet differences in gene copy number were evident across protein families and sand fly species. Overall, this comparative analysis sheds light on the different modes of sand fly salivary protein family divergence. Also, it informs which protein families are unique and conserved within taxon for the choice of taxon-specific biomarkers of vector exposure, as well as those families more conserved across taxa to be used as pan-specific vaccines for leishmaniasis.

## Introduction

Vector borne diseases represent almost half of the neglected tropical infectious diseases. When attempting to get a blood meal, most vectors of disease deliver the pathogen in the host skin and together with the pathogen these arthropods deliver saliva (Coutinho-Abreu et al., 2015; de Castro et al., 2017). Blood sucking arthropods secrete a plethora of bioactive compounds in their saliva to counteract the mammalian host hemostatic system in order to get a successful blood meal. Salivary anti-hemostatic components such as anticoagulants (Chagas et al., 2014), vasodilators (Ribeiro et al., 1989; Lerner et al., 1991; Champagne and Ribeiro, 1994), and inhibitors of platelet aggregation (Calvo et al., 2007, 2011; Assumpcao et al., 2013) have been described and some of these proteins characterized at the molecular level (Coutinho-Abreu et al., 2015). In addition, the biological activity of some arthropod salivary proteins was shown to promote the establishment of pathogens in the mammalian host (Coutinho-Abreu et al., 2015; de Castro et al., 2017). Furthermore, immunity to specific sand fly salivary proteins was shown to protect rodents and non-human primates against leishmaniasis (Kamhawi et al., 2000; Gomes et al., 2008; Collin et al., 2009; Oliveira et al., 2015).

It is well established that humans and animal reservoirs make antibodies to proteins in the saliva of insects including those present in sand flies. These findings have prompted research groups to explore the use of sand fly salivary proteins as markers of sand fly exposure for humans and animal reservoirs (Teixeira et al., 2010; Drahota et al., 2014; Marzouki et al., 2015; Sima et al., 2016; Kostalova et al., 2017). A recombinant sand fly salivary protein of 43 kDa (rSP03B), from the sand fly *P. pernicious*, belonging to the yellow family of proteins was shown to be a marker of sand fly exposure in dogs living in Southern and Central Italy and in Portugal (Drahota et al., 2014; Kostalova et al., 2017). Interestingly, the yellow related protein from *P. orientalis* (rPorSP24) was demonstrated to be a good marker of sand fly exposure in domestic animals including dogs from a *L. donovani* foci in Ethiopia (Sima et al., 2016). For humans living in visceral leishmaniasis disease endemic areas in Brazil, the combination of salivary proteins LJM11 and LJM17 (yellow related proteins) was demonstrated to be the best biomarker of *Lutzomyia longipalpis* exposure in humans (Teixeira et al., 2010), while Linb13, a protein of 30 kDa belonging to the antigen-5 family of proteins, was shown to be the best biomarker of *Lutzomyia intermedia* exposure in humans living in a cutaneous leishmaniasis endemic area (Carvalho et al., 2017). The salivary protein PpSP32 from *P. papatasi* was demonstrated to be the marker of *P. papatasi* exposure for humans living in cutaneous leishmaniasis endemic areas in Tunisia (Marzouki et al., 2015) and in Saudi Arabia (Mondragon-Shem et al., 2015).

Based on their continental separation, sand flies are classified as belonging to New World (NW) and Old World (OW) groups. Thus far, over a dozen sand fly salivary gland transcriptomes have been obtained. From the OW group, 10 species have had their salivary transcriptomes decoded. These species belong to five subgenera of the genus Phlebotomus, including the subgenera Phlebotomus, Paraphlebotomus, Larroussius, Adlerius, and Euphlebotomus (Valenzuela et al., 2001; Anderson et al., 2006; Kato et al., 2006; Oliveira et al., 2006; Hostomská et al., 2009; Abdeladhim et al., 2012; Rohousova et al., 2012; Martín-Martín et al., 2013; Vlkova et al., 2014). For NW sand flies, salivary gland transcriptomes were sequenced from four species. Within the genus Lutzomyia, the salivary transcriptomes of *Lu. longipalpis* (subgenus Lutzomyia) and *Lutzomyia ayacuchensis* (subgenus *Helcocyrtomyia*), as well as two other transcriptomes from sand flies in the genera *Nyssomyia* (*Nyssomyia intermedia*) and *Bichromomyia* (*Bichromomyia olmeca*) have been obtained (Valenzuela et al., 2004; de Moura et al., 2013; Kato et al., 2013; Abdeladhim et al., 2016).

The use of sand fly salivary proteins as markers of vector exposure represents therefore a practical application that can be implemented in epidemiological studies as well as for vector control programs. Therefore, having a well-defined catalog of sandfly salivary proteins as well as a better understanding of the evolutionary relationship of salivary proteins from different sand fly vectors will allow us to make more precise selection for these appealing biomarkers. In the current study, we focused on the evolutionary analysis of protein families unique to Old World (OW) sand flies comparing that with the protein families shared between OW and New World (NW) sand flies as well as those unique to NW sand flies. This comparative analysis also unveiled that the some salivary protein families unique to OW sand flies emerged and diversified in different manners than their counterparts unique to NW sand flies. These findings can inform which proteins are the best candidates to be used as a pan-specific or species-specific biomarkers of sand fly exposure or as well as a pan-specific vaccine against leishmaniasis.

## Materials and methods

### Sequences

Nucleotide and amino acid sequences were retrieved from the NCBI databases from sand fly salivary gland transcriptomes (Valenzuela et al., 2001, 2004; Anderson et al., 2006; Oliveira et al., 2006; Hostomská et al., 2009; Abdeladhim et al., 2012, 2016; Rohousova et al., 2012; de Moura et al., 2013; Kato et al., 2013; Martín-Martín et al., 2013; Vlkova et al., 2014). Signal peptides were removed from the protein sequences whereas sequences encoding signal peptides and stop codons were removed from the nucleotide sequences for further analyses. Only sequences displaying more than 5% divergence at the amino acid level were assumed to be encoded by true paralog genes and included in the analyses, rather than being alleles of the same gene and otherwise discarded. Sand fly groups, species, and sequence accession numbers are provided in Supplementary Table 1.

### Sequence alignment

Multiple sequence alignments of putative peptides were carried out using Clustal Omega built in the MacVector software 15.8 (Olson, 1994) and in MEGA7 (Kumar et al., 2016). For the construction of phylogenetic trees, the gap penalties were not taken into account in the multiple sequence alignments.

### DNA polymorphism, protein divergence, and phylogenetic analysis

The evolutionary analyses were performed in the DnaSP 5.10 software (Librado and Rozas, 2009). The parameter ω refers to the rate of non-synonymous nucleotide polymorphisms (Ka) over the synonymous rate of nucleotide polymorphisms (Ks) (Nei, 1987). Slide window analyses of ω along the nucleotide sequences encoding such proteins were also obtained. The diversity of the protein family sequences refers to the p-distance [proportion (*p*) of amino acid sites at which the two sequences to be compared are different (Nei, 2000)] obtained in the MEGA7 software (Kumar et al., 2016).

The evolutionary histories of salivary protein families were inferred by using the Maximum Likelihood method and conducted in MEGA7 (Kumar et al., 2016). The amino acid substitution model was selected based on the best fit provided by the Model Selection tool built in the MEGA 7 software. The bootstrap consensus trees inferred from 1,000 replicates (Felsenstein, 1985) were taken to represent the evolutionary history of the taxa analyzed (Felsenstein, 1985). Branches corresponding to partitions reproduced in <50% bootstrap replicates are collapsed. Initial tree(s) for the heuristic search were obtained by applying the Neighbor-Joining method to a matrix of pairwise distances estimated using a JTT model (Felsenstein, 1985).

### Statistical analysis

D'Agostino and Pearson normality test was performed to assess whether or not data follow a normal distribution, and the Mann–Whitney test was carried out in order to test the significance of the differences in protein divergence and omega (ω) values. Both tests were performed using the Prism 7 software (GraphPad).

## Results

### Updated sand fly salivary protein catalog

**Figure 1:**
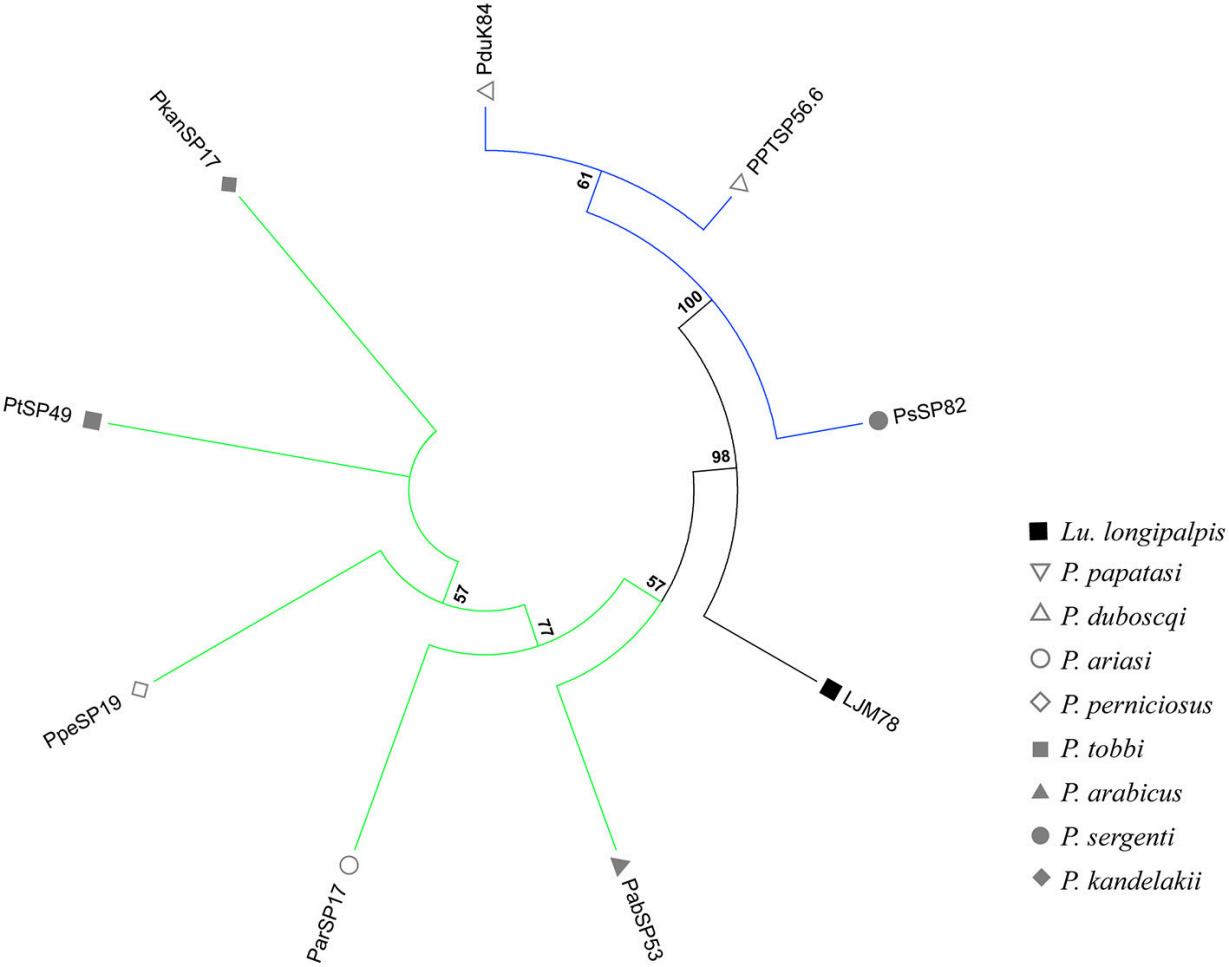
Phylogenetic analysis of ParSP17 protein family. The evolutionary history was inferred by using the Maximum Likelihood method based on the Le Gascuel matrix-based model (Gomes et al., 2008). Sand fly species are indicated by the different symbols in the legend on the right. Tree branches were color-coded so as to represent specific taxon: Green color represents the Larroussius and Adlerius subgenera; Blue color points to proteins of the Phlebotomus and Paraphlebotomus subgenera; and Black color indicates the proteins belonging to New World sand flies. Although the PduK84 sequence is illustrated here, it was not included in further analyses because it is truncated.

From 14 sand fly salivary gland transcriptomes we compiled the sand fly salivary proteins unique to either NW or OW sand flies as well as protein families common to all sand fly species and developed an updated catalog for all the analyzed sand fly salivary proteins (Table 1). With this extensive catalog of sand fly salivary proteins we identified 12 protein families shared between OW and NW sand flies, lufaxin, apyrase, yellow-related protein, silk-related protein or PpSP32, endonuclease, hyaluronidase, adenosine deaminase, small odorant binding-like proteins, D7 family of proteins, antigen-5 related protein, ParSP17 (39 kDa protein of unkown function), ParSP80 (16 kDa protein of unkown function) (Figure 1 and Supplementary Figures 4–15), seven protein families unique to OW sand flies, pyrophosphatase, phospholipase A2, ParSp15 (5 kDa protein of unknown function), ParSP25 (32 kDa protein of unknown function), SP16 (14.3 kDa protein of unknown function), ParSP23 (2 kDa protein of unknown function), and SP2.5 (2.5 kDa protein of unknown function, **Figures 7**–**10** and Supplementary Figures 1–3). Two protein families unique to OW sand flies that are only shared by two sand fly species (ParSP23-like and ParSP2.5-like; Supplementary Figures 2,3). We also identified 12 protein families unique to NW sand flies: toxin, RGD, c-type lectin, 14 kDa protein family, ml domain, 5′ nucleotidase, 9 kDa protein family, salo, 11.5 kDa protein family, maxadilan, 71 kDa protein family, and 5 kDa protein family.

**Table 1:** Sand fly salivary protein families across multiple sand fly species.

**Family of proteins**	***B. olmeca***	***L. longipalpis***	***Lu. ayacuyensis***	***N. intermedia***	***P. papatasi***	***P. duboscqi***	***P. sergenti***	***P. ariasi***	***P. orientalis***	***P. tobbi***	***P. perniciosus***	***P. kandelakki***	***P. arabicus***	***P. argentipes***
**PROTEINS UNIQUE TO NEW WORLD SAND FLIES**														
Toxin	X	–	–	X	–	–	–	–	–	–	–	–	–	–
RGD-containing	X	X	X	X	–	–	–	–	–	–	–	–	–	–
C-type lectin	X	X	X	X	–	–	–	–	–	–	–	–	–	–
Maxadilan	–	X	–	X	–	–	–	–	–	–	–	–	–	–
14kDa SP	X	X	–	X	–	–	–	–	–	–	–	–	–	–
ML domain	X	–	–	X	–	–	–	–	–	–	–	–	–	–
5′ Nucleotidase	–	X	–	–	–	–	–	–	–	–	–	–	–	–
9 kDa SP	–	X	X	–	–	–	–	–	–	–	–	–	–	–
SALO	X	X	–	X	–	–	–	–	–	–	–	–	–	–
11.5 kDa SP	X	X	X	–	–	–	–	–	–	–	–	–	–	–
71 kDa proteins	X	X	X	–	–	–	–	–	–	–	–	–	–	–
5 kDa protein	X	–	–	X	–	–	–	–	–	–	–	–	–	–
**PROTEINS UNIQUE TO OLD WORLD SAND FLIES**														
Pyrophosphatase	–	–	–	–	–	X	–	–	X	–	X	X	X	X
Phospholipase A2	–	–	–	–	–	–	–	X	X	–	X	X	X	–
SP16	–	–	–	–	X	–	X	–	X	–	–	–	X	X
ParSP23	–	–	–	–	-	–	–	X	–	–	–	–	X	–
SP2.5	–	–	–	–	X	X	–	–	–	–	–	–	–	–
ParSP25	–	–	–	–	–	–	–	X	X	X	X	X	X	X
ParSP15	–	–	–	–	–	–	–	X	X	X	X	X	–	–
**PROTEINS SHARED BETWEEN NEW WORLD AND OLD WORLD SAND FLIES**														
OBPs	X	X	X	X	X	X	X	X	X	X	X	X	X	X
Yellow-related	X	X	X	X	X	X	X	X	X	X	X	X	X	X
Antigen 5	X	X	X	X	X	X	X	X	X	X	X	X	X	X
Lufaxin	X	X	X	X	X	X	X	X	X	X	X	X	X	X
D7-related	X	X	X	X	X	X	X	X	X	X	X	X	X	X
Apyrase	X	X	X	X	X	X	X	X	X	X	X	X	X	X
Silk related	X	X	X	X	X	X	X	X	X	X	X	X	X	X
Hyaluronidase	X	X	–	X	–	–	–	–	X	X	X	X	X	–
Endonuclease	X	X	X	X	–	–	–	X	X	–	X	X	X	X
Adenosine deaminase	X	X	–	–	–	X	–	–	–	–	X	–	–	–
ParSP17	–	X	–	–	X	X	X	X	–	X	X	X	X	–
ParSP80	–	X	–	–	–	X	–	X	–	–	–		X	–

*X, protein expressed in the saliva*.

–*, not expressed or not detected*.

### Salivary proteins shared between old world and new world sand flies

The phylogenetic trees of 9 out of 12 salivary protein-encoding gene families shared between OW and NW sand flies (Supplementary Figures 4–15) correlated well with the sand fly species phylogeny, constructed based on ITS-2 sequences (Aransay et al., 2000). The resulting phylogenetic tree indicates that these shared gene families of sand fly salivary proteins have been evolving under purifying (negative) selection or are selective neutral (ω < 1; Barton and Etheridge, 2004). Such salivary protein families encompassed lufaxin, apyrase, yellow-related protein, antigen-5, endonuclease, hyaluronidase, ParSP80, odorant binding proteins (OBPs), and adenosine deaminase. On the other hand, Silk-related protein or PpSP32, the D7 family of proteins (Supplementary Figures 6,12; Abdeladhim et al., 2016) and the ParSP17-like family of proteins (Figure 1) displayed phylogenies diverging from the sand fly species phylogeny, pointing that strong selective forces related to the function and/or immunogenicity of such proteins have driven their evolution since the split of NW and OW sand flies that likely took place about 100MYs with the continental separation of the American continent from the European and African ones.

### Molecular evolution between OW and NW sand fly salivary proteins

**Figure 2:**
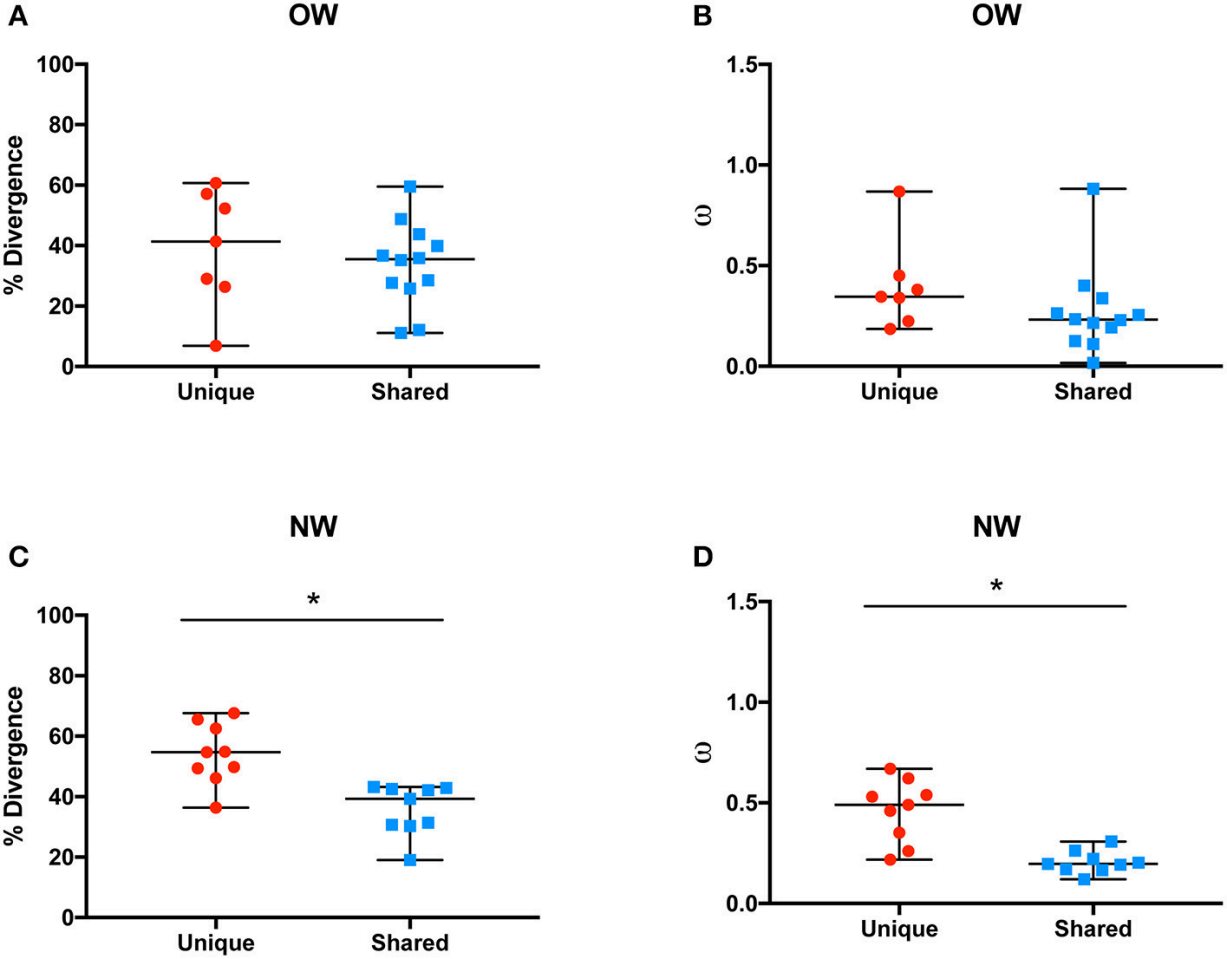
Average molecular divergence of unique and shared Old World **(A)** and New World **(C)** salivary proteins (top), and rate of non-synonymous over synonymous replacements (omega – ω) of unique and shared Old World **(B)** and New World **(D)** salivary proteins. ^*^p < 0.05.

The overall rates of molecular evolution of the salivary protein families unique to OW sand flies were similar to the families shared with NW sand flies (Figures 2A,B). The median protein divergences (% divergence) for the families unique to OW sand flies and shared with the NW ones were 41.4 and 35.5%, respectively (Figure 2A). Likewise, the median ratios of non-synonymous over synonymous replacements (ω) for the families unique to OW sand flies and shared with the NW ones were 0.34 and 0.23, respectively (Figure 2B). This is in sharp contrast to the pattern of divergence observed in the protein families unique to NW sand flies when compared with their counterparts shared with Old World sand flies (Figures 2C,D), as observed elsewhere (Abdeladhim et al., 2016). In the protein families unique to NW sand flies, the median protein divergence was 54.7% whereas 39.3% divergence was noticed for the protein families shared with OW sand flies (Figure 2C, *p* < 0.0008). By the same token, the median ω value was significantly greater in the protein families unique to NW sand flies (median ω = 0.49) as compared to those shared with OW sand flies (median ω = 0.2, *p* < 0.0008; Figure 2D).

### Molecular evolution within OW and NW sand flies

**Figure 3:**
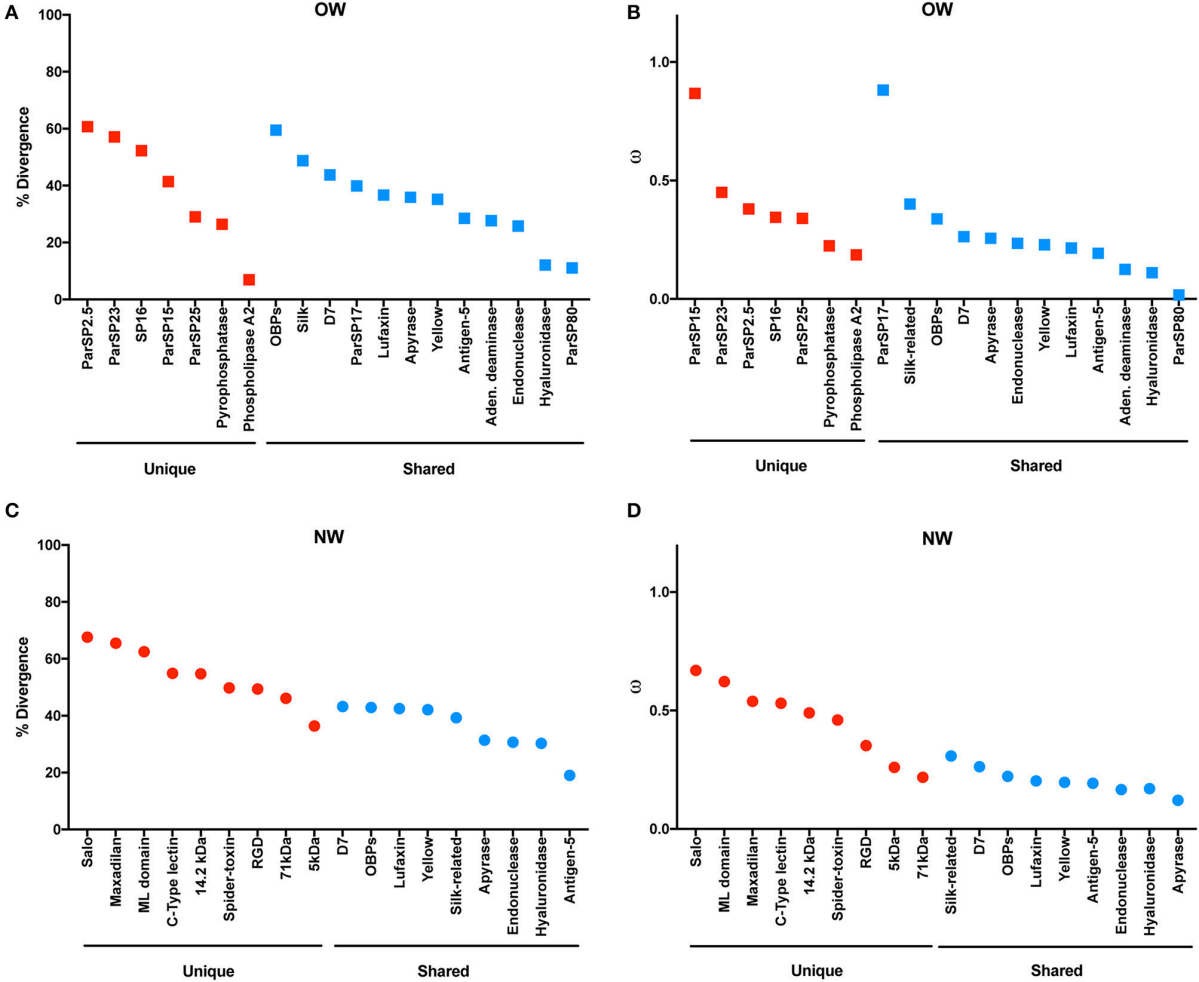
Molecular divergence across salivary protein in Old World **(A)** and New World **(C)** sand flies. Rate of non-synonymous over synonymous replacements (omega – ω) across salivary proteins in Old World **(B)** and New World **(D)** sand flies.

The molecular evolution of the protein families was also taken into account individually, in regard to the analyses of % divergence and the ratio of non-synonymous over synonymous replacements (ω; Figure 3). Among the protein families unique to OW sand flies, phospholipase A2, pyrophosphatase, and ParSP25-like displayed the lowest rates of diversification (divergence < 30%; ω < 0.35; Figures 3A,B) whereas ParSP2.5, ParSP23, and SP16 were among the most divergent families (divergence > 50%; ω > 0.44; Figures 3A,B). Within the OW sand fly protein families shared with NW sand flies, ParSP80, and hyaluronidase families showed consistently the lowest rates of sequence divergence and non-synonymous to synonymous codon replacement (divergence < 12%; ω < 0.12; Figures 3A,B). In contrast, the OBPs, silk-related, D7, and ParSP17 families exhibited the greatest rates of diversification (divergence > 39%; ω > 0.26; Figures 3A,B). Regarding the protein families unique to NW sand flies, the SALO, maxadilan, and ML domain families presented the highest rates of molecular evolution (divergence > 60%; ω > 0.62; Figures 3C,D; Abdeladhim et al., 2016) whereas the 5 kDa protein family presented itself as the least divergent protein family (divergence = 36%; ω = 0.26; Figures 3C,D; not previously assessed). Amongst the protein families of NW sand flies shared with OW species, the D7 family presented the highest levels of diversification (divergence = 43%; Figure 3C) whereas the antigen-5 protein family exhibited the lowest divergence at the amino acid level (divergence = 19%; Figure 3C). Regarding the ω values, the silk family displayed the highest ratio of non-synonymous over synonymous replacements (ω = 0.30; Figure 3D) whereas the lowest ratio was exhibited by the apyrase family. Regarding the OBP family, it is actually a superfamily, encompassing at least six protein families (Supplementary Figure 15). Hence, the high rates of divergence for the OBPs should be view with caution, as noted below.

### Molecular diversification along protein lengths

**Figure 4:**
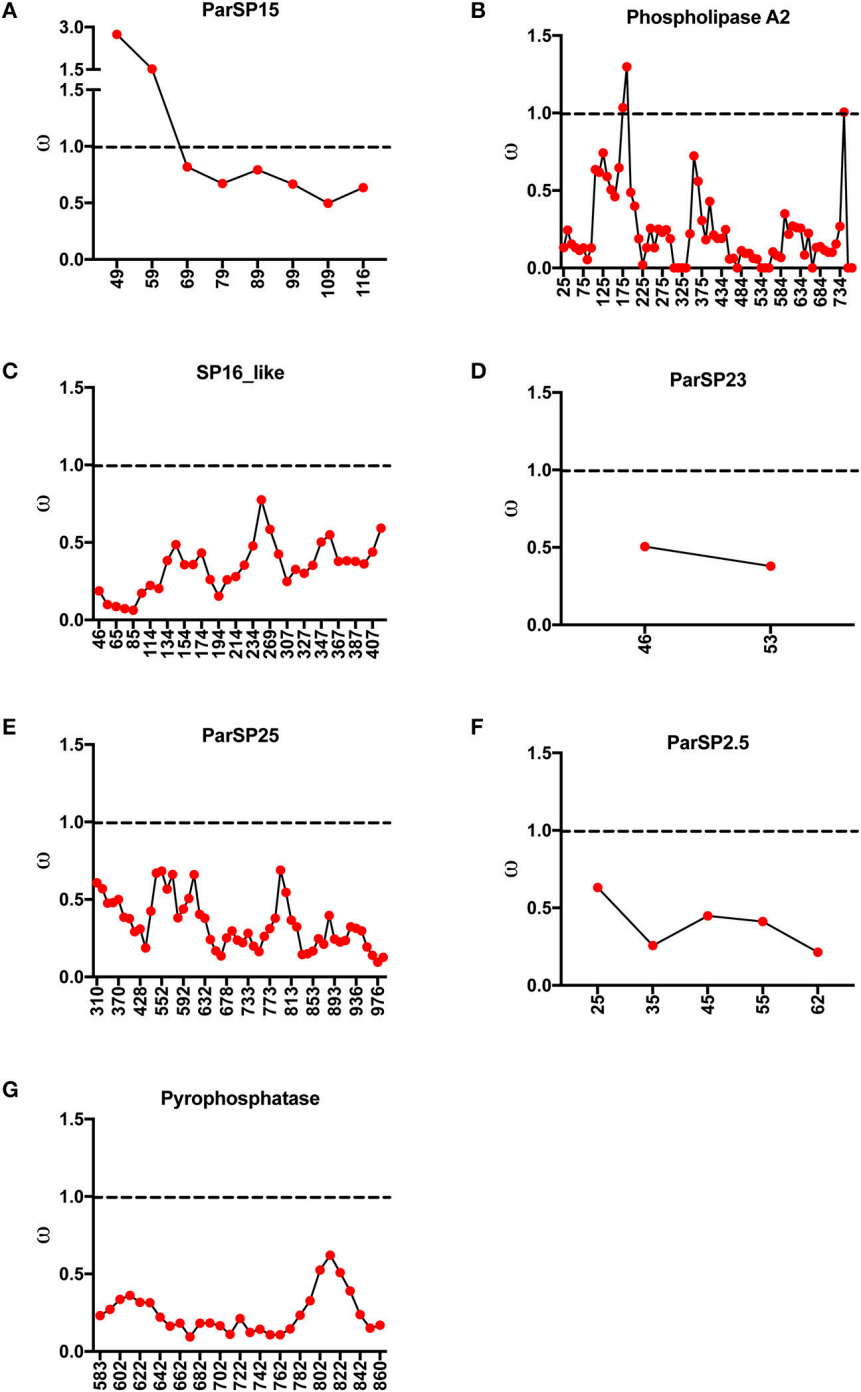
Slide window analyses of omega (ω) for the salivary protein families unique to Old World sand flies. Dotted lines point to the threshold for positive selection (ω ≥ 1). **(A)** ParSP15. **(B)** Phospholipase A2. **(C)** SP16. **(D)** ParSP23. **(E)** ParSP25. **(F)** ParSP2.5. **(G)** Pyrophosphatase.

**Figure 5:**
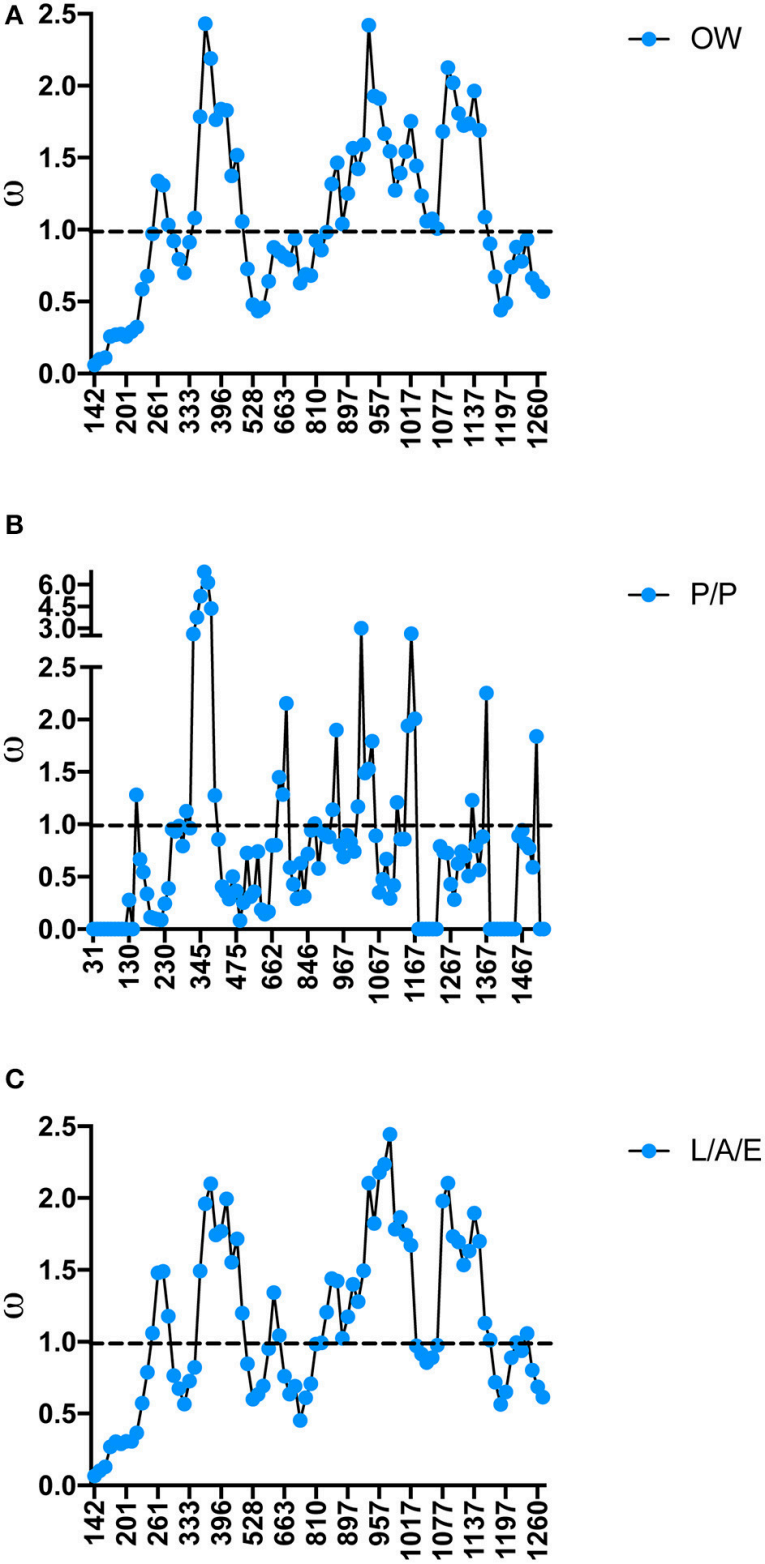
Slide window analyses of omega (ω) for the salivary protein ParSP17 in all the sand fly species **(A)**, Old World sand flies **(B)**, Phlebotomus and Paraphlebotomus sub-genera **(C)**, and Larrossious, Adlerius, and Euphlebotomus sub-genera. Dotted lines point to the threshold for positive selection (ω ≥ 1).

As specific regions of a protein can be subjected to different selective constraints and in turn evolve at different rates, slide-window analyses of non-synonymous over synonymous replacements (ω) for the genes encoding the salivary protein unique to OW sand flies and shared with NW sand flies were carried out (Figures 4, 5). Contrasting to the genes encoding the protein families unique to NW sand flies, which display multiple codons under positive (diversifying) or relaxed purifying selection (ω ≥ 1; Abdeladhim et al., 2016), the gene families unique to Old World sand flies exhibited for the most part codons under purifying (negative) selection (ω < 1; Figure 4). In the latter group, only ParSP15 and phospholipase A2 bore a few codons under positive selection (ω ≥ 1; Figure 4), mostly in the 5′ portion of the genes. The gene families shared with NW sand flies also displayed low levels of sequence divergence (Figure 3), with exception of the ParSP17 family which bear multiple codons under positive selection (Figure 5). In such (ParSP17; Figure 5A), a greater number of codons under positive selection were noticed in the gene sequences for the sand flies belonging to the related Phlebotomus and Paraphlebotomus subgenera (P/P; Figure 5B) than for the sand flies belonging to related Larrossious, Adhelerius, and Euphlebotomus subgenera (L/A/E; Figure 5C).

### Copy number variation of salivary protein-encoding genes across species

**Figure 6:**
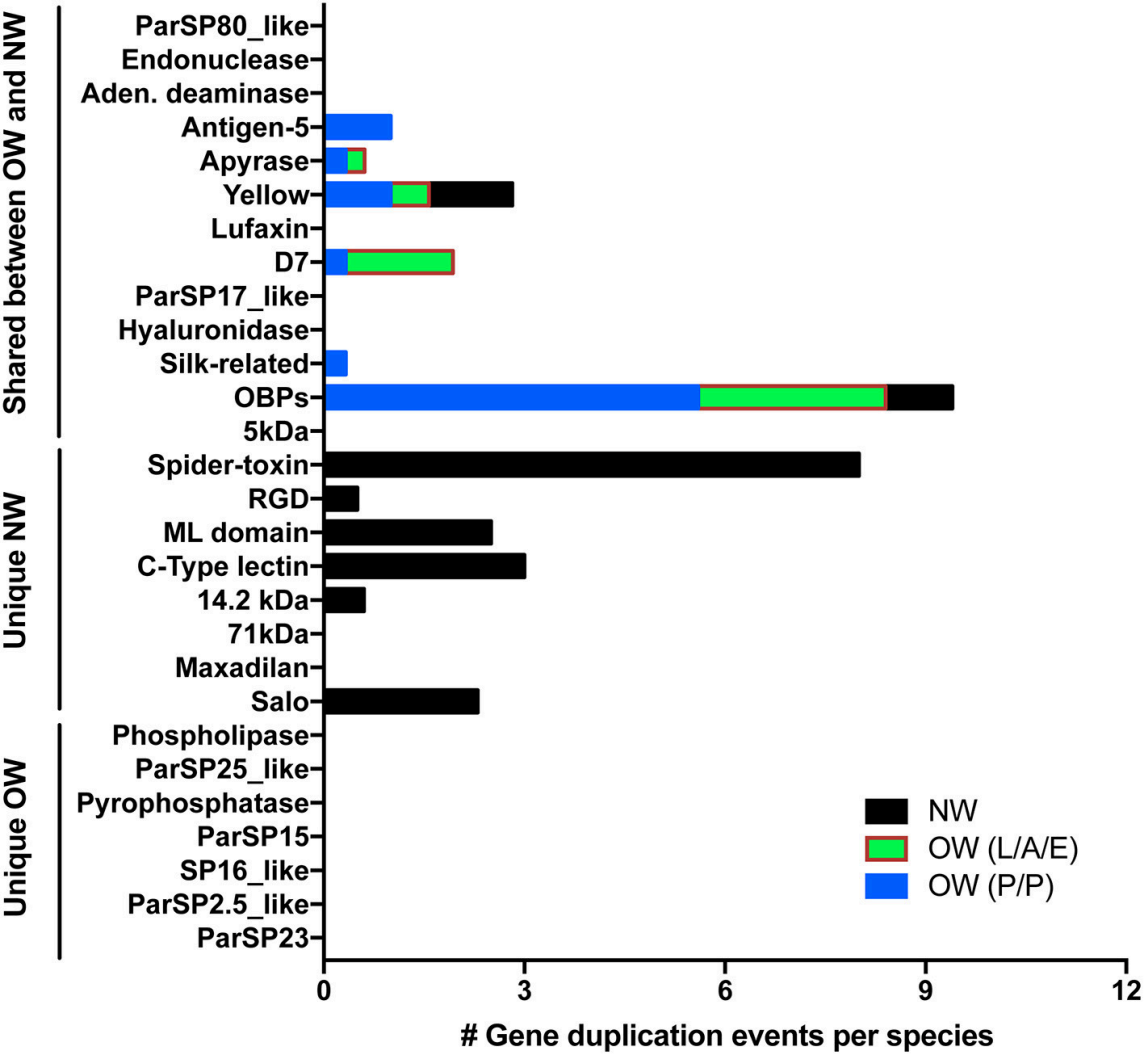
Copy number variation across sand fly salivary protein families. The number of gene duplication events were enumerated from the sequences within New World (NW—black boxes) as well as Old World (OW) sand flies (L/A/E—Larrossious, Adlerius, and Euphlebotomus subgenera—green and red boxes; P/P—Phlebotomus and Paraphlebotomus subgenera—blue boxes). Gene duplication events were normalized by the number of species represented in each group.

**Figure 7:**
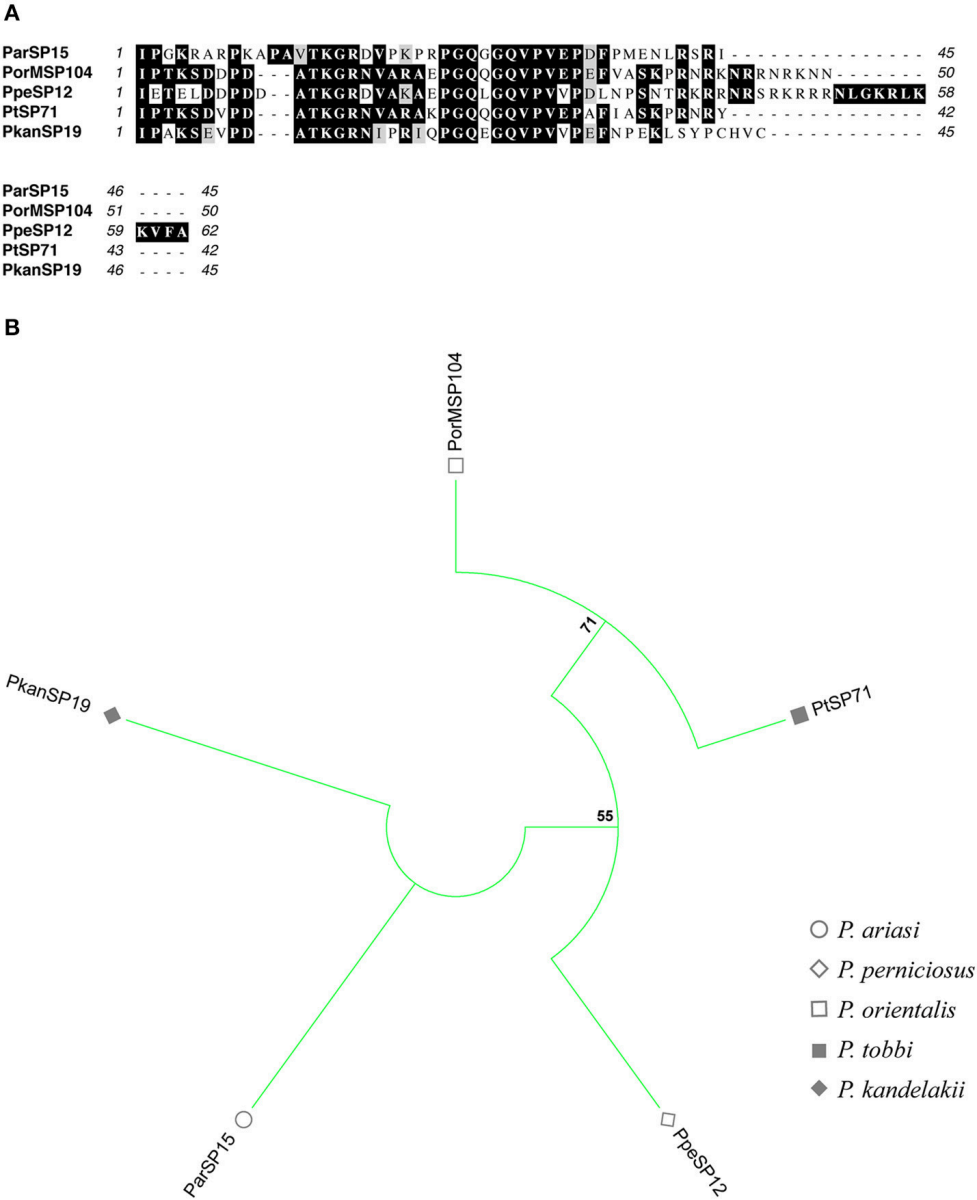
Multiple sequence alignment and molecular phylogenetic analysis of the sand fly **ParSP15** salivary protein family. **(A)** Multiple sequence alignment of **ParSP15 proteins**. ParSP15 (*P. ariasi*), PorMSP104 (*P. orientalis*), PpeSP12 (*P. perniciosus*), PtSP71 (*P. tobbi*), and PkanSP19 (*P. kandelakii*). Black background shading represents identical amino acids. Gray background shading represents similar amino acids. **(B)** The evolutionary history of **ParSP15** salivary protein family was inferred by using the Maximum Likelihood method based on the JTT matrix-based model (Jones et al., 1992). Sand fly species are indicated by the different symbols in the legend on the right.

**Figure 8:**
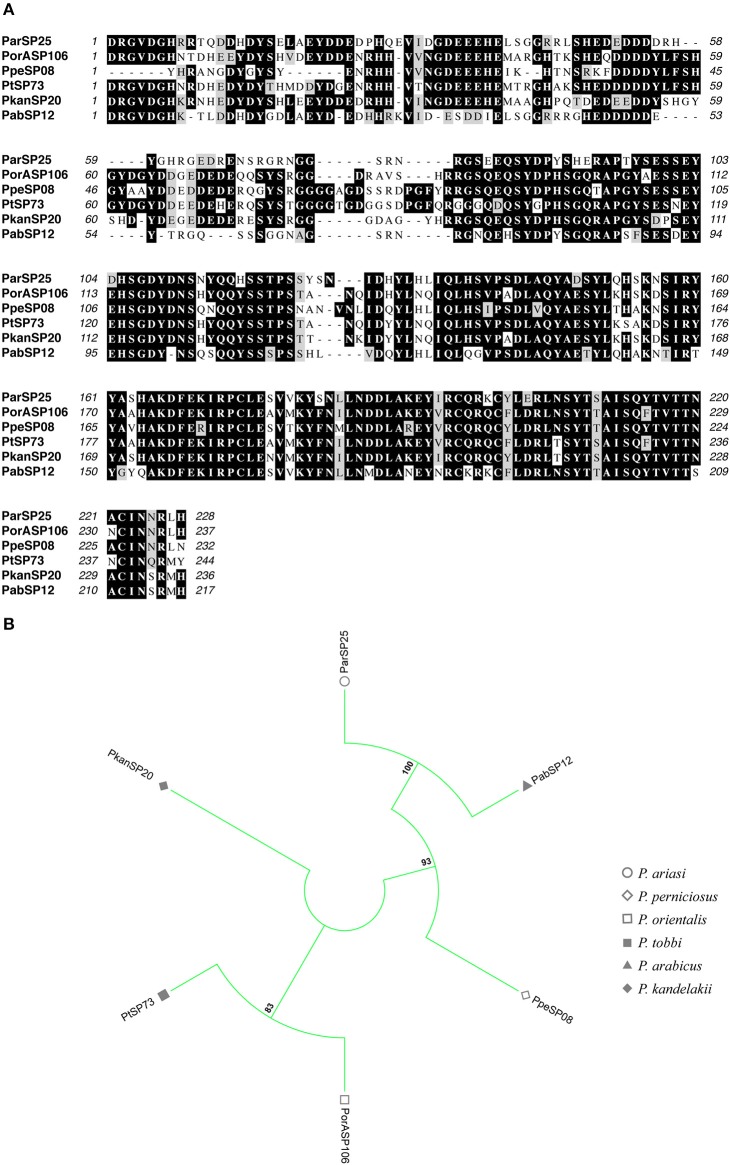
Multiple sequence alignment and molecular phylogenetic analysis of the sand fly **ParSP25** salivary protein family. **(A)** Multiple sequence alignment of **ParSP25 proteins**. ParSP25 (*P. ariasi*), PorASP106 (*P. orientalis*), PpeSP08 (*P. perniciosus*), PtSP73 (*P. tobbi*), PkanSP20 (*P. kandelakki*), and PabSP12 (*P. arabicus*). Black background shading represents identical amino acids. Gray background shading represents similar amino acids. **(B)** The evolutionary history of **ParSP25** salivary protein family was inferred by using the Maximum Likelihood method based on the JTT matrix-based model (Jones et al., 1992). Sand fly species are indicated by the different symbols in the legend on the right.

**Figure 9:**
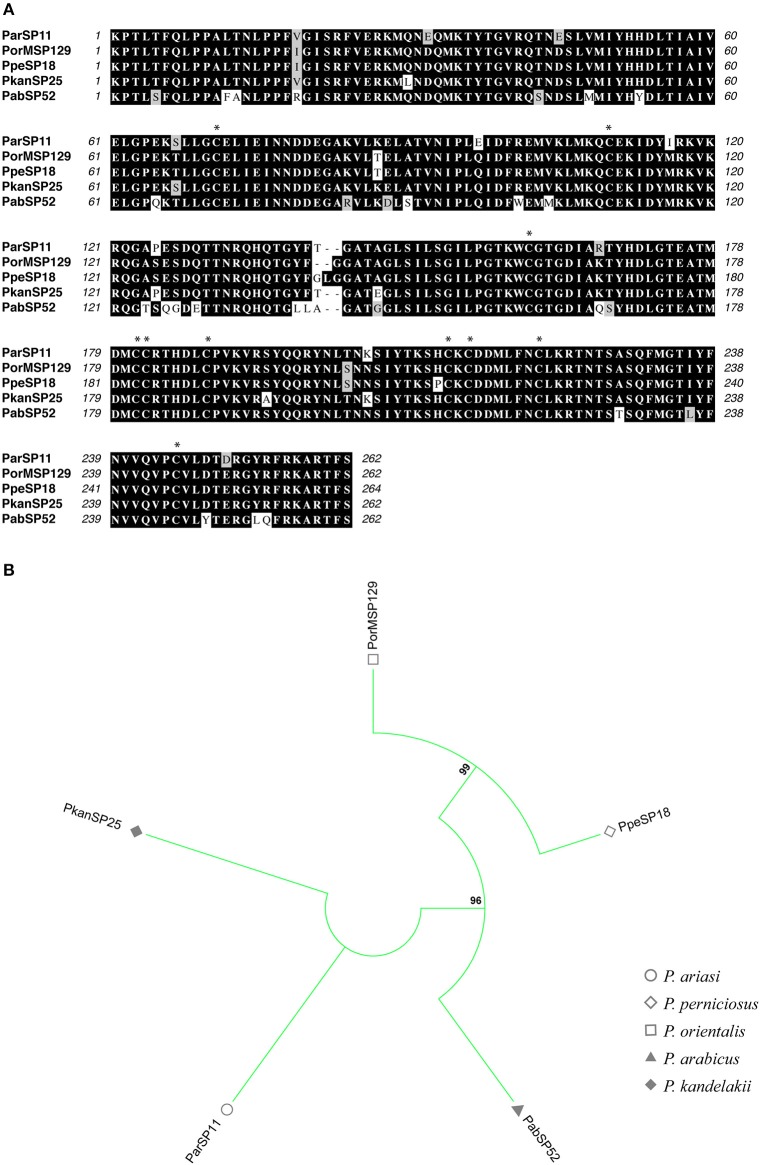
Multiple sequence alignment and molecular phylogenetic analysis of the sand fly **Phospholipase A2** salivary protein family. **(A)** Multiple sequence alignment of **Phospholipase A2 proteins**. ParSP11 (*P. ariasi*), PorMSP129 (*P. orientalis*), PpeSP18 (*P. perniciosus*), PkanSP25 (*P. kandelakki*), and PabSP52 (*P. arabicus*). Black background shading represents identical amino acids. Gray background shading represents similar amino acids. Asterisks indicate the conserved cysteine residues. **(B)** The evolutionary history of **Phospholipase A2** salivary protein family was inferred by using the Maximum Likelihood method based on the JTT matrix-based model (Jones et al., 1992). Sand fly species are indicated by the different symbols in the legend on the right.

**Figure 10:**
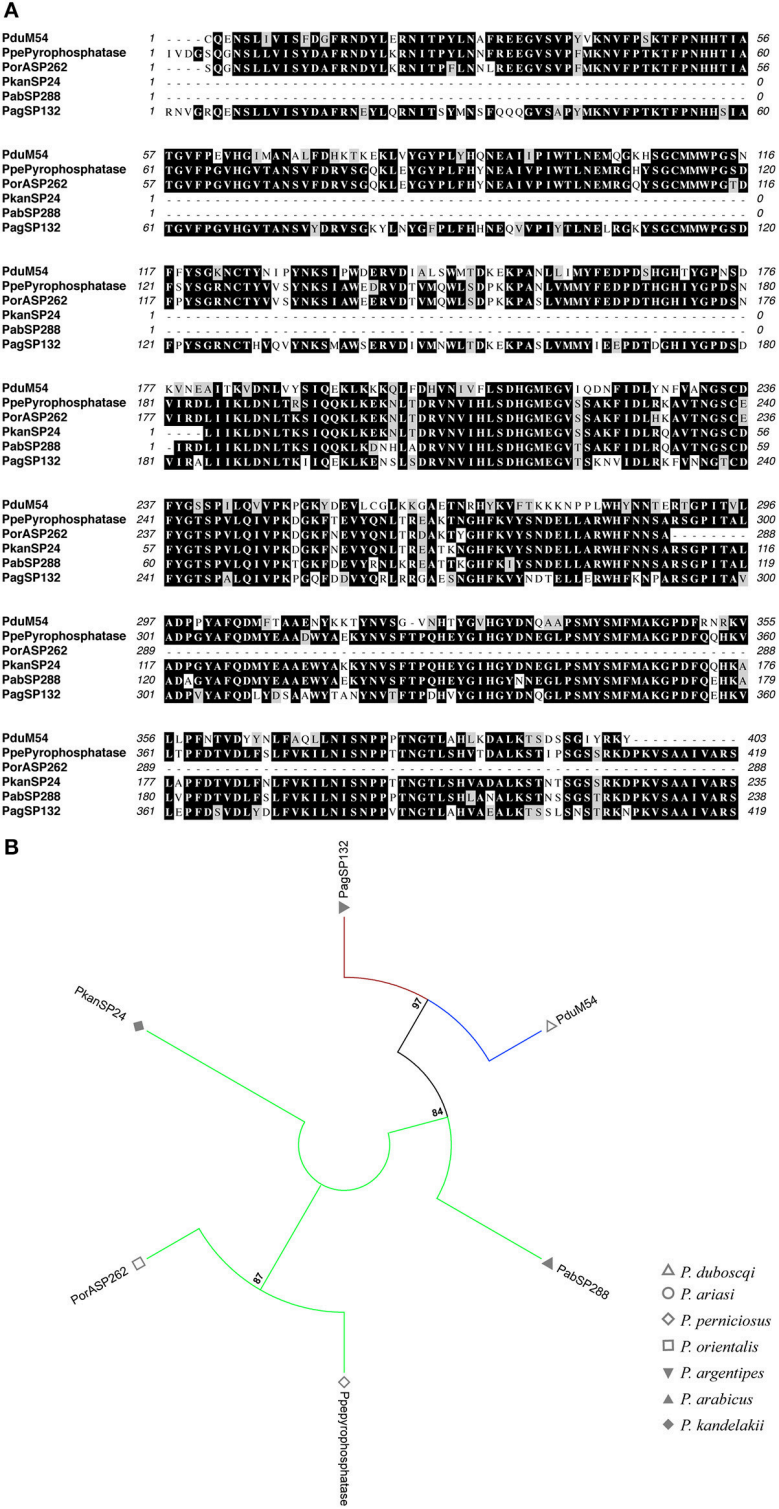
Multiple sequence alignment and molecular phylogenetic analysis of the sand fly **Pyrophosphatase** salivary protein family. **(A)** Multiple sequence alignment of **Pyrophosphatase A2 proteins**. PduM54 (*P. duboscqi*), PpePyrophosphatase (*P. perniciosus*), PorASP262 (*P. orientalis*), PkanSP24 (*P. kandelakki*), PabSP288 (*P. arabicus*), and PagSP132 (*P. argentipes*). Black background shading represents identical amino acids. Gray background shading represents similar amino acids. **(B)** The evolutionary history of **Pyrophosphatase** salivary protein family was inferred by using the Maximum Likelihood method based on the Whelan And Goldman model (Whelan and Goldman, 2001). Sand fly species are indicated by the different symbols in the legend on the right. Tree branches were color-coded so as to represent specific taxon: Green color represents the Larroussius and Adlerius subgenera; Red color indicates the Euphlebotomus subgenus; Blue color points to proteins of the Phlebotomus and Paraphlebotomus subgenera.

As important as sequence polymorphism (SNPs and INDELs) for the evolution of gene families and emergence of new molecular functions are gene duplication events (Innan and Kondrashov, 2010). Although less than two gene duplication events per species were noticed for two NW sand fly gene families (yellow-related and the small OBPs) shared with OW sand flies (Figure 6; yellow-related, Supplementary Figure 13; OBPs, Supplementary Figure 15; Abdeladhim et al., 2016), up to five gene duplication events were accounted for in six OW sand fly gene families (antigen-5, apyrase, D7, Silk, yellow-related, OBPs) shared with NW sand flies (Figure 6; antigen-5, Supplementary Figure 4; apyrase, Supplementary Figure 5; D7, Supplementary Figure 6; Silk, Supplementary Figure 12; yellow-related, Supplementary Figure 13; and OBPs, Supplementary Figure 15). In the gene families unique to NW sand flies (SALO, RGD, mannose-binding lectin, c-type lectin, and spider toxin-like) up to eight gene duplication events were detected in six gene families (Figure 6; SALO, RGD, mannose-binding lectin, c-type lectin, and spider toxin-like; Abdeladhim et al., 2016). In sharp contrast to the high rate of copy number variation in gene families unique to NW sand flies, no gene duplication event was noticed for the sand fly salivary protein encoding gene families unique to OW sand flies (Figures 6–10 and Supplementary Figures 1–3).

### Emergence of specific salivary protein encoding genes

Besides the differences in the rates of gene diversification and gene duplication events among protein families, the molecular evolution of salivary protein encoding gene families in sand flies has also displayed different modes of gene emergence as well as specific signatures of protein structure and divergence.

A few salivary gland gene families appear to have arisen upon duplication from a gene expressed in another tissue that subsequently acquired a specific promoter driving expression into the salivary gland, a mechanism called sub-functionalization (neofunctionalization) (Hahn, 2009; Innan and Kondrashov, 2010). Amongst the salivary gene families unique to NW sand flies, the c-type lectin, the mannose-binding lectin, and the spider toxin-like gene families share paralogs expressed in other tissues of sand flies and other unrelated arthropods (Abdeladhim et al., 2016). Similar phenomena were noticed for salivary protein families unique to OW sand flies, such as phospholipase A2 (Tunaz et al., 2003) and pyrophosphatase (Silva et al., 2015), as well as in protein families shared with NW sand flies, like hyaluronidase (Allalouf et al., 1975), endonuclease (Broderick et al., 2014), adenosine deaminase (Dolezelova et al., 2005), and OBPs (Benoit et al., 2017). Hence, such gene families seem to have emerged by sub-functionalization.

The presence of an OBP-like domain in the D7 protein C-termini (Hekmat-Scafe et al., 2000) points to the emergence of D7 genes by gene fusion (Kaessmann et al., 2009) between an ancient OBP and an unknown gene. In fact, conserved cysteine signatures (Table 2), as bore by OBPs, can unveil the mechanism of gene birth even among fast evolving genes. Multiple sand fly salivary protein families bear cysteine signatures (≥4 cysteines; Table 2). In the salivary protein families unique to NW sand flies, conserved cysteine residue signatures (62.5%, five out of eight protein families; Table 2) are present at a much higher frequency than in the protein families shared between OW and NW sand flies (50%, 6 out of 12; Table 2). Amongst the protein families unique to the OW sand flies, such proportion is reduced to only 28.5% of the protein families (two out of seven families; Table 2).

**Table 2:** Cysteine residue signatures of the sand fly salivary protein families.

**Protein family**	**Number of cysteine residues**	**New world sand flies**	**Old world sand flies**	**Cysteine residues signature**
SALO	6	Y	N	CX_14_CX_26−41_CX_8−11_CX_8_CX_6_C
C-Type lectin	5	Y	N	CX_70−91_CX_8−14_CX_4_CX_7_C
14.2 kDa	4	Y	N	CX_11−13_CX_18−21_CX_12−14_C
ML domain	6	Y	N	CX_13_CX_5−10_CX_42−47_CX_5−11_CX_39−43_C
Spider toxin	6	Y	N	CX_6_CX_4−9_CCX_4_CX_5_C
RGD	0	Y	N	
5 kDa	0	Y	N	
71 kDa	0	Y	N	
OBPs	5	Y	Y	CX_10_CX_3_CX_46_CX_15_CX_8_
D7 protein	10	Y	Y	CX_25−27_CX_3_CX_44−46_CX_49−50_CX_6−12_CX_3_CX_13−16_CX_9_CX_8_C
Antigen-5	14	Y	Y	CX_4_CX_9−13_CX_9−10_CX_59_CX_6_CX_5_CX_71_CX_18_CX_2_CX_15_CX_2_CX_4_CX_7_C
Yellow	4	Y	Y	CX_69−72_CX_132−136_CX_81−91_C
Endonuclease	10	Y	Y	CX_39−47_CX_9−11_CX_10−13_CX_14_CX_24_CX_192−198_CX_3_CX_25_CC
Lufaxin	5	Y	Y	CX_21−29_CX_9_CX_22−35_CX_118−125_C
Silk	0	Y	Y	
ParSP80	0	Y	Y	
ParSP17	0	Y	Y	
Hyaluronidase	0	Y	Y	
Aden. deaminase	0	Y	Y	
Apyrase	0	Y	Y	
Phospholipase A2	10	N	Y	CX_38_CX_49−51_CX_20_CCX_5_CX_23_CX_1_CX_6_CX_23_C
SP16	6	N	Y	CX_20−31_CX_8_CX_18_CCX_13_C
ParSP15	0	N	Y	
ParSP23	0	N	Y	
ParSP25	0	N	Y	
Pyrophosphatase	0	N	Y	
SP2.5	0	N	Y	

Y, protein expressed in the salivary glands.

*N, lack of expression in the salivary glands*.

### Taxon-specific expression of salivary protein encoding genes

In order to adapt to the new ecological niches, sand flies have faced the hemostasis components and immune systems of the mammal and bird species indigenous to their habitat. In order to face such selective pressures, the establishment of new protein variants may have been required for the survival of the sand flies. It is important to mention, nonetheless, that even within OW sand flies, differences in the levels of polymorphisms were noticed. The rates of codons under positive selection varied within subgroups: for instance, higher rates were noticed within the P/P subgenera in ParSP17 (Figure 5). In addition, most of the gene duplication events have taken place in *P. duboscqi* for the P/P subgenera as well as in *P. orientalis* and *P. perniciosus* for the L/A/E subgenera (Figure 6). It is noteworthy that members of the P/P subgenera only express up to three of the salivary proteins unique to OW sand flies (SP2.5, SP16, and/or pyrophosphatase) whereas members of the L/A/E subgenera for the most part secrete salivary proteins of four to five different families (ParSP15, ParSP25, ParSP23, phospholipase A2, SP16, and/or pyrophosphatase; Table 1). Among the later, ParSP15 family is unique to the Larroussius subgenus whereas phospholipase A2 is only expressed in member of Larroussius and Adlerius subgenera. Along the same lines, transcripts for hyaluronidase and endonuclease were missing in the P/P sialotranscriptomes whereas such protein are expressed in NW and L/A/E salivary glands (Table 1).

## Discussion

The comparative analysis of salivary protein gene families between OW and NW sand flies reveals that differences in the mode of evolutionary diversification amongst gene families that could have potential implications for the application of such proteins in the selection of markers of vector exposure and vaccines as: (1) At the sequence level, protein families unique to OW sand flies have evolved at a similar pace than those shared between OW and NW species, yet at a slower pace than the families unique to NW sand flies; (2) The evolutionary rates of both the protein/gene families unique to or shared between sand fly groups range from more divergent to more conserved families; (3) Gene families unique to NW sand flies have been diverging for the most part due to positive selection whereas the families shared between NW and OW (except ParSP17) as well as those unique to OW sand flies are less divergent due to purifying selection; (4) Events of gene duplication are more often observed in the gene families unique to NW sand fly species, such as SALO, RGD, mannose-binding lectin, c-type lectin, and spider toxin-like, whereas the emergence of new gene copies in OW sand flies is more evident in the gene families shared with NW sand flies, such as antigen-5, apyrase, D7, Silk, yellow-related, and OBPs; (5) Different from NW sand flies (Abdeladhim et al., 2016), the emergence of salivary gland gene families in OW sand flies relies less often on ancient genes bearing sequences encoding conserved cysteine signatures; and (6) a few salivary protein gene families is only expressed in sand flies belonging to specific sub-genera within OW sand flies; for instance, ParSP15 is unique to the Larrossious sub-genus, and SP2.5 is only expressed in members of the Phlebotomus sub-genus. Overall, these findings highlight the striking differences in the rates of molecular evolution of salivary protein encoding genes in NW and OW sand flies, which are underscored not only by sequence polymorphisms but also by copy number variation.

Also, sand fly salivary proteins are recognized by the humoral immune system of humans and animal reservoirs previously bitten by sand flies; therefore, some sand fly salivary proteins have been shown to work as biomarkers of vector exposure. For such proteins to be used as biomarker of vector exposure for the identification of taxon-specific sand fly bites, the candidates are supposed to be not only immunogenic and conserved but also expressed only within sand flies belonging to a specific taxon. For species-specific biomarkers the candidates should be divergent or with some level of homology that allows the selection of specific peptides. For specific salivary proteins to be used as vaccine components across species, we predict that the candidates need not only to be immunogenic but also conserved proteins to some extent.

Biomarkers of vector exposure have been identified among the salivary proteins of *Lu. longipalpis* (Teixeira et al., 2010), *Lu. intermedia* (Carvalho et al., 2017), *P. papatasi* (Marzouki et al., 2015), *P. perniciosus* (Drahota et al., 2014; Kostalova et al., 2017), and *P. orientalis* (Sima et al., 2016). For the most part, the best salivary protein candidates belong to conserved protein families. Although the less conserved Silk protein (Figure 3) was found to be the best biomarker of *P. papatasi* exposure (Marzouki et al., 2015), members of the apyrase, ParSP25, and antigen-5 conserved families have been shown as good markers of exposure in *P. perniciosus* (Drahota et al., 2014; Kostalova et al., 2017), *P. orientalis* (Sima et al., 2016), and *Lu. intermedia* (Carvalho et al., 2017), respectively. It is worth noting that proteins of the conserved yellow family (Figure 3) have been found to be the best biomarkers of vector exposure in dogs and humans amongst distantly related sand fly species, such as *Lu. longipalpis, P. orientalis*, and *P. perniciosus* (Teixeira et al., 2010).

Regarding sand fly salivary protein vaccines, SALO (Gomes et al., 2008) and lufaxin (Collin et al., 2009) from *Lu. longipalpis* protects hamsters and dogs, respectively, against *Leishmania infantum* infection. On the other hand, PdSP15 (PduM02 herein; OBP family) from *P. duboscqi* (and its *P. papatasi* ortholog) protects mice (Valenzuela et al., 2001) and non-human primates (Oliveira et al., 2015) against *Leishmania major* infection. As SALO is the most divergent among the unique salivary protein families in NW sand flies (Figure 3), it is more likely to work as a specific vaccine for *L. longipalpis* transmitted leishmaniasis. Regarding PdSP15 (PduM02), it belongs to the diverse OBP (super-) family, yet it only shares orthologs with *P. papatasi* and *P. sergenti* (Supplementary Figure 15). In fact, PdSP15 orthologs are relatively conserved (% divergence = 36.3%; ω = 0.54). Hence, a PdSP15-based vaccine is restricted to protect leishmania transmission from sand flies of the P/P subgenera. On the other hand, lufaxin is likely the best candidate for a pan-specific vaccine, as it is conserved and shared between OW and NW sand flies (Figure 3 and Supplementary Figure 9).

The data pointing out yellow proteins as the best biomarkers of vector exposure across sand fly species underscores the idea that conserved salivary proteins from the saliva of different sand fly species are recognized, processed, and presented in similar ways by the host immune systems. Thereby, it gives supports to the possibility that conserved yellow epitopes can be used as a pan-biomarker for sand fly exposure. For the same reasons, it is possible that conserved lufaxin epitopes can be used as pan-specific vaccines against leishmaniasis transmitted by different sand fly species.

ParSP25 proteins were shown to work as biomarkers of vector exposure for *P. perniciosus* and *P. orientalis* (Drahota et al., 2014; Sima et al., 2016; Kostalova et al., 2017). Such a protein family was among the least divergent families unique to OW sand flies (Figures 2A,B) and was only expressed in member of the L/A/E subgenera (Table 1). Thereby, it is likely that ParSP25 epitopes can be used as a taxon-specific biomarker of vector exposure to recognize bites of sand flies from L/A/E subgenera and distinguish them from bites of sand flies belonging to P/P ones that display overlapping habitats.

The evolution of salivary gland gene families unique to NW and to OW sand flies has faced completely different selective pressures, leading to a much faster pace of sequence and copy number variation in NW sand flies and a more constrained divergence in OW species. These evolutionary differences are further highlighted by the fact that the emergence of new gene families unique to NW sand flies relied upon sequences encoding specific cysteine codons, which were less evident in the genes encoding the protein families unique to OW sand flies. Regarding the protein families shared between OW and NW sand flies, the rates of sequence divergence were similar between OW and NW sand flies, yet more gene duplication events were noticed in OW species. Evolutionary differences are noticed even within OW sand flies, as more gene duplication are observed in P/P than in L/A/E, and the expression of some gene families was restricted to either P/P or L/A/E sand fly taxon.

As the salivary protein families shared between OW and NW sand flies are inter-specifically conserved, such proteins are suitable for the development of pan-specific biomarker of vector exposure and pan-specific vaccines. Along the same lines, the more conserved protein families unique to OW sand flies are ideal candidates for the development of taxon-specific biomarkers of vector exposure, as even the expression of such proteins can be restricted to a specific taxon. On the other hand, the development of species-specific biomarkers of vector exposure would require the identification of species-specific epitopes amongst the most divergent proteins families unique to either NW or OW sand flies.

## Author contributions

IC-A performed the evolutionary analysis and wrote the manuscript. JV wrote and edited the manuscript.

### Conflict of interest statement

The authors declare that the research was conducted in the absence of any commercial or financial relationships that could be construed as a potential conflict of interest.

## Supplementary material

Supplementary Figure 1 Multiple sequence alignment and molecular phylogenetic analysis of the sand fly **SP16** salivary protein family. **(Top)** Multiple sequence alignment of **SP16 proteins**. PPTSP14.3 (*P. papatasi*), PsSP98 (*P. sergenti*), PorASP152 (*P. orientalis*), PabSP63 (*P. arabicus*), and PagSP73 (*P. argentipes*). Black background shading represents identical amino acids. Gray background shading represents similar amino acids. Asterisks indicate the conserved cysteine residues. **(Bottom)** The evolutionary history of **SP16** salivary protein family was inferred by using the Maximum Likelihood method based on the Whelan And Goldman model (Whelan and Goldman, 2001). Sand fly species are indicated by the different symbols in the legend on the right. Sand fly species are indicated by the different symbols in the legend on the right. Tree branches were color-coded so as to represent specific taxon: Green color represents the Larroussius and Adlerius subgenera; Red color indicates the Euphlebotomus subgenus; Blue color points to proteins of the Phlebotomus and Paraphlebotomus subgenera.

Supplementary Figure 2 Multiple sequence alignment of the sand fly **ParSP23** salivary protein family. ParSP23 (*P. ariasi*) and PabSP56 (*P. arabicus*). Black background shading represents identical amino acids. Gray background shading represents similar amino acids.

Supplementary Figure 3 Multiple sequence alignment of the sand fly **SP2.5-like** salivary protein family. PPTSP2.5 (*P. papatasi*) and PduM80 (*P. duboscqi*). Black background shading represents identical amino acids. Gray background shading represents similar amino acids.

Supplementary Figure 4 Multiple sequence alignment and molecular phylogenetic analysis of the sand fly **Antigen-5** salivary protein family. **(Top)** Multiple sequence alignment of **Antigen-5 proteins**. PPTSP29 (*P. papatasi*), PduK107 and PduM48 (*P. duboscqi*), ParSP05 (*P. ariasi*), PorASP74 (*P. orientalis*), PpeSP07 (*P. perniciosus*), PkanSP14 (*P. kandelakki*), PabSP4 (*P. arabicus*), PagSP05 (*P. argentipes*), Luloag-5 (*Lu. longipalpis*), LayS79 (*Lu. ayacuchensis*), Linb-13 (*N. intermedia*), and LolAg5a (*B. olmeca*). Black background shading represents identical amino acids. Gray background shading represents similar amino acids. Asterisks indicate the conserved cysteine residues. **(Bottom)** The evolutionary history of **Antigen-5** salivary protein family was inferred by using the Maximum Likelihood method based on the Whelan And Goldman model (Whelan and Goldman, 2001). Sand fly species are indicated by the different symbols in the legend on the right. Tree branches were color-coded so as to represent specific taxon: Green color represents the Larroussius and Adlerius subgenera; Red color indicates the Euphlebotomus subgenus; Blue color points to proteins of the Phlebotomus and Paraphlebotomus subgenera; and Black color indicates the proteins belonging to New World sand flies.

Supplementary Figure 5 Multiple sequence alignment and molecular phylogenetic analysis of the sand fly **Apyrase** salivary protein family. **(Top)** Multiple sequence alignment of **Apyrase**. PPTSP36 (*P. papatasi*), PduM38 and PduM39 (*P. duboscqi*), PsSP42 (*P. sergenti*), PorMSP3 and PorMSP4 (*P. orientalis*), PpeSP01 and PpeSP01B (*P. perniciosus*), ParSP01 (*P. ariasi*), PtSP4 (*P. tobbi*), PkanSP09 (*P. kandelakki*), PabSP40 (*P. arabicus*), PagSP03 (*P. argentipes*), LuloApy (*Lu. longipalpis*), LayS17 (*Lu. ayacuchensis*), Linb-35 (*N. intermedia*), and LolApy (*B. olmeca*). Black background shading represents identical amino acids. Gray background shading represents similar amino acids. **(Bottom)** The evolutionary history of **Apyrase** salivary protein family was inferred by using the Maximum Likelihood method based on the Le_Gascuel_2008 model (Gomes et al., 2008). Sand fly species are indicated by the different symbols in the legend on the right. Tree branches were color-coded so as to represent specific taxon: Green color represents the Larroussius and Adlerius subgenera; Red color indicates the Euphlebotomus subgenus; Blue color points to proteins of the Phlebotomus and Paraphlebotomus subgenera; and Black color indicates the proteins belonging to New World sand flies.

Supplementary Figure 6 Multiple sequence alignment and molecular phylogenetic analysis of the sand fly **D7** salivary protein family. **(Top)** Multiple sequence alignment of **D7**. PPTSP28a (*P. papatasi*), PduK69 and Pduk103 (*P. duboscqi*), PsSP7 (*P. sergenti*), PpeSP04 and PpeSP04B (*P. perniciosus*), ParSP12 and ParSP16 (*P. ariasi*), PorMSP28 and PorMSP38 and PorMSP43 (*P. orientalis*), PtSP42 and PtSP54 and PtSP57 (*P. tobbi*), PkanSP11 and PkanSP12 (*P. kandelakki*), PabSP20 and PabSP54 and PabSP59 and PabSP84 (*P. arabicus*), LJL13 (*Lu. longipalpis*), LolD7 (*B. olmeca*), LayS101 (*Lu. ayacuchensis*), and Linb-42 (*N. intermedia*). Black background shading represents identical amino acids. Gray background shading represents similar amino acids. Asterisks indicate the conserved cysteine residues. **(Bottom)** The evolutionary history of **D7** salivary protein family was inferred by using the Maximum Likelihood method based on the Whelan And Goldman model (Whelan and Goldman, 2001). Sand fly species are indicated by the different symbols in the legend on the right. Tree branches were color-coded so as to represent specific taxon: Green color represents the Larroussius and Adlerius subgenera; Red color indicates the Euphlebotomus subgenus; Blue color points to proteins of the Phlebotomus and Paraphlebotomus subgenera; and Black color indicates the proteins belonging to New World sand flies.

Supplementary Figure 7 Multiple sequence alignment and molecular phylogenetic analysis of the sand fly **Endonuclease** salivary protein family. **(Top)** Multiple sequence alignment of **Endonuclease**. Black background shading represents identical amino acids. ParSP10 (*P. ariasi*), PpeSP32 (*P. perniciosus*), PorMSP101 (*P. orientalis*), PkanSP26 (*P. kandelakki*), PabSP49 (*P. arabicus*), PagSP11 (*P. argentipes*), LolEndo (*B. olmeca*), Linb-46 (*N. intermedia*), LJL138 (*Lu. longipalpis*), and LayS147 (*Lu. ayacuchensis*). Gray background shading represents similar amino acids. Asterisks indicate the conserved cysteine residues. **(Bottom)** The evolutionary history of **Endonuclease** salivary protein family was inferred by using the Maximum Likelihood method based on the Whelan And Goldman model (Whelan and Goldman, 2001). Branches corresponding to partitions reproduced in <50% bootstrap replicates are collapsed. Sand fly species are indicated by the different symbols in the legend on the right. Tree branches were color-coded so as to represent specific taxon: Green color represents the Larroussius and Adlerius subgenera; Red color indicates the Euphlebotomus subgenus; Blue color points to proteins of the Phlebotomus and Paraphlebotomus subgenera; and Black color indicates the proteins belonging to New World sand flies.

Supplementary Figure 8 Multiple sequence alignment and molecular phylogenetic analysis of the sand fly **Hyaluronidase** salivary protein family. **(Top)** Multiple sequence alignment of **Hyaluronidase**. PperHyal (*P. perniciosus*), PorASP112 (*P. orientalis*), PtSP125 (*P. tobbi*), PkanSP21 (*P. kandelakki*), PabSP72 (*P. arabicus*), Linb-54 (*N. intermedia*), LolHyaz (*B. olmeca*), LJLHYAL (*Lu. longipalpis*). Black background shading represents identical amino acids. Gray background shading represents similar amino acids. **(Bottom)** The evolutionary history of **Hyaluronidase** salivary protein family was inferred by using the Maximum Likelihood method based on the General Reversible Chloroplast model (Adachi et al., 2000). Sand fly species are indicated by the different symbols in the legend on the right. Tree branches were color-coded so as to represent specific taxon: Green color represents the Larroussius and Adlerius subgenera; Red color indicates the Euphlebotomus subgenus; Blue color points to proteins of the Phlebotomus and Paraphlebotomus subgenera; and Black color indicates the proteins belonging to New World sand flies.

Supplementary Figure 9 Multiple sequence alignment and molecular phylogenetic analysis of the sand fly **Lufaxin** salivary protein family. **(Top)** Multiple sequence alignment of **Lufaxin**. PPTSP34 (*P. papatasi*), PduM04 (*P. duboscqi*), PsSP49 (*P. sergenti*), PpeSP06 (*P. perniciosus*), ParSP09 (*P. ariasi*), PtSP66 (*P. tobbi*), PkanSP16 (*P. kandelakki*), PabSP32 (*P. arabicus*), PagSP05 (*P. argentipes*), Lolaxin (*B. olmeca*), Lufaxin (*Lu. longipalpis*), LayS26 (*L. ayacuchensis*), and Linb-17 (*N. intermedia*). Black background shading represents identical amino acids. Gray background shading represents similar amino acids. Asterisks indicate the conserved cysteine residues. **(Bottom)** The evolutionary history of **Lufaxin** salivary protein family was inferred by using the Maximum Likelihood method based on the Whelan And Goldman model (Whelan and Goldman, 2001). Sand fly species are indicated by the different symbols in the legend on the right. Tree branches were color-coded so as to represent specific taxon: Green color represents the Larroussius and Adlerius subgenera; Red color indicates the Euphlebotomus subgenus; Blue color points to proteins of the Phlebotomus and Paraphlebotomus subgenera; and Black color indicates the proteins belonging to New World sand flies.

Supplementary Figure 10 Multiple sequence alignment of the sand fly **ParSP17** salivary protein family. PPTSP56.6 (*P. papatasi*), PsSP82 (*P. sergenti*), PpeSP19 (*P. perniciosus*), ParSP17 (*P. ariasi*), PtSP49 (*P. tobbi*), PkanSP17 (*P. kandelakki*), PabSP53 (*P. arabicus*), LJM78 (*Lu. longipalpis*). Black background shading represents identical amino acids. Gray background shading represents similar amino acids.

Supplementary Figure 11 Multiple sequence alignment and molecular phylogenetic analysis of the sand fly **ParSP80** salivary protein family. PduK110 (*P. duboscqi*), ParSP80 (*P. ariasi*), PabSP91 (*P. arabicus*), and LJS138 (*Lu. longipalpis*). Black background shading represents identical amino acids. Gray background shading represents similar amino acids.

Supplementary Figure 12 Multiple sequence alignment and molecular phylogenetic analysis of the sand fly **Silk** salivary protein family. **(Top)** Multiple sequence alignment of **Silk**. PPTSP32 (*P. papatasi*), PduK45 and PduM33 (*P. duboscqi*), PsSP44 (*P. sergenti*), PorASP86 (*P. orientalis*), ParSP02 (*P. ariasi*), PpeSP05 (*P. perniciosus*), PtSP29 (*P. tobbi*), PkanSP15 (*P. kandelakki*), PabSP30 (*P. arabicus*), PagSP06 (*P. argentipes*), Lolsilk (*B. olmeca*), LJL04 (*Lu. longipalpis*), LayS89 (*Lu. ayacuchensis*), Linb-26 (*N. intermedia*). Black background shading represents identical amino acids. Gray background shading represents similar amino acids. **(Bottom)** The evolutionary history of **Silk** salivary protein family was inferred by using the Maximum Likelihood method based on the JTT matrix-based model (Jones et al., 1992). Sand fly species are indicated by the different symbols in the legend on the right. Tree branches were color-coded so as to represent specific taxon: Green color represents the Larroussius and Adlerius subgenera; Red color indicates the Euphlebotomus subgenus; Blue color points to proteins of the Phlebotomus and Paraphlebotomus subgenera; and Black color indicates the proteins belonging to New World sand flies.

Supplementary Figure 13 Multiple sequence alignment and molecular phylogenetic analysis of the sand fly **Yellow** salivary protein family. **(Top)** Multiple sequence alignment of **Yellow**. PPTSP42 and PPTSP44 (*P. papatasi*), PduK06 and PduM10 (*P. duboscqi*), PsSP22 and PsSP26 (*P. sergenti*), ParSP04 and ParSP04b (*P. ariasi*), PorASP2 and PorASP4 (*P. orientalis*), PtSP37 and PtSP38 (*P. tobbi*), PpeSP03B and PpeSP03 (*P. perniciosus*), PkanSP04 (*P. kandelakki*), PabSP26 (*P. arabicus*), PagSP04 (*P. argentipes*), LolYLWb and LolTLWc and LolYLWA (*B. olmeca*), Linb-21 (*N. intermedia*), LayS22 and LayS118 (*Lu. ayacuchensis*), and LJM17 and LJM17 and LJM111 (*Lu. longipalpis*). Black background shading represents identical amino acids. Gray background shading represents similar amino acids. Asterisks indicate the conserved cysteine residues. **(Bottom)** The evolutionary history of **Yellow** salivary protein family was inferred by using the Maximum Likelihood method based on the Whelan And Goldman model (Whelan and Goldman, 2001). Sand fly species are indicated by the different symbols in the legend on the right. Tree branches were color-coded so as to represent specific taxon: Green color represents the Larroussius and Adlerius subgenera; Red color indicates the Euphlebotomus subgenus; Blue color points to proteins of the Phlebotomus and Paraphlebotomus subgenera; and Black color indicates the proteins belonging to New World sand flies. Names in bold represent 44 kDa protein homologs.

Supplementary Figure 14 Multiple sequence alignment of the sand fly **Adenosine deaminase** salivary protein family. PduM73 (*P. duboscqi*), PpeAda (*P. perniciosus*), and LJLAda (*L. longipalpis*). Black background shading represents identical amino acids. Gray background shading represents similar amino acids.

Supplementary Figure 15 Multiple sequence alignment and molecular phylogenetic analysis of the sand fly **small Odorant Binding Protein (OBPs)** salivary protein family. **(Top)** Multiple sequence alignment of **OBPs**. PPTSP12 and PPTSP14 and PPTSP14.2a and PPTSP15 (*P. papatasi*), PduM12 and PduM60 and PduM07 and PduM50 and PduM57 and PduM31 and PduM49 and PduM58 and PduM62 and PduM99 and PduM02 and PduM03 and PduM06 (*P. duboscqi*), PsSP14 and PsSP14 and PsSP55 and PsSP9 (*P. sergenti*), PtSP9 and PtSP17 and PtSP32 and PtSP31 and PtSP18 and PtSP23 (*P. tobbi*), PpeSP02 and PpeSP09 and PpeSP11 (*P. perniciosus*), PorASP28 and PorASP31 and PorASP37 and PorASP61 and PorASP61 and PorASP64 (*P. orientalis*), ParSP03 and ParSP01 and ParSP08 (*P. ariasi*), PkanSP05 and PkanSP06 and PkanSP07 (*P. kandelakki*), PabSP45 and PabSP2 (*P. ariasi*), PagSP93 and PagSP07 and PagSP01 and PagSP12 and PagSP02 and PagSP13 (*P. argentipes*), LolSOBPa and LolSOBPb and LolSOBPc (*B. olmeca*), Linb-7 and Linb-8 and Linb-28 (*N. intermedia*), LayS69 (*Lu. ayacuchensis*), and LuloOBP (*Lu. longipalpis*). Black background shading represents identical amino acids. Gray background shading represents similar amino acids. Asterisks indicate the conserved cysteine residues. **(Bottom)** The evolutionary history of **OBPs** salivary protein family was inferred by using the Maximum Likelihood method based on the Le_Gascuel_2008 model (Gomes et al., 2008). Sand fly species are indicated by the different symbols in the legend on the right. Tree branches were color-coded so as to represent specific taxon: Green color represents the Larroussius and Adlerius subgenera; Red color indicates the Euphlebotomus subgenus; Blue color points to proteins of the Phlebotomus and Paraphlebotomus subgenera; and Black color indicates the proteins belonging to New World sand flies.

Supplementary Table 1 Nucleotide and protein accession numbers.

## References

[B1] Abdeladhim M. Jochim R. C. Ben Ahmed M. Zhioua E. Chelbi I. Cherni S.. (2012). Updating the salivary gland transcriptome of *Phlebotomus papatasi* (Tunisian strain): the search for sand fly-secreted immunogenic proteins for humans. PLoS ONE 7:e47347. 10.1371/journal.pone.0047347 23139741 PMC3491003

[B2] Abdeladhim M.V Coutinho-Abreu I Townsend S. Pasos-Pinto S. Sanchez L. Rasouli M.. (2016). Molecular diversity between salivary proteins from new world and old world sand flies with emphasis on *Bichromomyia olmeca*, the sand fly vector of leishmania mexicana in mesoamerica. PLoS Negl. Trop. Dis. 10:e0004771. 10.1371/journal.pntd.0004771 27409591 PMC4943706

[B3] Adachi J. Waddell P. J. Martin W. Hasegawa M. (2000). Plastid genome phylogeny and a model of amino acid substitution for proteins encoded by chloroplast DNA. J. Mol. Evol. 50, 348–358. 10.1007/s002399910038 10795826

[B4] Allalouf D. Ber A. Ishay J. (1975). Properties of testicular hyaluronidase of the honey bee and oriental hornet: comparison with insect venom and mammalian hyaluronidases. Comp. Biochem. Physiol. B 50, 331–337. 1109830 10.1016/0305-0491(75)90282-5

[B5] Anderson J. M. Oliveira F. Kamhawi S. Mans B. J. Reynoso D. Seitz A. E.. (2006). Comparative salivary gland transcriptomics of sandfly vectors of visceral leishmaniasis. BMC Genomics 7:52. 10.1186/1471-2164-7-52 16539713 PMC1434747

[B6] Aransay A. M. Scoulica E. Tselentis Y. Ready P. D. (2000). Phylogenetic relationships of phlebotomine sandflies inferred from small subunit nuclear ribosomal DNA. Insect Mol. Biol. 9, 157–168. 10.1046/j.1365-2583.2000.00168.x 10762423

[B7] Assumpcao T. C. Ma D. Schwarz A. Reiter K. Santana J. M. Andersen J. F. (2013). Salivary antigen-5/CAP family members are Cu2+-dependent antioxidant enzymes that scavenge O(2)(-). and inhibit collagen-induced platelet aggregation and neutrophil oxidative burst. J. Biol. Chem. 288, 14341–14361. 10.1074/jbc.M113.466995 23564450 PMC3656290

[B8] Barton N. H. Etheridge A. M. (2004). The effect of selection on genealogies. Genetics 166, 1115–1131. 10.1534/genetics.166.2.1115 15020491 PMC1470728

[B9] Benoit J. B. Vigneron A. Broderick N. A. Wu Y. Sun J. S. Carlson J. R.. (2017). Symbiont-induced odorant binding proteins mediate insect host hematopoiesis. Elife 6:e19535. 10.7554/eLife.19535 28079523 PMC5231409

[B10] Broderick N. A. Buchon N. Lemaitre B. (2014). Microbiota-induced changes in drosophila melanogaster host gene expression and gut morphology. MBio 5:e01117–14. 10.1128/mBio.01117-14 24865556 PMC4045073

[B11] Calvo E. Mizurini D. M. Sa-Nunes A. Ribeiro J. M. Andersen J. F. Mans B. J.. (2011). Alboserpin, a factor Xa inhibitor from the mosquito vector of yellow fever, binds heparin and membrane phospholipids and exhibits antithrombotic activity. J. Biol. Chem. 286, 27998–28010. 10.1074/jbc.M111.247924 21673107 PMC3151045

[B12] Calvo E. Tokumasu F. Marinotti O. Villeval J. L. Ribeiro J. M. Francischetti I. M. (2007). Aegyptin, a novel mosquito salivary gland protein, specifically binds to collagen and prevents its interaction with platelet glycoprotein VI, integrin alpha2beta1, and von Willebrand factor. J. Biol. Chem. 282, 26928–26938. 10.1074/jbc.M705669200 17650501 PMC2913440

[B13] Carvalho A. M. Fukutani K. F. Sharma R. Curvelo R. P. Miranda J. C. Barral A.. (2017). Seroconversion to *Lutzomyia intermedia* LinB-13 as a biomarker for developing cutaneous leishmaniasis. Sci. Rep. 7:3149. 10.1038/s41598-017-03345-0 28600554 PMC5466628

[B14] Chagas A. C. Oliveira F. Debrabant A. Valenzuela J. G. Ribeiro J. M. Calvo E. (2014). Lundep, a sand fly salivary endonuclease increases Leishmania parasite survival in neutrophils and inhibits XIIa contact activation in human plasma. PLoS Pathog. 10:e1003923. 10.1371/journal.ppat.1003923 24516388 PMC3916414

[B15] Champagne D. E. Ribeiro J. M. (1994). Sialokinin I and II: vasodilatory tachykinins from the yellow fever mosquito Aedes aegypti. Proc. Natl. Acad. Sci. U.S.A. 91, 138–142. 8278354 10.1073/pnas.91.1.138 PMC42901

[B16] Collin N. Gomes R. Teixeira C. Cheng L. Laughinghouse A. Ward J. M.. (2009). Sand fly salivary proteins induce strong cellular immunity in a natural reservoir of visceral leishmaniasis with adverse consequences for Leishmania. PLoS Pathog. 5:e1000441. 10.1371/journal.ppat.1000441 19461875 PMC2677456

[B17] Coutinho-Abreu I. V. Guimaraes-Costa A. B. Valenzuela J. G. (2015). Impact of insect salivary proteins in blood feeding, host immunity, disease, and in the development of biomarkers for vector exposure. Curr. Opin. Insect Sci. 10, 98–103. 10.1016/j.cois.2015.04.014 PMC4553692 26339571

[B18] de Castro W. Oliveira F. Coutinho-Abreu I. V. Kamhawi S. Valenzuela J. G. (2017). Basic and translational research on sand fly saliva: pharmacology, biomarkers, and vaccines, in Arthropod Vectors: Controller of Disease Transmission, ed Wikel S. K. Aksoy S. Dimopoulos G. (San Diego, CA: Academic Press), 249.

[B19] de Moura T. R. Oliveira F. Carneiro M. W. Miranda J. C. Clarencio J. Barral-Netto M.. (2013). Functional transcriptomics of wild-caught *Lutzomyia intermedia* salivary glands: identification of a protective salivary protein against *Leishmania braziliensis* infection. PLoS Negl. Trop. Dis. 7:e2242. 10.1371/journal.pntd.0002242 23717705 PMC3662654

[B20] Dolezelova E. Zurovec M. Dolezal T. Simek P. Bryant P. J. (2005). The emerging role of adenosine deaminases in insects. Insect Biochem. Mol. Biol. 35, 381–389. 10.1016/j.ibmb.2004.12.009 15804573

[B21] Drahota J. Martin-Martin I. Sumova P. Rohousova I. Jimenez M. Molina R.. (2014). Recombinant antigens from Phlebotomus perniciosus saliva as markers of canine exposure to visceral leishmaniases vector. PLoS Negl. Trop. Dis. 8:e2597. 10.1371/journal.pntd.0002597 24392167 PMC3879210

[B22] Felsenstein J. (1985). Confidence limits on phylogenies: an approach using the bootstrap. Evolution 39, 783–791. 10.2307/2408678 28561359

[B23] Gomes R. Teixeira C. Teixeira M. J. Oliveira F. Menezes M. J. Silva C.. (2008). Immunity to a salivary protein of a sand fly vector protects against the fatal outcome of visceral leishmaniasis in a hamster model. Proc. Natl. Acad. Sci. U.S.A. 105, 7845–7850. 10.1073/pnas.0712153105 18509051 PMC2397325

[B24] Hahn M. W. (2009). Distinguishing among evolutionary models for the maintenance of gene duplicates. J. Hered. 100, 605–617. 10.1093/jhered/esp047 19596713

[B25] Hekmat-Scafe D. S. Dorit R. L. Carlson J. R. (2000). Molecular evolution of odorant-binding protein genes OS-E and OS-F in Drosophila. Genetics 155, 117–127. 10790388 10.1093/genetics/155.1.117 PMC1461081

[B26] Hostomská J. Volfova V. Mu J. Garfield M. Rohousova I. Volf P.. (2009). Analysis of salivary transcripts and antigens of the sand fly Phlebotomus arabicus. BMC Genomics 10:282. 10.1186/1471-2164-10-282 19555500 PMC2714351

[B27] Innan H. Kondrashov F. (2010). The evolution of gene duplications: classifying and distinguishing between models. Nat. Rev. Genet. 11, 97–108. 10.1038/nrg2689 20051986

[B28] Jones D. T. Taylor W. R. Thornton J. M. (1992). The rapid generation of mutation data matrices from protein sequences. Comput. Appl. Biosci. 8, 275–282. 1633570 10.1093/bioinformatics/8.3.275

[B29] Kaessmann H. Vinckenbosch N. Long M. (2009). RNA-based gene duplication: mechanistic and evolutionary insights. Nat. Rev. Genet. 10, 19–31. 10.1038/nrg2487 19030023 PMC3690669

[B30] Kamhawi S. Belkaid Y. Modi G. Rowton E. Sacks D. (2000). Protection against cutaneous leishmaniasis resulting from bites of uninfected sand flies. Science 290, 1351–1354. 10.1126/science.290.5495.1351 11082061

[B31] Kato H. Anderson J. M. Kamhawi S. Oliveira F. Lawyer P. G. Pham V. M.. (2006). High degree of conservancy among secreted salivary gland proteins from two geographically distant Phlebotomus duboscqi sandflies populations (Mali and Kenya). BMC Genomics 7:226. 10.1186/1471-2164-7-226 16952314 PMC1574310

[B32] Kato H. Jochim R. C. Gomez E. A. Uezato H. Mimori T. Korenaga M.. (2013). Analysis of salivary gland transcripts of the sand fly *Lutzomyia ayacuchensis*, a vector of Andean-type cutaneous leishmaniasis. Infect. Genet. Evol. 13, 56–66. 10.1016/j.meegid.2012.08.024 23000112 PMC3873855

[B33] Kostalova T. Lestinova T. Maia C. Sumova P. Vlkova M. Willen L.. (2017). The recombinant protein rSP03B is a valid antigen for screening dog exposure to Phlebotomus perniciosus across foci of canine leishmaniasis. Med. Vet. Entomol. 31, 88–93. 10.1111/mve.12192 27718267

[B34] Kumar S. Stecher G. Tamura K. (2016). MEGA7: molecular evolutionary genetics analysis version 7.0 for bigger datasets. Mol. Biol. Evol. 33, 1870–1874. 10.1093/molbev/msw054 27004904 PMC8210823

[B35] Lerner E. A. Ribeiro J. M. Nelson R. J. Lerner M. R. (1991). Isolation of maxadilan, a potent vasodilatory peptide from the salivary glands of the sand fly *Lutzomyia longipalpis*. J. Biol. Chem. 266, 11234–11236. 2040631

[B36] Librado P. Rozas J. (2009). DnaSP v5: a software for comprehensive analysis of DNA polymorphism data. Bioinformatics 25, 1451–1452. 10.1093/bioinformatics/btp187 19346325

[B37] Martín-Martín I. Molina R. Jimenez M. (2013). Identifying salivary antigens of Phlebotomus argentipes by a 2DE approach. Acta Trop. 126, 229–239. 10.1016/j.actatropica.2013.02.008 23422341

[B38] Marzouki S. Kammoun-Rebai W. Bettaieb J. Abdeladhim M. Hadj Kacem S. Abdelkader R.. (2015). Validation of recombinant salivary protein PpSP32 as a suitable marker of human exposure to *Phlebotomus papatasi*, the vector of *Leishmania major* in Tunisia. PLoS Negl. Trop. Dis. 9:e0003991. 10.1371/journal.pntd.0003991 26368935 PMC4569422

[B39] Mondragon-Shem K. Al-Salem W. S. Kelly-Hope L. Abdeladhim M. Al-Zahrani M. H. Valenzuela J. G. (2015). Severity of old world cutaneous leishmaniasis is influenced by previous exposure to sandfly bites in Saudi Arabia. PLoS Negl. Trop. Dis. 9:e0003449 10.1371/journal.pntd.0003449 25646796 PMC4315490

[B40] Nei M. (1987). Molecular Evolutionary Genetics. New York, NY: Columbia University Press.

[B41] Nei M. K. S. (2000). Molecular Evolution and Phylogenetics. New York, NY: Oxford University Press.

[B42] Oliveira F. Kamhawi S. Seitz A. E. Pham V. M. Guigal P. M. Fischer L.. (2006). From transcriptome to immunome: identification of DTH inducing proteins from a Phlebotomus ariasi salivary gland cDNA library. Vaccine 24, 374–390. 10.1016/j.vaccine.2005.07.085 16154670

[B43] Oliveira F. Rowton E. Aslan H. Gomes R. Castrovinci P. A. Alvarenga P. H.. (2015). A sand fly salivary protein vaccine shows efficacy against vector-transmitted cutaneous leishmaniasis in nonhuman primates. Sci. Transl. Med. 7:290ra290. 10.1126/scitranslmed.aaa3043 26041707

[B44] Olson S. A. (1994). MacVector: an integrated sequence analysis program for the Macintosh. Methods Mol. Biol. 25, 195–201. 10.1385/0-89603-276-0:195 8004165

[B45] Ribeiro J. M. Vachereau A. Modi G. B. Tesh R. B. (1989). A novel vasodilatory peptide from the salivary glands of the sand fly *Lutzomyia longipalpis*. Science 243, 212–214. 2783496 10.1126/science.2783496

[B46] Rohousova I. Subrahmanyam S. Volfova V. Mu J. Volf P. Valenzuela J. G.. (2012). Salivary gland transcriptomes and proteomes of Phlebotomus tobbi and Phlebotomus sergenti, vectors of leishmaniasis. PLoS Negl. Trop. Dis. 6:e1660. 10.1371/journal.pntd.0001660 22629480 PMC3358328

[B47] Silva J. R. Amaral D. T. Hastings J. W. Wilson T. Viviani V. R. (2015). A transcriptional and proteomic survey of Arachnocampa luminosa (Diptera: Keroplatidae) lanterns gives insights into the origin of bioluminescence from the Malpighian tubules in Diptera. Luminescence 30, 996–1003. 10.1002/bio.2850 25676901

[B48] Sima M. Ferencova B. Warburg A. Rohousova I. Volf P. (2016). Recombinant salivary proteins of *Phlebotomus orientalis* are suitable antigens to measure exposure of domestic animals to sand fly bites. PLoS Negl. Trop. Dis. 10:e0004553. 10.1371/journal.pntd.0004553 26986566 PMC4795800

[B49] Teixeira C. Gomes R. Collin N. Reynoso D. Jochim R. Oliveira F.. (2010). Discovery of markers of exposure specific to bites of *Lutzomyia longipalpis*, the vector of *Leishmania infantum* chagasi in Latin America. PLoS Negl. Trop. Dis. 4:e638. 10.1371/journal.pntd.0000638 20351786 PMC2843637

[B50] Tunaz H. Park Y. Buyukguzel K. Bedick J. C. Nor Aliza A. R. Stanley D. W. (2003). Eicosanoids in insect immunity: bacterial infection stimulates hemocytic phospholipase A2 activity in tobacco hornworms. Arch. Insect Biochem. Physiol. 52, 1–6. 10.1002/arch.10056 12489129

[B51] Valenzuela J. G. Belkaid Y. Garfield M. K. Mendez S. Kamhawi S. Rowton E. D.. (2001). Toward a defined anti-Leishmania vaccine targeting vector antigens: characterization of a protective salivary protein. J. Exp. Med. 194, 331–342. 11489952 10.1084/jem.194.3.331 PMC2193460

[B52] Valenzuela J. G. Garfield M. Rowton E. D. Pham V. M. (2004). Identification of the most abundant secreted proteins from the salivary glands of the sand fly *Lutzomyia longipalpis*, vector of Leishmania chagasi. J. Exp. Biol. 207, 3717–3729. 10.1242/jeb.01185 15371479

[B53] Vlkova M. Sima M. Rohousova I. Kostalova T. Sumova P. Volfova V.. (2014). Comparative analysis of salivary gland transcriptomes of *Phlebotomus orientalis* sand flies from endemic and non-endemic foci of visceral leishmaniasis. PLoS Negl. Trop. Dis. 8:e2709. 10.1371/journal.pntd.0002709 24587463 PMC3937273

[B54] Whelan S. Goldman N. (2001). A general empirical model of protein evolution derived from multiple protein families using a maximum-likelihood approach. Mol. Biol. Evol. 18, 691–699. 10.1093/oxfordjournals.molbev.a003851 11319253

