# Supplementary material for: Comparative Evolution of Sand Fly Salivary Protein Families and Implications for Biomarkers of Vector Exposure and Salivary Vaccine Candidates
Source: Front Cell Infect Microbiol. 2018 Aug 29;8:290. doi: 10.3389/fcimb.2018.00290 (PMC6123390; doi:10.3389/fcimb.2018.00290)
Supplement: Supplementary Figure 1 — Multiple sequence alignment and molecular phylogenetic analysis of the sand fly SP16 salivary protein family. (Top) Multiple sequence alignment of SP16 proteins. PPTSP14.3 (P. papatasi), PsSP98 (P. sergenti), PorASP152 (P. orientalis), PabSP63 (P. arabicus), and PagSP73 (P. argentipes). Black background shading represents identical amino acids. Gray background shading represents similar amino acids. Asterisks indicate the conserved cysteine residues. (Bottom) The evolutionary history of SP16 salivary protein family was inferred by using the Maximum Likelihood method based on the Whelan And Goldman model (Whelan and Goldman, 2001). Sand fly species are indicated by the different symbols in the legend on the right. Sand fly species are indicated by the different symbols in the legend on the right. Tree branches were color-coded so as to represent specific taxon: Green color represents the Larroussius and Adlerius subgenera; Red color indicates the Euphlebotomus subgenus; Blue color points to proteins of the Phlebotomus and Paraphlebotomus subgenera. [file Image_1.PDF]

```
                    *                               *        *
PPTSP14.3    1 -------KPIPNDFSNFGQTLSEQINRAVNE---NLKYLPDFSNFGDTISQSVTTHIENA   50
PsSP98       1 -------KPTPNSFWDFGKMLSDNINMAVNE---GLKNLPDFSNFGTAISQSVSAHIENA   50
PorASP152    1 NDFNNFNIPYPVLPPDFGAKLSEQINRGVQESLSGLKDLEQLKDLGPKISHQIQTQVDNQ   60
PabSP63      1 -------LPKPDYWEDFGTSLSEQINRQVQQGLSGLKDLDRLKNLGSEITENVHRHVNNQ   53
PagSP73      1 ------APPNWYTSPDFGNELSKYINEQVQQSLSGLKGLDRLQNLGPEISQSVQTQLDNE   54

                       *                               *        *
PPTSP14.3   51 MWYKGANVCSTEEVLELSD-----PFPFYGY------SSTCSETNGEFICVVTENKDGKT   99
PsSP98      51 MWYKGANVCSTEEVEELSD-----PFPFYGY------SSACSETNGEFICIITENKGDKT   99
PorASP152   61 LWFRGPNVCFTETVEDVPP-----NMFYFGTSG--QFSQSCNGINDEYVCSIIETENGHT  113
PabSP63     54 MWFRGPNVCITETNEENANGESSNSIRRFGN----HISQSCIGMNEQYTCTIMDYVDGQI  109
PagSP73     55 FWFKGENVCVKEEVSEVPSYKSNVNYYYSTGTIGMQNSQICQGTNNVFTCTKFETTDGTT  114

                       **             *
PPTSP14.3  100 ERTIKKYKCCDNYSLQYDGPKLVCK----  124
PsSP98     100 QRTIKKYKCCDNYSLQYDGDKLICKETK-  127
PorASP152  114 QRKTKVYKCCQDSSLGYIGEKIRCLKNN-  141
PabSP63    110 QRKTVIYKCCENYSLGRDGEQIRCLKNDY  138
PagSP73    115 RKITKIYTCCENYSLGYNGEKKRCLKNN-  142
```

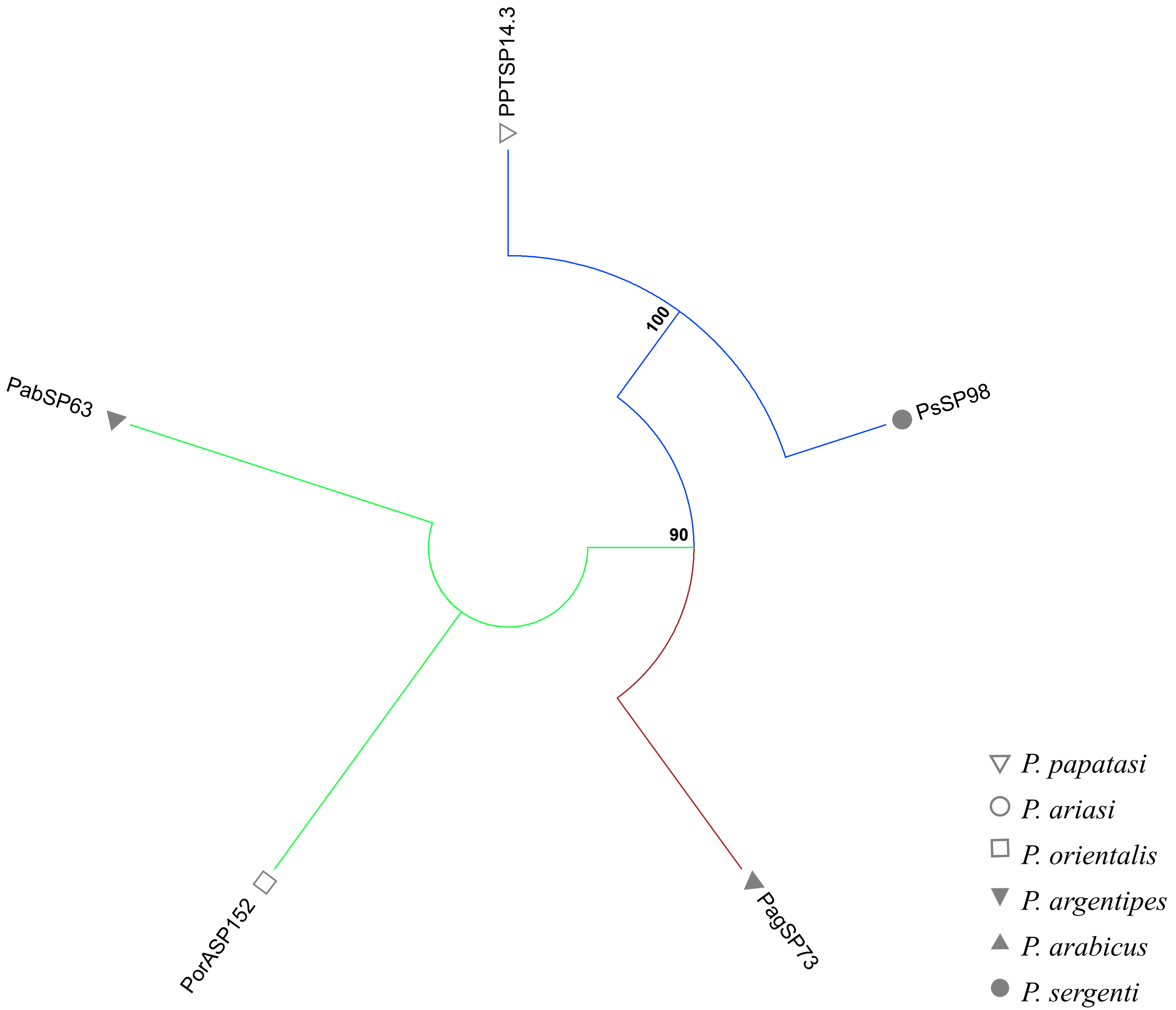

PPTSP14.3
100
PsSP98
PabSP63
90
PorASP152
PagSP73
P. papatasi
P. ariasi
P. orientalis
P. argentipes
P. arabicus
P. sergenti
