# Supplementary material for: Comparative Evolution of Sand Fly Salivary Protein Families and Implications for Biomarkers of Vector Exposure and Salivary Vaccine Candidates
Source: Front Cell Infect Microbiol. 2018 Aug 29;8:290. doi: 10.3389/fcimb.2018.00290 (PMC6123390; doi:10.3389/fcimb.2018.00290)
Supplement: Supplementary Figure 2 — Multiple sequence alignment of the sand fly ParSP23 salivary protein family. ParSP23 (P. ariasi) and PabSP56 (P. arabicus). Black background shading represents identical amino acids. Gray background shading represents similar amino acids. [file Image_2.PDF]

```
ParSP23   1  N P E K R P C T N C E - - - - R P K L S A K T P L   21
PabSP56   1  A P G K K P S Q P A K P S N Q R P P R S A R T P I   25
```
