# Supplementary material for: Comparative Evolution of Sand Fly Salivary Protein Families and Implications for Biomarkers of Vector Exposure and Salivary Vaccine Candidates
Source: Front Cell Infect Microbiol. 2018 Aug 29;8:290. doi: 10.3389/fcimb.2018.00290 (PMC6123390; doi:10.3389/fcimb.2018.00290)
Supplement: Supplementary Figure 3 — Multiple sequence alignment of the sand fly SP2.5-like salivary protein family. PPTSP2.5 (P. papatasi) and PduM80 (P. duboscqi). Black background shading represents identical amino acids. Gray background shading represents similar amino acids. [file Image_3.PDF]

**PPTSP2.5**	*1*	K Q P E K S Q T T Q K A S T T K K R P N L S V V S P L V	*28*
**PduM80**	*1*	Q S S A K A Q N N R R Q R S T T I R P N L S G R S P L M	*28*
