# Supplementary figures and images for: Comparative Evolution of Sand Fly Salivary Protein Families and Implications for Biomarkers of Vector Exposure and Salivary Vaccine Candidates
Source: Front Cell Infect Microbiol. 2018 Aug 29;8:290. doi: 10.3389/fcimb.2018.00290 (PMC6123390; doi:10.3389/fcimb.2018.00290)

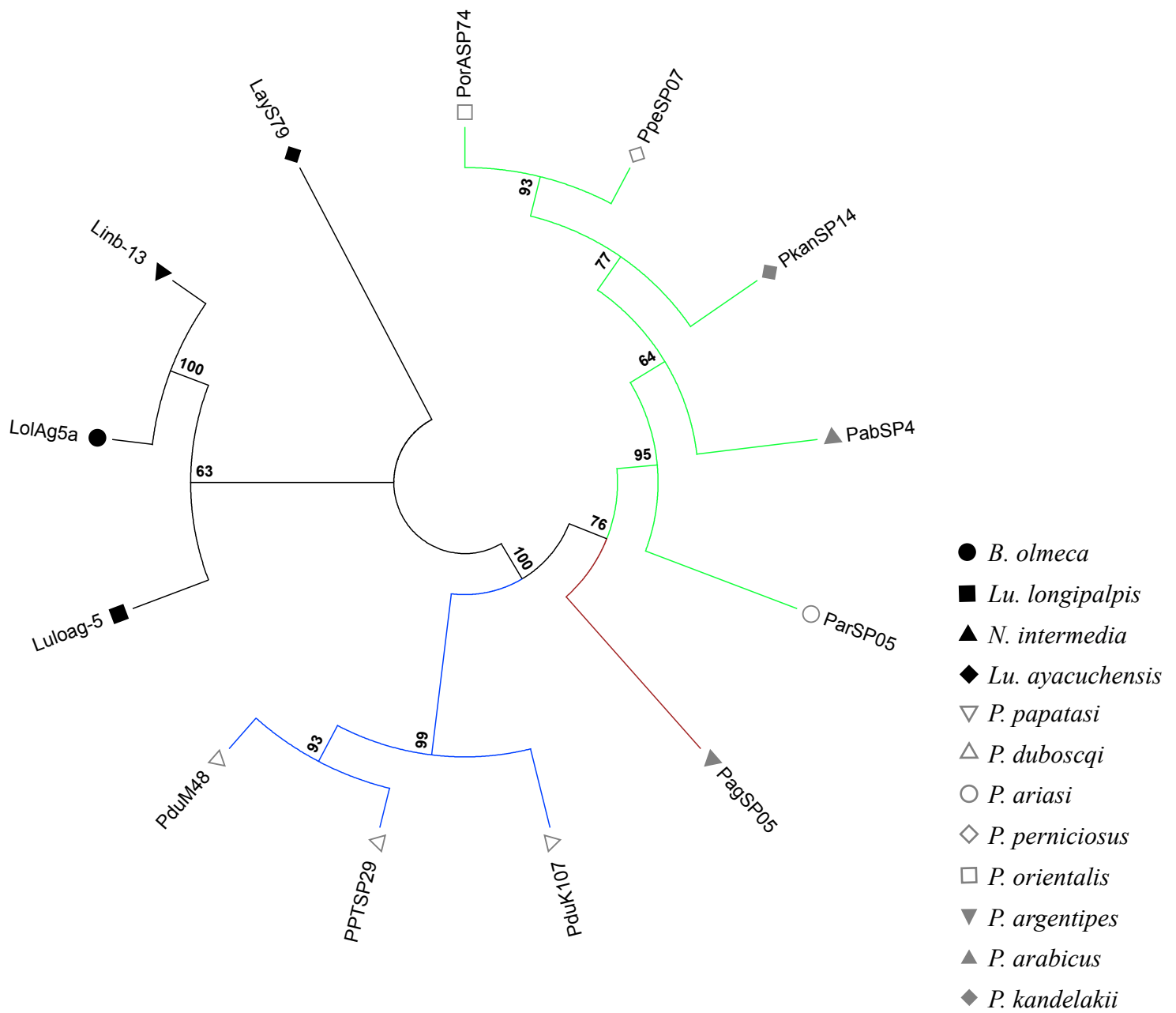

Supplement: Supplementary Figure 4 — Multiple sequence alignment and molecular phylogenetic analysis of the sand fly Antigen-5 salivary protein family. (Top) Multiple sequence alignment of Antigen-5 proteins. PPTSP29 (P. papatasi), PduK107 and PduM48 (P. duboscqi), ParSP05 (P. ariasi), PorASP74 (P. orientalis), PpeSP07 (P. perniciosus), PkanSP14 (P. kandelakki), PabSP4 (P. arabicus), PagSP05 (P. argentipes), Luloag-5 (Lu. longipalpis), LayS79 (Lu. ayacuchensis), Linb-13 (N. intermedia), and LolAg5a (B. olmeca). Black background shading represents identical amino acids. Gray background shading represents similar amino acids. Asterisks indicate the conserved cysteine residues. (Bottom) The evolutionary history of Antigen-5 salivary protein family was inferred by using the Maximum Likelihood method based on the Whelan And Goldman model (Whelan and Goldman, 2001). Sand fly species are indicated by the different symbols in the legend on the right. Tree branches were color-coded so as to represent specific taxon: Green color represents the Larroussius and Adlerius subgenera; Red color indicates the Euphlebotomus subgenus; Blue color points to proteins of the Phlebotomus and Paraphlebotomus subgenera; and Black color indicates the proteins belonging to New World sand flies. [file Image_4.PDF]

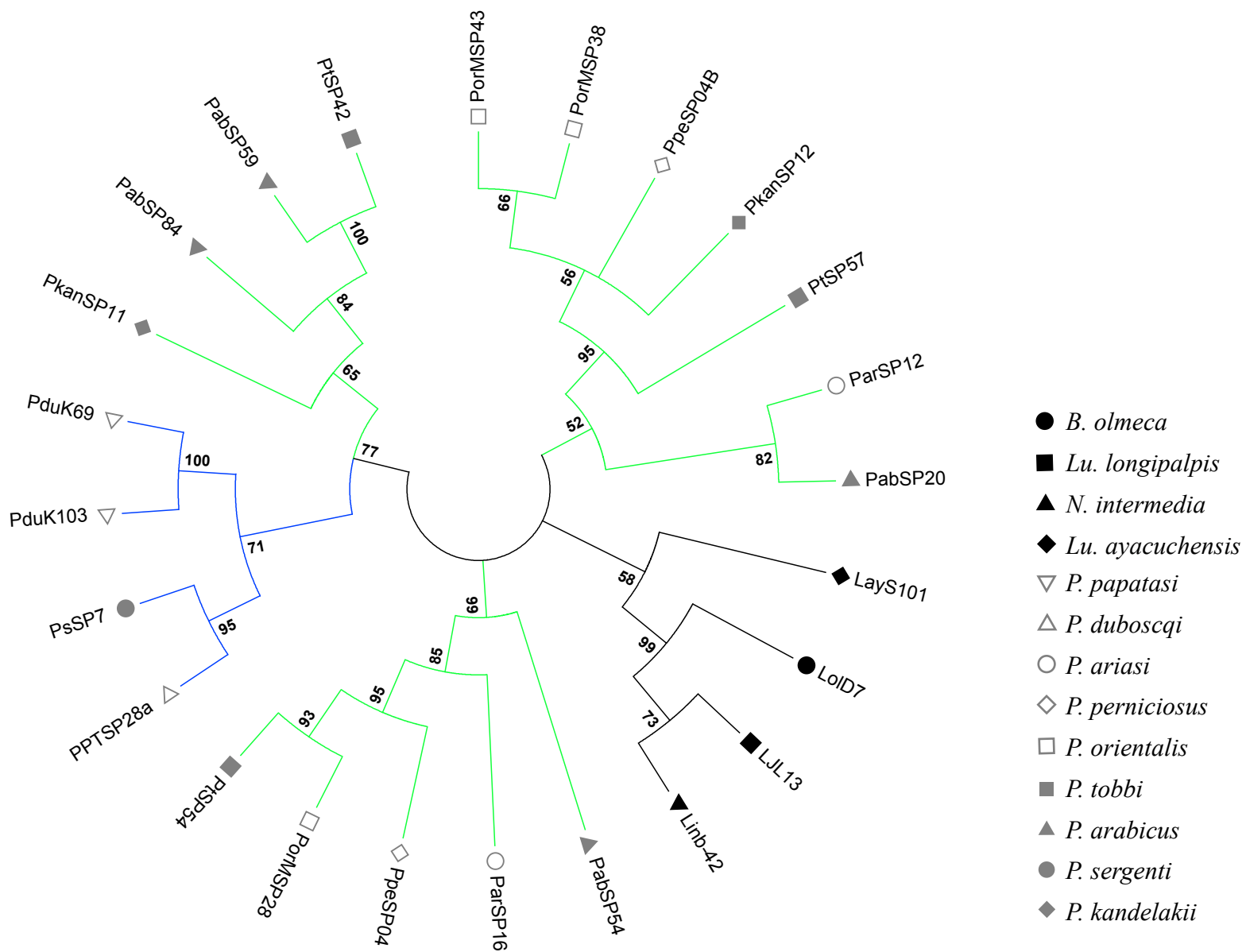

Supplement: Supplementary Figure 6 — Multiple sequence alignment and molecular phylogenetic analysis of the sand fly D7 salivary protein family. (Top) Multiple sequence alignment of D7. PPTSP28a (P. papatasi), PduK69 and Pduk103 (P. duboscqi), PsSP7 (P. sergenti), PpeSP04 and PpeSP04B (P. perniciosus), ParSP12 and ParSP16 (P. ariasi), PorMSP28 and PorMSP38 and PorMSP43 (P. orientalis), PtSP42 and PtSP54 and PtSP57 (P. tobbi), PkanSP11 and PkanSP12 (P. kandelakki), PabSP20 and PabSP54 and PabSP59 and PabSP84 (P. arabicus), LJL13 (Lu. longipalpis), LolD7 (B. olmeca), LayS101 (Lu. ayacuchensis), and Linb-42 (N. intermedia). Black background shading represents identical amino acids. Gray background shading represents similar amino acids. Asterisks indicate the conserved cysteine residues. (Bottom) The evolutionary history of D7 salivary protein family was inferred by using the Maximum Likelihood method based on the Whelan And Goldman model (Whelan and Goldman, 2001). Sand fly species are indicated by the different symbols in the legend on the right. Tree branches were color-coded so as to represent specific taxon: Green color represents the Larroussius and Adlerius subgenera; Red color indicates the Euphlebotomus subgenus; Blue color points to proteins of the Phlebotomus and Paraphlebotomus subgenera; and Black color indicates the proteins belonging to New World sand flies. [file Image_6.PDF]

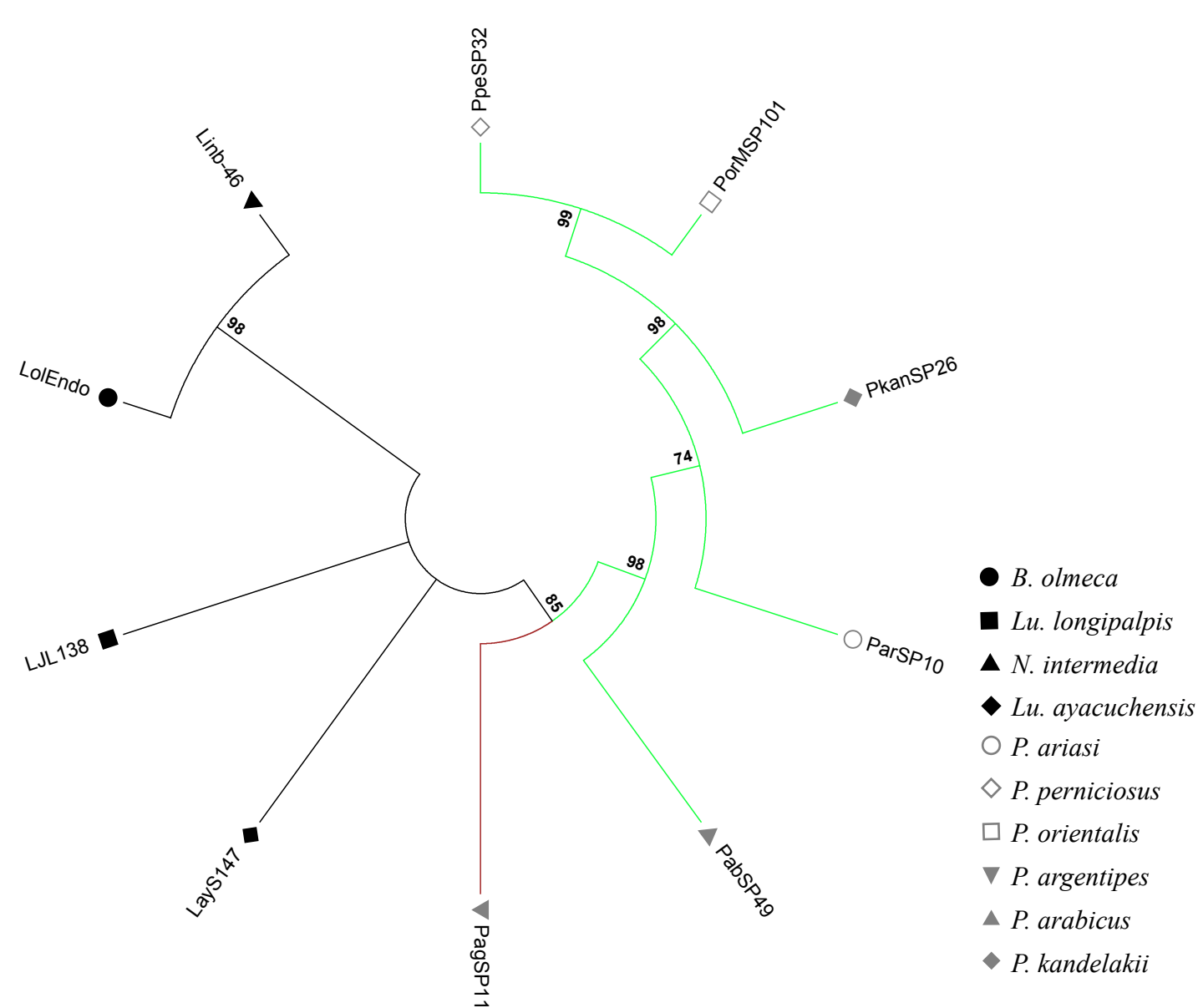

Supplement: Supplementary Figure 7 — Multiple sequence alignment and molecular phylogenetic analysis of the sand fly Endonuclease salivary protein family. (Top) Multiple sequence alignment of Endonuclease. Black background shading represents identical amino acids. ParSP10 (P. ariasi), PpeSP32 (P. perniciosus), PorMSP101 (P. orientalis), PkanSP26 (P. kandelakki), PabSP49 (P. arabicus), PagSP11 (P. argentipes), LolEndo (B. olmeca), Linb-46 (N. intermedia), LJL138 (Lu. longipalpis), and LayS147 (Lu. ayacuchensis). Gray background shading represents similar amino acids. Asterisks indicate the conserved cysteine residues. (Bottom) The evolutionary history of Endonuclease salivary protein family was inferred by using the Maximum Likelihood method based on the Whelan And Goldman model (Whelan and Goldman, 2001). Branches corresponding to partitions reproduced in <50% bootstrap replicates are collapsed. Sand fly species are indicated by the different symbols in the legend on the right. Tree branches were color-coded so as to represent specific taxon: Green color represents the Larroussius and Adlerius subgenera; Red color indicates the Euphlebotomus subgenus; Blue color points to proteins of the Phlebotomus and Paraphlebotomus subgenera; and Black color indicates the proteins belonging to New World sand flies. [file Image_7.PDF]
