# Supplementary material for: Comparative Evolution of Sand Fly Salivary Protein Families and Implications for Biomarkers of Vector Exposure and Salivary Vaccine Candidates
Source: Front Cell Infect Microbiol. 2018 Aug 29;8:290. doi: 10.3389/fcimb.2018.00290 (PMC6123390; doi:10.3389/fcimb.2018.00290)
Supplement: Supplementary Figure 5 — Multiple sequence alignment and molecular phylogenetic analysis of the sand fly Apyrase salivary protein family. (Top) Multiple sequence alignment of Apyrase. PPTSP36 (P. papatasi), PduM38 and PduM39 (P. duboscqi), PsSP42 (P. sergenti), PorMSP3 and PorMSP4 (P. orientalis), PpeSP01 and PpeSP01B (P. perniciosus), ParSP01 (P. ariasi), PtSP4 (P. tobbi), PkanSP09 (P. kandelakki), PabSP40 (P. arabicus), PagSP03 (P. argentipes), LuloApy (Lu. longipalpis), LayS17 (Lu. ayacuchensis), Linb-35 (N. intermedia), and LolApy (B. olmeca). Black background shading represents identical amino acids. Gray background shading represents similar amino acids. (Bottom) The evolutionary history of Apyrase salivary protein family was inferred by using the Maximum Likelihood method based on the Le_Gascuel_2008 model (Gomes et al., 2008). Sand fly species are indicated by the different symbols in the legend on the right. Tree branches were color-coded so as to represent specific taxon: Green color represents the Larroussius and Adlerius subgenera; Red color indicates the Euphlebotomus subgenus; Blue color points to proteins of the Phlebotomus and Paraphlebotomus subgenera; and Black color indicates the proteins belonging to New World sand flies. [file Image_5.PDF]

```
PPTSP36    1   APRSGTIYNFAIIADLDKKSISPKNDNNYKSIVKVGELIQVG--DKYSVKMKK-EDHEIF   57
PduM39     1   APSSETIYKFAIIADLDRKSISQKNDNNYKSIVKIGQLNQVG--RKFNFAMEN-KDHEIF   57
PduM38     1   APRSGTTINFAIIADLDKKSISKKNDNNYKSIVKLGELYKVA--DKYSFSMKD-EHHEVF   57
PsSP42     1   APQGGKSFNFAIIADLDKKSISKTDANNFKSIVKLGELTQVG--TKYDIVMKNKEDREIF   58
PorMSP4    1   APRATKFIPFALIADFDKKSIK-EDQKSFTSIVKYGELKHNG--ERYTLSLKS-ENLHYF   56
PtSP4      1   APRATKFIPFALIADLDKKSIK-EDQKSFTSIVKYGELKHNG--ERYTLSLKS-ENLHYF   56
PpeSP01B   1   APRATRFIPFALIADLDRKSIK-EDQKSFTSIVKYGELKDNG--ERYTLSLKS-ENLHYF   56
ParSP01    1   APRATRFIPFAVISDLDKKSIK-SDQKSFTSIVRYGELKDNG--ERYTLSIKS-ENLHYF   56
PorMSP3    1   APRPTRFIPFAIISDLHRKAMH-DEKNRFTSIVKYGQLKYNG--EKYTLSIRS-ENLHYF   56
PpeSP01    1   APRPTRFIPFAIISDLHRKAMH-DEKNRFTSIVKYGQLKYNG--EKYTLSIRS-ENLHYF   56
PkanSP09   1   APRATKFIPFAVIADLDRKSIK-DDQKSFTSIVKYGELKHNG--ERYTLSLKS-ENLHYF   56
PabSP40    1   APRATRFIPFAIVADLDKKSIK-SDQKSFTSIVKYGELKDNG--ERYTLTMKS-ENLHYF   56
PagSP03    1   APRALRFIPFAVIADLDKDSIK-DAGKQFTSIVKYGELRDNG--ENYDLTMKS-QNLHYF   56
LolApy     1   APPDVEWYHFGLIADMDKKAISKSDPTTFNSDLKIDELQHNTKTDKYTYVISR-VKKPVT   59
LuloApy    1   APPGVEWYHFGLIADMDKKSIA-SDKTTFNSVLKIDELRHNTKTDQYIYVRSR-VKKPVS   58
LayS17     1   APPGVEWYHFGLIADMDKKSISPSDRNTFNSILKIDELRHNTRTGRYNFVISR-VKKPVS   59
Linb-35    1   APPGVEWYHFGMIADMDKKSISKSEKNTFNSDLKIDELRHDVKSDRYSYVMSR-IKKPVT   59

PPTSP36    58  TKYAYKGRGAELSEFLIYKWKLYTFDDKSGIIFRLKTNADLIPWVTLANGNGDQTDGFKA   117
PduM39     58  TKYAYKGRGAELSEFLVYKWKLYTFDDKSGIVFKLKNNADLVPWVILANGNGDQVDGFKA   117
PduM38     58  TKYAYKGRGAELSEFLVYKWKLYTFDDKSGIVFKLKNNADLVPWVILANGDGDQVDGFKA   117
PsSP42     59  TKYAYRGRGAELSEFLRFNRKLYSFDDKSGIVFQLKDNADLVPWVVLANGDGNQKDGFKA   118
PorMSP4    57  TRYAYNGRGAELSELLYFNDKLYTIGDKTGIVFEVKHGGDLIPWVILSNGPGNQKDGFKA   116
PtSP4      57  TRYAYNGRGAELSELLYFNDKLYTIGDKTGIVFEVKHGGDLIPWVILANGPGNQKDGFKA   116
PpeSP01B   57  TRYAYNGRGAELSELLYFNDKLYTIGDKTGIVFEVVHGGDLIPWVILSNGPGNQKDGFKA   116
ParSP01    57  TRYAYNGRGAELSELLYFNNKLYTIDDKTGIIFEVKHGGDLIPWVILSNGDGNQKNGFKA   116
PorMSP3    57  TQDTYKGTGAEMSELIYFNNKLYTLNDETGTIYEVKHGGELIPWVTLKNDDGNDKDGFKA   116
PpeSP01    57  TKNTYKGTGADMSELIYFNDKLYTLNDETGTIYEVKHGGELIPWITLKNDDGNQKDGFKA   116
PkanSP09   57  TRYAYNGRGAELSELLYFNDKLYTIDDKTGIIFEVKHGGDLIPWVILSNGPGNQKDGFKA   116
PabSP40    57  TRYAYKGRGAELSELLYFNNKLYSIDDKTGIIFEVKHGGDLIPWVILSNGDGNQKDGFKA   116
PagSP03    57  TRFAYNGRGAELSELLNFNSKLFTVDDKTGIVFEVKYGGNLIPWVILANGNSNKQEGMKA   116
LolApy     60  TRFAYKGRGAELSEIVVFKNRLYTFDDKSGITFRLTKDGELHPWVILANGNGDSQEGYKA   119
LuloApy    59  TRYGFKGRGAELSEIVVFNNKLYTVDDKSGITFRITKDGKLFPWVILADADGQRPDGFKG   118
LayS17     60  TRFGYKGRGAELSEILIFNKQLYTFDDKTGIVFRMTKDGKLLPWVVLANGDGKQPDGFKA   119
Linb-35    60  TKYGFNGRGGELSEVVVYNNRLYTFDDKTGITFRMTKDGELHPWVILANGDGNRPDGFKA   119

PPTSP36    118 EWATTKGDKMYVGSTGISFTDK-TGKLNSNSLWIKEIDQDGKVQSLDWKEQYDKIKSAMK   176
PduM39     118 EWATTKGDKMYVGSTGISWSDS-TGKLNSNSLWIKEINQDGKVLSSNWKEYYDKMKSAMN   176
PduM38     118 EWATTKGDKMYVGSTGISWSDS-TGKLNSNSLWIKEIDQDGKVLSLNWKQYYDKMKSVMK   176
PsSP42     119 EWATAKDGKMYVGSTGISWTDK-SGIPNTSSLWIKEIDKEGRVQNKNWEKYYEAVKKAMN   177
PorMSP4    117 EWATVKDDKLYVGSTGMTFLDKRTGNISKNALWVKEINKEGEVISINWENQYKKVKDAMG   176
PtSP4      117 EWATVKDDKLYVGSTGMTFLDKRTGNISKNALWVKEIDKNGEVISINWENQYKKVKDAMG   176
PpeSP01B   117 EWATVKGDKLYVGSTGMTFLDKRTGTISTNALWVKVIDHNGEVTSINWENQYKKVKDAMG   176
ParSP01    117 EWATVKGDKLIVGSTGIPWFEEKTQSLNTYSLWVKEISKEGEVTNINWKSQYSKVKNAMG   176
PorMSP3    117 KWATVKGDKLIVGSAGMAFLDAKTMNIDRDALWVKEISESGHITNKYWDSEYKKVRDAMG   176
PpeSP01    117 KWATVKGDKLIVGSAGMAFLDAKTMNIDRDALWVKEISESGHVTNKYWDSQYKKVRDAMG   176
PkanSP09   117 EWATVKDDKLIVGSTGIAFQDAKTSTINKNALWVKEIDKKGEVTNIYWENQYKKVKNAMG   176
PabSP40    117 EWATVKGDKLIVGSTGMPWFNDKHQILDSNALWIKEISPEGEVTNINWKSQYSKVKNAMG   176
PagSP03    117 EWATRKGDKMYVGSTGLMWYNEKTKETNSDSMWVKEISRNGEVKSIDWHKQYEAVKKALG   176
LolApy     120 EWATKKGDAIYVGSTGVVFRDR-SGKLSTKALWIKKISKDGAVTSIDWTDIYQKIRNAAK   178
LuloApy    119 EWATIKDDTIYVGSTGMLK--------FTSSLWVKKITKDGVVTSHDWTDKYRKILKALN   170
LayS17     120 EWATVRNDKIYVGSTGITFKDE-KGNANTQSLWVKEITKDGSVTSHDWSQKYKKIREAMK   178
Linb-35    120 EWATIKSNTIYVGSIGVIFKDK-NGKPSPQSQWIKKISKDGTVTSEDWSAIYQKIRNAMK   178

PPTSP36    177 IPN--GFIWHEAVNWSKLKNQWVFLPRKCSDRPFDTKTEENIGCNKIIIASENFEIIKSI   234
PduM39     177 MPK--GFIWHEAVNWSKKKNQWVLLPRKCSELPFDTETEETIGCNKIIIASENFQKINSI   234
PduM38     177 IPN--GFIWHEAVNWSKLKNQWVLLPRKCSELPFDTNTEETIGCNKIIIASENFQIVRSI   234
PsSP42     178 IPN--GFVWHEAVNWSPIKKQWVFLPRKCSDLPYNTETEENIGCNEIIIADAVFKTVKSI   235
PorMSP4    177 ISS--GFVWHEAVNWSPRKNIWVFMPRKCSKQQFSAQIEENTGCNQIITANENFSDVKAI   234
PtSP4      177 ISS--GFVWHEAVNWSPRKNLWVFMPRKCTNQAFSAQIEENTGCNKIITANENFSDVKAI   234
PpeSP01B   177 MSS--GFVWHEAVNWSPRKNLWVFMPRKCSRQPFSAQIEEHTGCNQIITANENFNDVRVI   234
ParSP01    177 IPSSVGFVWHEAVNWSPRKNLWVFMPRKCTTEYFTSQVEEKTGCNQIITANEDFTQVKAI   236
PorMSP3    177 LFS--GFVWHEAVNWSPRKNVWVFIPRKCTNEPYTVRLDKNTGCNQIITANEDFSDIKTI   234
PpeSP01    177 LVS--GFVWHEAVNWSPRKNLWVFMPRKCTNEPYTVRLDKKTGCNQIITANEDFSEIKTI   234
PkanSP09   177 ITS--GFVWHEAVNWSPRKNIWVFMPRKCSNQQFTARIEENTGCNQIITANENFSDVKAI   234
PabSP40    177 IPSSVGFVWHEAVNWSPRKNLWVFMPRKCTTEYFTALVEEKTGCNKIITANEDFSQVKAI   236
PagSP03    177 MTN--GFVWHEAVTWSSHKKLWVFLPRKCTAEKYSRQIEETTGCNKIITANEDFTKVNAV   234
LolApy     179 IPN--GFIWHEAATWSDIFKKWVFMPRKCSKDPLSQDNEETTGCNKIIIADENFNDIQVF   236
LuloApy    171 MPN--GFVWHEAVTWSPFRKQWVFMPRKCSRHPFSQELEERTGCNKIVTADENFNDIQVI   228
LayS17     179 LPEQ-GFVWHEAVIWSQIRKEWIFLPRKCSHLAFTPSSEEASGCNLIITADEKFQNIKVI   237
Linb-35    179 MPN--GFVWHEAAMWSPLRKEWVFLPRKCSKDPISQENEEKTGCNKIITANENFKNIKVI   236

PPTSP36    235 QIKGKSINRAAGFSSFKFLPDSDDQILLALKTIEKDDKTATYITVIDITGRVLMPEMQIN   294
PduM39     235 DIKGTPFDPAAGFSSFKFLPDSDDQILIALKTIEKNGKTATYLTVIDITGKVLMSDKIVN   294
PduM38     235 RIKGKSIDPAAGFSSFKFLPESDDQILIALKTIEKNGKTATYLTVIDITGRVLMPDKIIN   294
PsSP42     236 QIDKNPIDSASGFSSFKFLPDTNDQILIALKTVEKGDKTATYITVIDINGKVLMPEKLIN   295
PorMSP4    235 NIDRAAVDPASGFSSFKFIPNTRNNDIFAIKTIERNGQTATYGTVIDINGKTLLPDKRIL   294
PtSP4      235 RIDRDAVDRASGFSSFKFIPNTRNNDIFAIKTIERDGKTATYGTVIDINGKTLLPDKKIL   294
PpeSP01B   235 HINRAAADSASGFSSFKFIPNTRNNDIFAIKTIERNGQTATYGTVIDINGKTLLPDQRIL   294
ParSP01    237 RIDGPVQDQAAGFSSFKFIPGTQNNDIFALKTIERNGQTATYGTVINIEGKTLLNEKRIL   296
PorMSP3    235 KIEGDIKDQASGFSSFKFIPGTKNNDIFALKTIEKNGKIATYGTVIDINGKTLMPERRII   294
PpeSP01    235 KIEGDIKDPASGFSSFKFIPGTKNNDIFALKTIEKNGKIAAYGTVIDINGKTLMPERRIL   294
PkanSP09   235 KIDKAAQDPASGFSSFKFIPGTKNNDIFALKTIERNGQTVTYGTVIDITGKTLLRDKQIL   294
PabSP40    237 RINGPVEDSASGFSSFKFIPGTQNNDILALKTIEKNGGTATYATVINIEGKTLLQEKWII   296
PagSP03    235 SITDNKNDPASGFSSFKFIPGTNNEHILAIKTIEKDGATATYAKVITLTGKTLLS-KKIL   293
LolApy     237 DIKDTPKHSASGFSAFRFIPGTYNNRILALRTIEQGKTVETSVVVINIRGRVFMNEKKLY   296
LuloApy    229 HIQDQPYNLASGFSSFRFIPGTKNERLLALRTVEQEDQVKTWAVVMDMKGTVLMYEKELY   288
LayS17     238 PVRDHPAEIASGFSAFKFIPGTNNEKLLALRTIEQGEKIATYAVVIDMEGNVLMPEPKLY   297
Linb-35    237 DIKDTPRNPASGFSTFRFIPGTNNGRILALRTVEKDDLIETSAVVIDMSGKVLMPEKKLY   296

PPTSP36    295 SDKYEGIVLLKSTEGFLKRSQ-   315
PduM39     295 KDKFEGIVLLKSTEGFLKRKE-   315
PduM38     295 EDKFEGIVLLKNTEGFLKRKE-   315
PsSP42     296 NDKYEGIELLKNAKGLFKRYAL   317
PorMSP4    295 DDNMKELHFS-GSQSIK-----   310
PtSP4      295 DDKYEGIAFFKDPKGIK-----   311
PpeSP01B   295 DDKYEGIAFFKDPKGIKLE---   313
ParSP01    297 DDKYEGVAFFKNPEGII-----   313
PorMSP3    295 DDKYEGLVFFRHPIGIK-----   311
PpeSP01    295 DDKYEGLVFFKHPAGIKKLE--   314
PkanSP09   295 DDKYEGIAFFKDPKGIK-----   311
PabSP40    297 NDKYEGVAFFKNPKGII-----   313
PagSP03    294 DTKNEGVEFMRNPQGIV-----   310
LolApy     297 DDKYEGLAFFGGVRKNKS----   314
LuloApy    289 DEKFEGIAFFGGIKKN------   304
LayS17     298 DEKYEGVAFFGGSQKK------   313
Linb-35    297 NDKYEGLAFFGGVRRNKS----   314
```

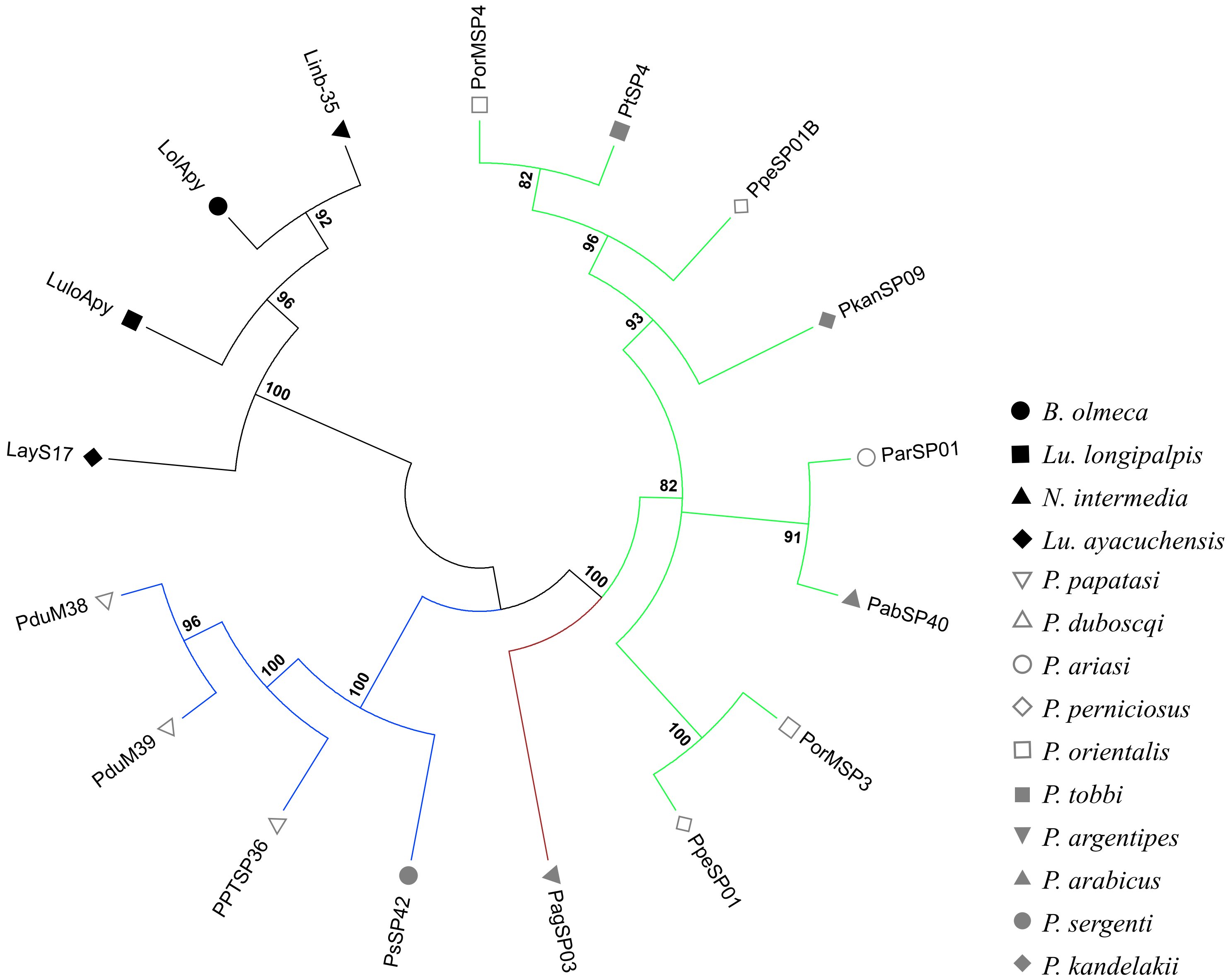

PorMSP4
PtSP4
PpeSP01B
PkanSP09
ParSP01
PabSP40
PorMSP3
PpeSP01
PagSP03
PsSP42
PPTSP36
PduM39
PduM38
LayS17
LuloApy
LolApy
Linb-35
B. olmeca
Lu. longipalpis
N. intermedia
Lu. ayacuchensis
P. papatasi
P. duboscqi
P. ariasi
P. perniciosus
P. orientalis
P. tobbi
P. argentipes
P. arabicus
P. sergenti
P. kandelakii
