# Supplementary material for: Comparative Evolution of Sand Fly Salivary Protein Families and Implications for Biomarkers of Vector Exposure and Salivary Vaccine Candidates
Source: Front Cell Infect Microbiol. 2018 Aug 29;8:290. doi: 10.3389/fcimb.2018.00290 (PMC6123390; doi:10.3389/fcimb.2018.00290)
Supplement: Supplementary Figure 8 — Multiple sequence alignment and molecular phylogenetic analysis of the sand fly Hyaluronidase salivary protein family. (Top) Multiple sequence alignment of Hyaluronidase. PperHyal (P. perniciosus), PorASP112 (P. orientalis), PtSP125 (P. tobbi), PkanSP21 (P. kandelakki), PabSP72 (P. arabicus), Linb-54 (N. intermedia), LolHyaz (B. olmeca), LJLHYAL (Lu. longipalpis). Black background shading represents identical amino acids. Gray background shading represents similar amino acids. (Bottom) The evolutionary history of Hyaluronidase salivary protein family was inferred by using the Maximum Likelihood method based on the General Reversible Chloroplast model (Adachi et al., 2000). Sand fly species are indicated by the different symbols in the legend on the right. Tree branches were color-coded so as to represent specific taxon: Green color represents the Larroussius and Adlerius subgenera; Red color indicates the Euphlebotomus subgenus; Blue color points to proteins of the Phlebotomus and Paraphlebotomus subgenera; and Black color indicates the proteins belonging to New World sand flies. [file Image_8.PDF]

```
PperHyal    1   SEILKSPERNFTIYWNVPTNQCNRHNYTSANETKPDFPTLLTNLSIVHNV--NGS--FRG   56
PorASP112   1   ----ENPEKNFTIYWNVPTDQCNRHNYT--NETKPNFPELLTNLSIVHNV--NGS--FRG   50
PtSP125     1   SETLKSPERNFTIYWNVPTDQCNRHNYT-ANETKPNFPELLTNLSIVHNF--NGS--FRG   55
PkanSP21    1   ---TEKPNKNFTIYWNVPTDQCNKHNYTNANGTKPDFAALLTNLSIVHNV--NGS--FRG   53
PabSP72     1   ----ADGERNFTIYWNVPTDQCNKHNYT--NATKPDFASLLKNLSIVQNF--KGT--FIG   50
Linb-54     1   ---------ETFKIYWNVPTHLCRNQNIS--------FDSLLQGLNIIQNKGKDGNYSFSG   44
LolHyaz     1   -----------FTIYWNVPTHLCINQNVS--------FTKLLDDLHIEQNA--DGK--FSG   38
LJLHYAL     1   ------EENSFTIYWNVPTHQCEKLNVS--------FISLLKELNIVHNK--DGN--FSG   42

PperHyal    57  EEFRILYSPGLWPSMEHNKTENGTHGGMPHHGDLEKHLEQLETDIKNCSHINYIPEHFTG   116
PorASP112   51  EEFRILYSPGLWPSMEHNKTENGTHGGMPHHGNLTEHLEQLEKDINNCSHINYIPNNFTG   110
PtSP125     56  EEFRILYSPGLWPSMEHNKTENGTHGGMPHHGNLTKHLEQLEKDIKNCSNINYIPENFTG   115
PkanSP21    54  EEFRILYSPGLWPSMEHNKTENGTHGGMPHHGNITEHLKQLEIDINNCSHINYIPKNFTG   113
PabSP72     51  EEFRILYSPGLWPSMEHNKTENGTHGGLPHRGNLTKHLEQLEADIKNCSHINYIPENFTG   110
Linb-54     45  ESFTILYSPGLWPSMEHNNKTN---GGMPHHGNLKLHLKDLKQDIEKGITN----ENYSG   97
LolHyaz     39  EKFTILYSPGLWPSMEHN-TTN---GGMPQHGNLSIHLEKLKIDIKKRIKE----ENYTG   90
LJLHYAL     43  ESFTILYSPGLWPSMEHKNITN---GGMPQCGNMTLHLEKLEKDVKEKLKD----DGYSG   95

PperHyal    117 MAVIDMESWRPVFRQNTGWMQIYRKLVFEEIDRKACQNETY------FKEILNNT-----   165
PorASP112   111 MAVIDMESWRPVFRQNTGWMEIYRKLVFREIDSNATLRN---------LVYNDSSG----   157
PtSP125     116 MAVIDMESWRPVFRQNTG------------------------------------------   133
PkanSP21    114 MAVIDMESWRPVFRQNTGWMEIYRKLVFREIDKNKTLRE--------QEKINDS-----   159
PabSP72     111 MAVIDMESWRPVFRQNTGWMGIYRKLVFQEIDRNETLLNEIKKNETEIMKKKNKNPKMLN   170
Linb-54     98  LTVIDMESWRPVFRQNSGWMTIYRNLTFKEVN--ETLTK----------ELEKTP-----   140
LolHyaz     91  LAVIDMESWRPIFCQNTGWMIKYRYLTFEEVN--KTLAD----------EFQKNP-----   133
LJLHYAL     96  LAVIDMESWRPVFRQNTGWMIKYRELTFEQYN--KTLAE----------EYKNNT-----   138

PperHyal    166 -TCSGNKANVCFKEAAKIFEPMAIDYMNKSIAKVRELRPEAHWGYYGFPYCFNI--RKND   222
PorASP112   158 ITCEKNRTNLCFKEAAKIFEPMAIDYMNKSIAKLRELRPEAHWGYYGFPYCFNI--RKDN   215
PtSP125     134 ------------------------------------------------------------   133
PkanSP21    160 ITCEKNRTSDCFKVAADIFEPMAIDFMNKSIAKVRELRSEAHWGYYGFPYCFNI--RKND   217
PabSP72     171 FTCLKNRTNSCFKVAAKIFEPMAIRFMNESISKLKELRPQAKWGYYGFPYCFNI--RKDN   228
Linb-54     141 --TNTEIRNYLIKEGAKIFEPKAKEFLNESTLLVKDLRKSAKWGYYGFPYCFNMGQALSA   198
LolHyaz     134 --KNTTLRNHLMKEGAKIFEPKAKEFLTKSTELVKKERSKAKWGYYGFPYCFNMGQAKSA   191
LJLHYAL     139 --TNDKLRNRLIKESAEIFEAPAKDFLMNSTELVKKYWKDAKWGYYGFPYCFNMGVAANA   196

PperHyal    223 RNESCPKPVPKENNNTAWLFQSYDSWYPSVYISHDNFTEEDRQNLVSGRVKEYNRLRNLT   282
PorASP112   216 RSESCAELVQKENDNTTWLFQSYNSWYPSVYISHDNFTEEDRLKLVRGRVQEYNRLRNLT   275
PtSP125     134 ------------------------------------------------------------   133
PkanSP21    218 RNESCSKRVQKENNETAWLFKSYDSWYPSVYISHDNFTEDDRLNLVRGRVQEYNRLRKLH   277
PabSP72     229 RTEDCSSLVQRENNNTEWLFTSYEHWYPSVYISHDNFTVDDRLKLVRGRVKEYNRLRDTF   288
Linb-54     199 RNETCPTI----------------------------------------------------   206
LolHyaz     192 RNENCPDIVQKENNKTDWLFKSYDYWFPSVYISSVNFTADERLQLVRGRTTEYNRLRDLF   251
LJLHYAL     197 RNESCPKIVKEENNKTEWLFKSYDYWFPSVYITKVNFTCEERGQLVRGRVTEYQRLRKEF   256

PperHyal    283 NQN--ATIYPYVWLLYNLDNRTEVYLNETDLNMTLTTLKNYTMDGAVIWGMSQNVNTSDK   340
PorASP112   276 NQN--ATIYPYIWLLYNLDNRTEVYLNKTDLNMTLTTLKIT-------------------   314
PtSP125     134 ------------------------------------------------------------   133
PkanSP21    278 NPN--ATIYPYVWLLYNLPNPKDVYLNQTDLNMTFTTLKNYTMDGAVIWGMSQNVNTSEK   335
PabSP72     289 NLTDKTKIYPYVWLLYNLDNRTEVYLNGSDLNMTLTTLKNYTMDGAVIWGMSQNVNTSGK   348
Linb-54     207 ------------------------------------------------------------   206
LolHyaz     252 NQN--AKIYPYVWYLYNLEN---KYLNETDLRMTLKTLKDNKMDGTCDLGFQ--------   298
LJLHYAL     257 NPK--AKIYPYVWFLYNLSN---EYLSKEDLEMSLKILKMGKMDGAVIWGSSKNLTKECE   311

PperHyal    341 CLKLYKYVNETLKPILEGLNITRHEPKSNGTGSILESQKCPKNTTNTTNNTNQRKKREIK   400
PorASP112   315 ------------------------------------------------------------   314
PtSP125     134 ------------------------------------------------------------   133
PkanSP21    336 CIKLYEYVNETLGPILKEIGINKTEPISNTT--ILESQKCPIN-----NTINKRQKREIK   388
PabSP72     349 CTALFNYVNETLRPILSGLKISQSE--SKADNETIASQKCPKNTTG--KGISTRKKRSIE   404
Linb-54     207 ------------------------------------------------------------   206
LolHyaz     299 ------------------------------------------------------------   298
LJLHYAL     312 CKDLYDYVNGTMRTVLEGL---------------------------------KKPENNVK   338

PperHyal    401 EDAQCQNCLINDEPASPASNSDHSNQDSSIFFVKYLFQVSYNFFQSVFSQI---   451
PorASP112   315 ------------------------------------------------------   314
PtSP125     134 ------------------------------------------------------   133
PkanSP21    389 EDAQCQNCLNDDTHDT----ASNSNLDSSISVLKYVFQFSYELFQSVFIRIQPI   438
PabSP72     405 RDAKCLNCINVG-PDEKLSTTYTSITDSSTYVVKYLLKITYNVFLLVFNQYE--   455
Linb-54     207 ------------------------------------------------------   206
LolHyaz     299 ------------------------------------------------------   298
LJLHYAL     339 WNGSCQ------------------ETSDAARYLKNCTDKQ--------------   360
```

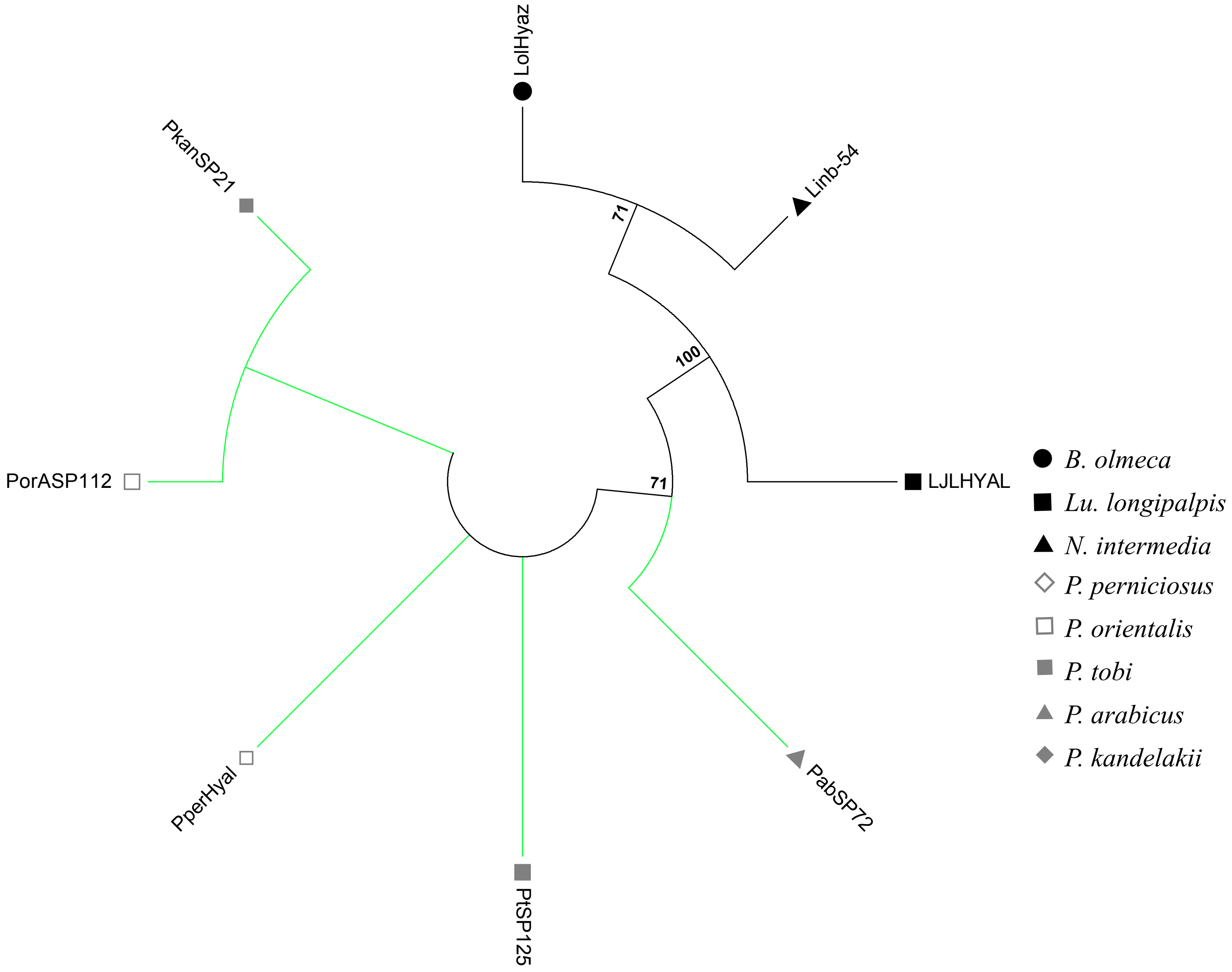
LolHyaz
Linb-54
PkanSP21
PorASP112
LJLHYAL
PperHyal
PtSP125
PabSP72
71
100
71
B. olmeca
Lu. longipalpis
N. intermedia
P. perniciosus
P. orientalis
P. tobi
P. arabicus
P. kandelakii
