# Supplementary material for: Comparative Evolution of Sand Fly Salivary Protein Families and Implications for Biomarkers of Vector Exposure and Salivary Vaccine Candidates
Source: Front Cell Infect Microbiol. 2018 Aug 29;8:290. doi: 10.3389/fcimb.2018.00290 (PMC6123390; doi:10.3389/fcimb.2018.00290)
Supplement: Supplementary Figure 9 — Multiple sequence alignment and molecular phylogenetic analysis of the sand fly Lufaxin salivary protein family. (Top) Multiple sequence alignment of Lufaxin. PPTSP34 (P. papatasi), PduM04 (P. duboscqi), PsSP49 (P. sergenti), PpeSP06 (P. perniciosus), ParSP09 (P. ariasi), PtSP66 (P. tobbi), PkanSP16 (P. kandelakki), PabSP32 (P. arabicus), PagSP05 (P. argentipes), Lolaxin (B. olmeca), Lufaxin (Lu. longipalpis), LayS26 (L. ayacuchensis), and Linb-17 (N. intermedia). Black background shading represents identical amino acids. Gray background shading represents similar amino acids. Asterisks indicate the conserved cysteine residues. (Bottom) The evolutionary history of Lufaxin salivary protein family was inferred by using the Maximum Likelihood method based on the Whelan And Goldman model (Whelan and Goldman, 2001). Sand fly species are indicated by the different symbols in the legend on the right. Tree branches were color-coded so as to represent specific taxon: Green color represents the Larroussius and Adlerius subgenera; Red color indicates the Euphlebotomus subgenus; Blue color points to proteins of the Phlebotomus and Paraphlebotomus subgenera; and Black color indicates the proteins belonging to New World sand flies. [file Image_9.PDF]

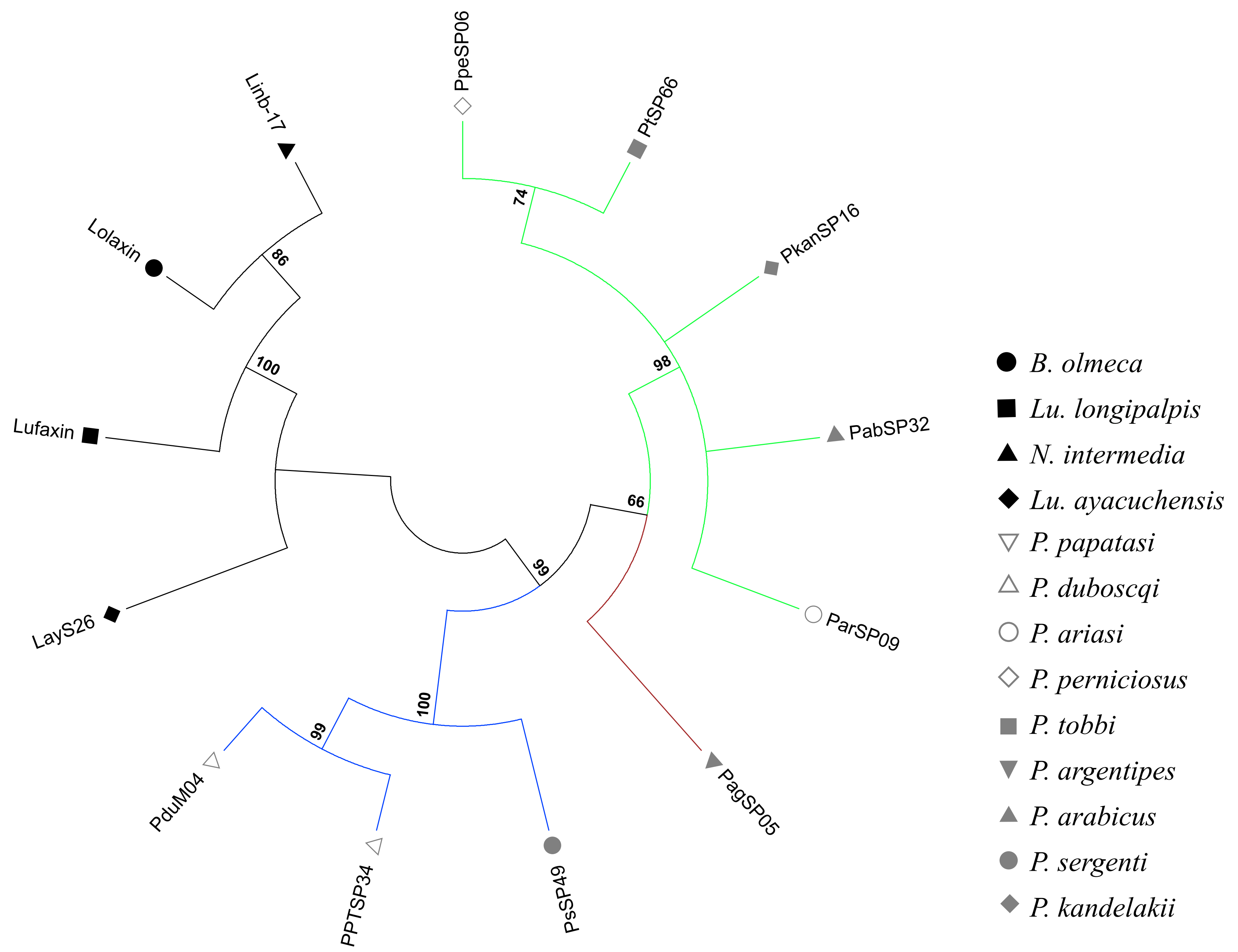

PpeSP06
PtSP66
PkanSP16
PabSP32
ParSP09
PagSP05
PsSP49
PPTSP34
PduM04
LayS26
Lufaxin
Lolaxin
Linb-17
74
98
66
99
100
99
86
100
B. olmeca
Lu. longipalpis
N. intermedia
Lu. ayacuchensis
P. papatasi
P. duboscqi
P. ariasi
P. perniciosus
P. tobbi
P. argentipes
P. arabicus
P. sergenti
P. kandelakii
