# Supplementary material for: Comparative Evolution of Sand Fly Salivary Protein Families and Implications for Biomarkers of Vector Exposure and Salivary Vaccine Candidates
Source: Front Cell Infect Microbiol. 2018 Aug 29;8:290. doi: 10.3389/fcimb.2018.00290 (PMC6123390; doi:10.3389/fcimb.2018.00290)
Supplement: Supplementary Figure 10 — Multiple sequence alignment of the sand fly ParSP17 salivary protein family. PPTSP56.6 (P. papatasi), PsSP82 (P. sergenti), PpeSP19 (P. perniciosus), ParSP17 (P. ariasi), PtSP49 (P. tobbi), PkanSP17 (P. kandelakki), PabSP53 (P. arabicus), LJM78 (Lu. longipalpis). Black background shading represents identical amino acids. Gray background shading represents similar amino acids. [file Image_10.PDF]

```
PPTSP56.6    1  VFFPVPIITGT-TSTSSSGQPTT-VNTNISFQNVSNITDMVIYLTQNISRALLVSLPTPE   58
PsSP82       1  VFFPVPIITGT-TSTSSSGQPTT-VNTNISFQNVSNITDMVIYLTQNISRAMLTSLPTPD   58
PpeSP19      1  IFFPIPINIQTGTTSSSSGQPGQQVTTSISFSNVSNITDMVIYLTQNISRALLTRVPNPD   60
ParSP17      1  ---------------------------------------MVIYLTQNISRALLTLLPNPE   21
PtSP49       1  IFFPIPINIQTGTTSSSSGQPGQQVTTSISFNNVSNITDMVIYLTQNISRALLTRLPNPD   60
PkanSP17     1  IFFPIPINIQTGTTSSS-GQPGQQVTTSISFSNVSNITDMVIYLTQNISRALLTRLPNPE   59
PabSP53      1  VFFPIPINIQTGTTSSSSGQPGQQVTTSINFSNVSNITDMVIYLTQNISRALLTHLPDPE   60
LJM78        1  ------------------------LGLPEQFKGLEDLP------KKPLAETYYHEGLNDG   30

PPTSP56.6   59  DIELVAEILDTFSDGLKSMISESKEDYEENNATGSEWITDESSNKPNFFDDIVKDINSMF  118
PsSP82      59  DIETVTDILDTFSNGLKSMISETREEYGEEPSLGFEWITDESSNNPNYFQNMLEQIQNIF  118
PpeSP19     61  DIKSAADILESFTGSLKYFQTPPDDVDQEE--------SETKSRSKRSFTDIFKQSS---  109
ParSP17     22  DVRSAADVLESFTDDLKSFYPPPDDVNEEV--------SETESRTKRSLIEQLKESQ---   70
PtSP49      61  DIKSAADVLESFTDSLKSYQTPYNDVDEEE--------SETKSRSKRSLSDILKESA---  109
PkanSP17    60  DIKSAADVLESFTESLKYFHPPSDDVQED---------SVTKSRSRRSLSDILKESA---  108
PabSP53     61  DIESAADILEKFTEDLTNSYVTFDDANEEA--------SVTKSRSKRSFLDQLKESQ---  109
LJM78       31  KTDEMVDIFKSLSDEFK-FSDENLDVGEEK------------NYKKRDITQNSVARN---   74

PPTSP56.6  119  QNFAALFNPNFDNQKNKEDTN---------------------------------------  139
PsSP82     119  HNLSTLFNQNFNNQKNKEDTNNSEDKTDDASRSEDTSKLEEKNVRRKRGLFGPTNFNLTE  178
PpeSP19    110  --------PLKEIGERIEEIK---------------------------------------  122
ParSP17     71  --------PLKQIRETVAETT---------------------------------------   83
PtSP49     110  --------PLREIREKVEDIK---------------------------------------  122
PkanSP17   109  --------PLKEIRGKIEDIT---------------------------------------  121
PabSP53    110  --------PLKAIRERVDEVK---------------------------------------  122
LJM78       75  ---------FLSNVKGIPSMP---------------------------------------   86

PPTSP56.6  140  --------------KFIPVTNKQEEN--INNQTNSNTEDQTQTTESQPEEISNLITGTRR  183
PsSP82     179  TFKIVKDRIDNFGQKLISLFNKPSEKPQSTNETNSDTKDQSQTSESQSQATNNSAAQSRR  238
PpeSP19    123  ---------------KKLKGMLKPKPQTPSGNQTDSSN----TTSETQ---------SRK  154
ParSP17     84  ---------------KYLKGFLKTKP---SGNQTESSNS---TSTKTQ---------SRK  113
PtSP49     123  ---------------KKLKGLLKPRPQTPSGNQAESSNT---TTSETQ---------SRK  155
PkanSP17   122  ---------------KKLKELLKPKPQTPSGNQTESTNTT--NTEETQ---------SRK  155
PabSP53    123  ---------------KQLQAYLKPKPQTPSGNQTDTNTT---TTETQ----------SRR  154
LJM78       87  ---------------SLPSMPSMPSIPSLWSSQTQAAPNTALALPESD------------  119

PPTSP56.6  184  KRDLTSSIPEILKTSAGLNLATN----PLYPSG---SVSADSQASFPN--LRNIFP--MS  232
PsSP82     239  KRDLFSSF--MPKVSAGLSLITN----PQNFSG---SASADSQASPPDQSLLDVFLKDIT  289
PpeSP19    155  KRALTDFIP-MDSLKDAISKTGE----VLIPS------SASAN-SSPLD-----FMSKLS  197
ParSP17    114  RRGLTDFIP-VNSLKDAISQATSGAMKAFKPS------SENKTSSNPLD-----FLASLS  161
PtSP49     156  KRALTDFIP-IDSLRDAISKTGD----VFKPS------SASANNSSPLD-----ILSKLS  199
PkanSP17   156  KRGLTDFIP-MDSLKDAMSQASEGAMKVFKPP------SEGGNASSPLD-----FLANLS  203
PabSP53    155  KRGLSDYIP-VNSFKDALSQASAAAGKMMKPPNASLSANASASGSNPLD-----FLTKIS  208
LJM78      120  -YSLLDMPN---IVKNFLKETRD-----------------------------------LY  140

PPTSP56.6  233  EIVGDLSNQTLQAMNEGIKQMAQQDAAAQNIAIQNAIQQGKELINQEMEKTKAIQQFVNT  292
PsSP82     290  EIGAAVLKETVNAMNEGVKQMAIQNAAAQGAAVQNASQQQNELIKQEINKTIFIQQYVNA  349
PpeSP19    198  DIANDLIQNSMKEISE---NLASSVAMYQVNSQLDAIKQSMDIIKQEIDKTQKIQKYVKE  254
ParSP17    162  DISRDLVQNSIKEVSG---NLVSSVALYQVNSKLDAIKQSIGIINQEIDRTKKVQQYVMN  218
PtSP49     200  DIGSDLIQKSIKEISG---NLASSIALYQVNSQFDAIKQSVDILKQEIDKTKNIQGYVTD  256
PkanSP17   204  DIASDLIQKSTKEISE---NLASSVALYQVNSQLDAINKSIDIIKQGIDSTEKIQKYVME  260
PabSP53    209  DIGRDLVQNSIKEIAS---NMASSAALYQVNSKLDAIKQSVNIIQQEIEKTKKVQEIINQ  265
LJM78      141  NDVGAFLKAITEALTN------RSSSSQLLSSPMVSTNKTKEFIRNEIQKVRKVRNFVQE  194

PPTSP56.6  293  SLDKISKLIETLAQKIRDSTCVQQFTSIRNLLSEGITCVKNKFDTGMKTINDTLSNIFDA  352
PsSP82     350  TLNKINNIIETLAQKIKSSNCIKQFTDMRNLLSEGITCVKNKFDTGMKTFNDTLSNISDA  409
PpeSP19    255  ALNQAKNATKSLGEKLKSSNCFAQFINPFKLFEKGITCVKNKIDNGLKIAKDTFKNLQQA  314
ParSP17    219  ALQQASNITNSIGEQLKSNNCFAQFINPFKLFEEVITCVKNKIENGLKIAEETFKNLNQA  278
PtSP49     257  ALNQAKDAIKSLGQKLKSSNCFAQFTDPRKLFEKGITCVKNKIDNGLQIASDTFKNLQQA  316
PkanSP17   261  ALKQAENATKSLGEKL-SNKCFAQFINPLKFFEKGITCVKNKINNGLKIAKDTFNNLQLA  319
PabSP53    266  ALQQAGAATSSFGDKVTSSNCFAQFINPFKLFEGGITCVKNKFDKGIKIATDTLNNISQA  325
LJM78      195  TLQKIRDISAAIAKKVKSSECLSNLTDIKGLVSDGINCLKEKFNDGKRIILQLYNNLLKG  254

PPTSP56.6  353  LEVPWDIKNEAEKCIENQEANALSKILCYVTIPLKLEENKLFLPVEFGKRIAEAVQFFAT  412
PsSP82     410  IEIPWDIQKEVAKCTENEDANTLSKILCYALVPLQLEENKIFLPIEFGKRIAETIQFFAT  469
PpeSP19    315  MSVPSDIQSEVSKCSQNQQLNPIAKLLCYLRTPLQLDDEKLLLPFEFTRRIREITNYFAT  374
ParSP17    279  LSVPSDIVSEVSKCSQNQNLNPLTKLLCYLRVPLQLDEEKLLLPIEFARRIREITNYFAT  338
PtSP49     317  LSVLSDIGSEVSKCSQNQQLNPTTKLLCYLRTPLQLDDEKLLLPIEFTKRIREITNYFAT  376
PkanSP17   320  FSVPSDIGSEVSKCSQNQDLNPIAKLLCYLRVPLQLDDDKLLLPIEFSRRIREITNYFAT  379
PabSP53    326  LSVPSDIKTEVSKCSDNKELNPFTKLLCFLKTPLQLDEEKLLLPIEFVRRIREITEYFAT  385
LJM78      255  LKIPNDLMVELKKCDTNQ-NNTLGRIICYFLTPLQLEKEQILLPVEFIKRILELTHYFST  313

PPTSP56.6  413  LRMDLIKCGIVTIQSTALKAVDCGKEAIIIGKDTILEMI  451
PsSP82     470  LRMDLIKCGIITIQSIAYKAVDCGKEAIIIGKDTILENF  508
PpeSP19    375  MRMDLIRCGIETIQSIGDKVEDCAREAILAVKDTLKG--  411
ParSP17    339  MRMDLIQCGIATIQSIGDKVENCAIEAILAVKDTLKG--  375
PtSP49     377  MRMDLIRCSIETIQSIGDXVERLCREAILAVKTL-----  410
PkanSP17   380  MRMDLIRCGVETIQSIGDKVEDCAREAILAVKDTLKG--  416
PabSP53    386  MRMDIIQCGIATIHSIEDKVADCAKEAILAVKDTLMG--  422
LJM78      314  MKEDLINCGITTIASIT----------------------  330
```
