# Supplementary material for: Comparative Evolution of Sand Fly Salivary Protein Families and Implications for Biomarkers of Vector Exposure and Salivary Vaccine Candidates
Source: Front Cell Infect Microbiol. 2018 Aug 29;8:290. doi: 10.3389/fcimb.2018.00290 (PMC6123390; doi:10.3389/fcimb.2018.00290)
Supplement: Supplementary Figure 11 — Multiple sequence alignment and molecular phylogenetic analysis of the sand fly ParSP80 salivary protein family. PduK110 (P. duboscqi), ParSP80 (P. ariasi), PabSP91 (P. arabicus), and LJS138 (Lu. longipalpis). Black background shading represents identical amino acids. Gray background shading represents similar amino acids. [file Image_11.PDF]

```
PduK110      1   QTCTNAQVKGATSYSTSDATIVSQIAFVTEFSLECSNVATERVSLFAEIDGRITPVAIIG   60
ParSP80      1   ETCSNPQVKGASSYTTTDATIVSQIAFITEFSLECSNPGAEKVSLFAEVDGRITPVAVIG   60
ParSP80.2    1   ETCSNAQVKGATSYTTTDATIVSQIAFVTEFSLECSNPGAEKVSLFAEVDGRITPVAVIG   60
LJS138       1   ETCSNAKVKGATSYSTTDATIVSQIAFVTEFSLECSNPGSEKISLFAEVDGKITPVAMIG   60

PduK110     61   DTKYQVSWYEEVKKARSGDYNVKLYDEEGYAAVRKTQRSGEDNNVKPLATVVVRHSGSYT  120
ParSP80     61   DTKYQVSWNEEVKKARSGDYNVRLYDEEGYGAVRKAQRSGEENNAKPLATVVVRHSGSYT  120
ParSP80.2   61   DTKYQVSWNEEVKKARSGDYNVRLYDEEGYGAVRKAQRSGEESNVKPLATVVVRHSGSYT  120
LJS138      61   DTTYQVSWNEEVNKARSGDYSVKLYDEEGYGAVRKAQRSGEENKVKPLATVVVRHPGTYT  120

PduK110    121   GPWFNSEILAAALIAFVAYFAFSTRSKILY  150
ParSP80    121   GPWFNSEILASGLIAVVAYFAFATRSKILS  150
ParSP80.2  121   GPWFNSEILASGLIAVVAYFAFATRSKILS  150
LJS138     121   GPWFNSEILAAGLIAVVAYFAFSTRSKILS  150
```
