# Supplementary material for: Comparative Evolution of Sand Fly Salivary Protein Families and Implications for Biomarkers of Vector Exposure and Salivary Vaccine Candidates
Source: Front Cell Infect Microbiol. 2018 Aug 29;8:290. doi: 10.3389/fcimb.2018.00290 (PMC6123390; doi:10.3389/fcimb.2018.00290)
Supplement: Supplementary Figure 12 — Multiple sequence alignment and molecular phylogenetic analysis of the sand fly Silk salivary protein family. (Top) Multiple sequence alignment of Silk. PPTSP32 (P. papatasi), PduK45 and PduM33 (P. duboscqi), PsSP44 (P. sergenti), PorASP86 (P. orientalis), ParSP02 (P. ariasi), PpeSP05 (P. perniciosus), PtSP29 (P. tobbi), PkanSP15 (P. kandelakki), PabSP30 (P. arabicus), PagSP06 (P. argentipes), Lolsilk (B. olmeca), LJL04 (Lu. longipalpis), LayS89 (Lu. ayacuchensis), Linb-26 (N. intermedia). Black background shading represents identical amino acids. Gray background shading represents similar amino acids. (Bottom) The evolutionary history of Silk salivary protein family was inferred by using the Maximum Likelihood method based on the JTT matrix-based model (Jones et al., 1992). Sand fly species are indicated by the different symbols in the legend on the right. Tree branches were color-coded so as to represent specific taxon: Green color represents the Larroussius and Adlerius subgenera; Red color indicates the Euphlebotomus subgenus; Blue color points to proteins of the Phlebotomus and Paraphlebotomus subgenera; and Black color indicates the proteins belonging to New World sand flies. [file Image_12.PDF]

```
PPTSP32    1  --STIPIQSQGQDFPVPFVSEQTD----DFYDDKFYPDISDDNINEVVRDN-GRK-----  48
PduK45     1  ----IPIKNQGKDFPVPFVSEQTD----DFYDDKFYPDIDDENINEVVRDNKGNRGAQSN  52
PduM33     1  --KPIPINNQGKHFPVPFVSQQNDG---DFYDDNYYPDINDESINTAVRDN-GGK-----  49
PsSP44     1  -AKTIPISKQGKNFPVPFVDPKETD---DFFDDQYYPDIDDESITEIVRDN---------  47
PorASP86   1  ALPAIPIAKQGKNFPVPFVSEENS--PDDYFDDQYYPDINDAGVSSKAPQGTRKPPNR--  56
ParSP02    1  APPAIPIAKQGNDFPVPIVDEKET---DDFFDDRFYPDIDDERVGARAPVGGKQTSNRGT  57
PpeSP05    1  ALPAIPIARQGKDFPVPFVSEDNN--PDDYFDDQYYPDINDAGVGSKAPQGSRKPPNR--  56
PtSP29     1  APPAIPIAKQGKDFPVPFVSEENN--PDDYFDDQYYPDINDGGVGSRAPQGTRKPSNR--  56
PkanSP15   1  ALPAIPIAKQGKDFPVPIVDEKIT---DDFFDDRFYPDIDDERVGPKAPEGTRKPPKK--  55
PabSP30    1  APQVIPIAKQGKDFPVPIVDEKTT---DDFFDDRYYPDIDDERVGPKAPTGTRQKPGS--  55
PagSP06    1  ARPAIPINKQGKDFPVPTADEKVT---DDYFDDRFYPDIDDEKIGVRKDNGQGSKGGS--  55
Lolsilk    1  -AKVIPIKKQGQNFPVPIVKPGS--S-DDYFDDQYYPDINDETIAEAPKDNKRSSVG---  53
LJL04      1  -ANEIPINRQGKDYPVPIIDPNKSSS-DDYFDDRFYPDIDDEGIAEAPKDNRGKSRGG--  56
LayS89     1  -ANEIPINQQGKNYPLPIADPDK--S-DDYFDDRFYPDINDESIVEAPKDNRGKPGGG--  54
Linb-26    1  -AKVIPIKQQGKNFPVPQANPAN--S-DDYFDDQFYPDINDEKIGEAPRDNRGKTGGG--  54

PPTSP32   49  ---GGDRGSQSTPSGK--------------------------ESHPSATQTGGRRPSQSP  79
PduK45    53  VPAGGSRPSATPTSGGRPSQP-----------------PSRGETRPSVTPSRDRRPSQSS  95
PduM33    50  ---GDSRGSQSKPSGK--------------------------ETRPSATQTGGRRQSNPS  80
PsSP44    48  ------QGYQSKPSGD--------------------------KSRPSATPNSGQRP--PG  73
PorASP86  57  -------ETIPPPRG---------------------------DQVS-----GGRTPP---  74
ParSP02   58  ---SSQSDKVPRPQGSNRGPSSQTTDKVPRPQWPSRGTNSQNDKVPRPQGSSGQTPPRTP  114
PpeSP05   57  -------GTIPPPRG---------------------------DQVSS----GGRTPP---  75
PtSP29    57  -------ETNP--RG---------------------------DQSKGP-VSGGRTLP---  76
PkanSP15  56  -------ETIPPPRG---------------------------DQIEGPQVPGGRTPPRAP  81
PabSP30   56  ------KGPQPTPGG-----------------------RTPPGSKGPQATPGGRTPPGSK  86
PagSP06   56  -------ASQSRPPAP--------------------------DKSP------GKTNVS--  74
Lolsilk   54  -----------PSGGKLG-------------------------KGNKRPGHGSGGTS--V  75
LJL04     57  -------GAAGAREGRLGTN------------------GAKPGQGGTRPGQGGTRPGQGG  91
LayS89    55  -------SKPAAAPG-----------------------GARLGAGGTTPGRGATTPGGGG  84
Linb-26   55  -------GTGASSGGRQGG--------------------ARPGKGGKRPGQGSRRPG--G  85

PPTSP32   80  CGESRPSGS------------------------ATSGRRPSQSPRGE-------------  102
PduK45    96  RREPCASGS------------------------PTRGRTPSQSPEGEPRPSATFPSSSDR  131
PduM33    81  KGESRPSAT------------------------PTGGRRPSKSPGGELPPRTTFPSSG-W  115
PsSP44    74  RAET-PPAS------------------------PAS----SASP----------------  88
PorASP86  75  ERVGQGAS------------------------TGTNNRRPGAQNNRNPTGS---------  101
ParSP02  115  GKVEQSGR------------------------TNTKDQIPRPLTNRNPTKNPT-------  143
PpeSP05   76  GRVGQGTSPTKDKRARPQINRNPTGTVGQGGSPGTKDKRARPQINRNPTGS---------  126
PtSP29    77  GSVG------------------------------TKDQKPGPQINRNPTGS---------  97
PkanSP15  82  GKVGQGAS------------------------TGTKNQTPGSLANRNPTGS---------  108
PabSP30   87  GPQATPGGRTPP-------------------GRGGQGGKPGGKDQRTGPATG--------  119
PagSP06   75  DKRADPPK------------------------ASPCDRKSGRKGVRD-------------  97
Lolsilk   76  TRPGQSG----------------------------SSARPASGGTS--------------  93
LJL04     92  TRPGQGGTR-----------------------PGQGGTRPGQGRTKPAQGTTRPAQGTRN  128
LayS89    85  TRPSAGGSR-----------------------QNTGRTRPAAGGTRRGQGGTRADQGRQR  121
Linb-26   86  TRPGQGG----------------------------IAT---QGDTS--------------  100

PPTSP32  103  ------------------------------SLPPATLAGR-------------QNSRQQD  119
PduK45   132  ESLPFPRGQFPIPDTFPTKGVESLPNSGGVSRPSVTLPGSDRTQWGGYETSRGQNSRQQG  191
PduM33   116  GSSQVPLEESQPSATFPTKSWDSLPLPGRGSRPSDTLPSSARRPCDGFDTSSRQNSRQPG  175
PsSP44    89  -------------------------------APPCRNPGQ--------------QGRKQG  103
PorASP86 102  ----------------------GQKPKDRELQIKDKTPSGSQGG---------KPGGQVR  130
ParSP02  144  --------------------EQARRPGNRELLIRDKTP-GSQGG---------K---QGT  170
PpeSP05  127  ----------------------GTKPRDRELVIRDKPPSGSQGG---------KPGRQVR  155
PtSP29    98  ----------------------GAKPKDRELVIRDKTPSGDQGG---------KPGRLGK  126
PkanSP15 109  ----------------------GTRSRDRELVIRDKTPSGGQAG---------KPERQGS  137
PabSP30  120  --------------------KWGKGSQGKELRIREKTT----------------PVRQGR  143
PagSP06   98  ------------------------RTKNRELVIDESTARVRQNS---------QDRKQNH  124
Lolsilk   94  ------------------------------------SVGSRNP----------NK--RGN  105
LJL04    129  PGSVGTKEAQDASKQGQGKRRPGQVGGKRPGQANAPNAGTRKQ----------QKGSRGV  178
LayS89   122  PG----------NNQG-GTRQGG--GASRPAQG---AAGGRKQG---------TKGTKGA  156
Linb-26  101  ------------------------------------SVGQRKP----------QKGGRGN  114

PPTSP32  120  RRQNKKQPDLSKYKNSPARYIFTTGNVD-SGKTPDEERIFRTNRAEYVLATGGPYDNYLV  178
PduK45   192  RRQDRKQPDLSKYKNSPAKYIFATGNVD-SGKEPDEVRMFRTKRPEYELATGDPYNNYLV  250
PduM33   176  RQQNRNQPSLSNYRNSPAKYIFTSGYVD-SSKKPDEERLFRTNKKEYTIATGDPYTNYLV  234
PsSP44   104  KKGQKKQ-DLSRYKNSPAKYIFRTGNID-PGKTPDDVRLFSTSQPEYVIASGNPYDDYVV  161
PorASP86 131  GS----KEDLSRYKNAPAKLIFKSSNINTTGKTPNAVKLFKTKKAKTVVAKGGPNDVYEV  186
ParSP02  171  GN----RQKLSSYKDAQPKLIFKSSQFNTDGQNPYLTRLFKTKKVEEVIAKGSPTDEYVL  226
PpeSP05  156  GP----KEDLSRYQNAPAKLIFKSSNINTAGKTPSAVKLFKTKKDKTVVAKGGPNDVYEV  211
PtSP29   127  GS----KEDLSRYKNAPAKLIFKSSNINTAGKTPNAVRLFKTKKAKTVIAKGGPNDVYVV  182
PkanSP15 138  GK----KQDLSRFKNAPAKLIFKSSNFDTTGKTPYAQRLFKTQKPQTVIAKGDPNDVYVL  193
PabSP30  144  GN----RQDLSSYKNAQPKLIFKSSQFSTNGKIPSAVKLFRTKKSEEVITTGSPTDEFVV  199
PagSP06  125  KQNRPVQRNLQSYKDAPATYVFKSYDFRENGRTP-IVKLFETNKAEEVIAKGGRNDEYVL  183
Lolsilk  106  GQ-----KLKSKYANTPAKYIFKSPKFNDAGKTP-IVTYFKTKNKQHVVARGSPNDEYVM  159
LJL04    179  GR-----PDLSRYKDAPAK-FFKSPDFSGEGKTP-TVNYFRTKKKEHIVTRGSPNDEFVL  231
LayS89   157  NRR----SDLSKYKDSPAKYIFKSPSFNEEGKTP-IVNYFKTSKKEYTAAGGGPNDEYVL  211
Linb-26  115  GQ-----GLKAKYANTPVKSIFKSPHFNDAGITP-TVKYFKTKNKEHIMARGGANDEFVL  168

PPTSP32  179  EIIDGPNPNDISLKQSTTMGGDSKLILDNPNRNTIVGRIKTFKA-------  222
PduK45   251  EIIEGPNPSDISLKQSTVMGGDSKIILENPTGRTIVGRIKTYKGEKKGN--  299
PduM33   235  EIIQGPDPNDIGLKQLTTMDGDSRLILENPTGETVVGRVKLTGRERKGN--  283
PsSP44   162  EIIEGP-TSDLKLKQATIMGRESRLILDNPSRGKVIGRVKTYKA-------  204
PorASP86 187  ELLDGN-FNNMSLRIQIMDRKSSTAILSNPDRNLIVGRVKTYR-GLR----  231
ParSP02  227  ELLDGK-PDNLSLVIRTNG-KTSQAVLRNPTRNRIVGRIKSYNPGPRRMSY  275
PpeSP05  212  ELLDEN-FNNMSLRIQIMDRKSSTAILSNPDRNLIVGRVKTYR-GLR----  256
PtSP29   183  ELLDGN-FNNMSLRIQIMDRKSSTAILSNPDRNSIVGRVKTYR-GLR----  227
PkanSP15 194  ELLDGD-YQNLSLGIKIMGRSKSIATLHNPNRNAIVGRIKTYK-GLR----  238
PabSP30  200  ELLDGR-LDNLSLRIETMG-QNSKVILRNPNRNRIVGRVKTYKNAYSG---  245
PagSP06  184  DILDGK-PYKLSLKMDATG----TVTVSNPDRERIVGRLKTYKA-------  222
Lolsilk  160  EILEGD-PSGIVLSIQTIG-NESQVIVKNPNGKPIVGRMKVYKNGYRG---  205
LJL04    232  EILDGD-PTGLGLKSETIG-KDTRLVLENPNGNSIVARVKIYKNGYSG---  277
LayS89   212  EIVDGD-PSGLGLAVQTIG-KDSRLILKNPKGNNIVGRVKIYRGAYTG---  257
Linb-26  169  EILEGD-TSGLRMSVETVG-SESRAVLKNPNEKSIVGRVKTYKDGYRRSG-  216
```

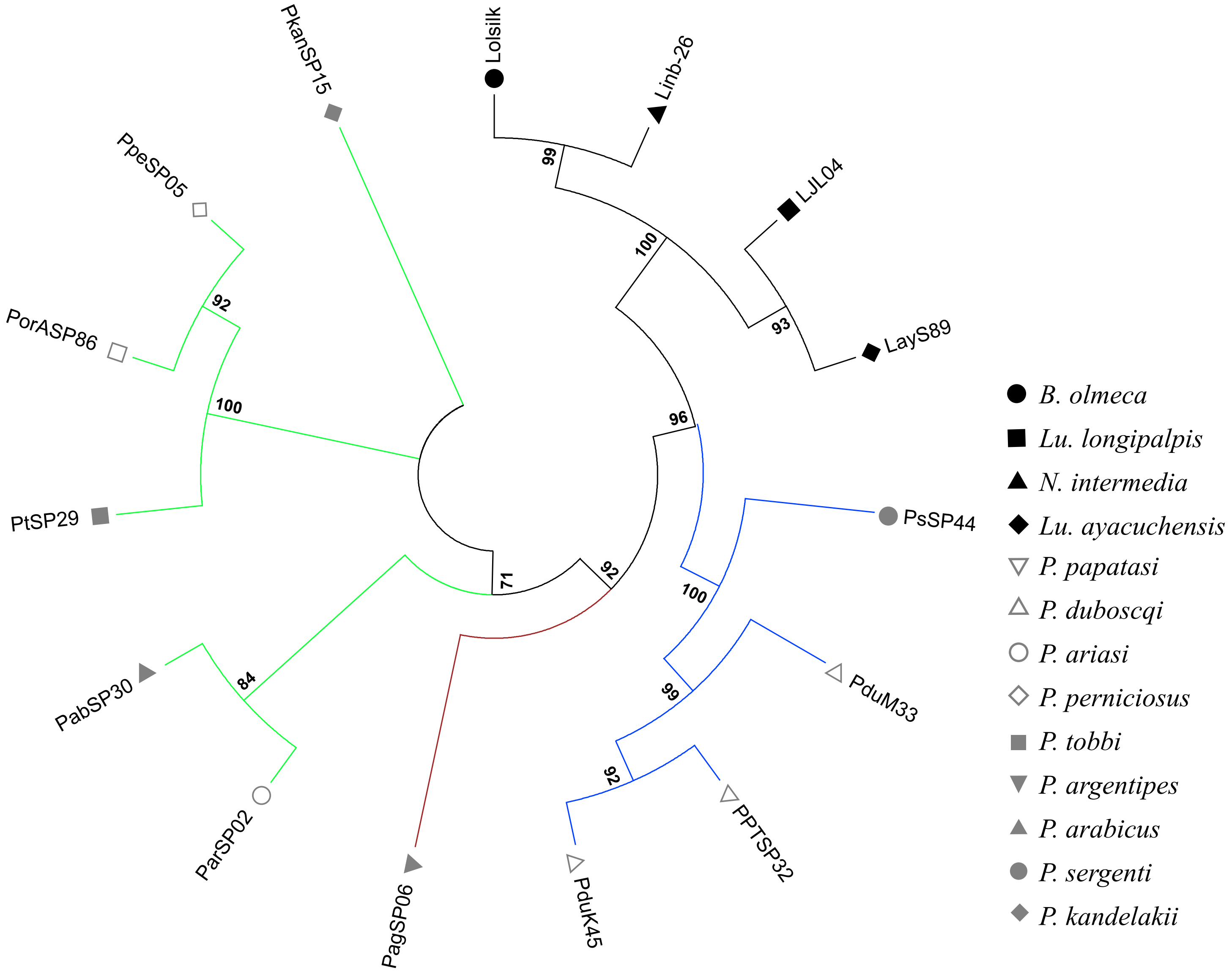

Lolsilk
Linb-26
LJL04
LayS89
PkanSP15
PpeSP05
PorASP86
PtSP29
PabSP30
ParSP02
PagSP06
PduK45
PPTSP32
PduM33
PsSP44
99
100
93
96
92
100
84
71
92
100
99
92
B. olmeca
Lu. longipalpis
N. intermedia
Lu. ayacuchensis
P. papatasi
P. duboscqi
P. ariasi
P. perniciosus
P. tobbi
P. argentipes
P. arabicus
P. sergenti
P. kandelakii
