# Supplementary material for: Comparative Evolution of Sand Fly Salivary Protein Families and Implications for Biomarkers of Vector Exposure and Salivary Vaccine Candidates
Source: Front Cell Infect Microbiol. 2018 Aug 29;8:290. doi: 10.3389/fcimb.2018.00290 (PMC6123390; doi:10.3389/fcimb.2018.00290)
Supplement: Supplementary Figure 13 — Multiple sequence alignment and molecular phylogenetic analysis of the sand fly Yellow salivary protein family. (Top) Multiple sequence alignment of Yellow. PPTSP42 and PPTSP44 (P. papatasi), PduK06 and PduM10 (P. duboscqi), PsSP22 and PsSP26 (P. sergenti), ParSP04 and ParSP04b (P. ariasi), PorASP2 and PorASP4 (P. orientalis), PtSP37 and PtSP38 (P. tobbi), PpeSP03B and PpeSP03 (P. perniciosus), PkanSP04 (P. kandelakki), PabSP26 (P. arabicus), PagSP04 (P. argentipes), LolYLWb and LolTLWc and LolYLWA (B. olmeca), Linb-21 (N. intermedia), LayS22 and LayS118 (Lu. ayacuchensis), and LJM17 and LJM17 and LJM111 (Lu. longipalpis). Black background shading represents identical amino acids. Gray background shading represents similar amino acids. Asterisks indicate the conserved cysteine residues. (Bottom) The evolutionary history of Yellow salivary protein family was inferred by using the Maximum Likelihood method based on the Whelan And Goldman model (Whelan and Goldman, 2001). Sand fly species are indicated by the different symbols in the legend on the right. Tree branches were color-coded so as to represent specific taxon: Green color represents the Larroussius and Adlerius subgenera; Red color indicates the Euphlebotomus subgenus; Blue color points to proteins of the Phlebotomus and Paraphlebotomus subgenera; and Black color indicates the proteins belonging to New World sand flies. Names in bold represent 44 kDa protein homologs. [file Image_13.PDF]

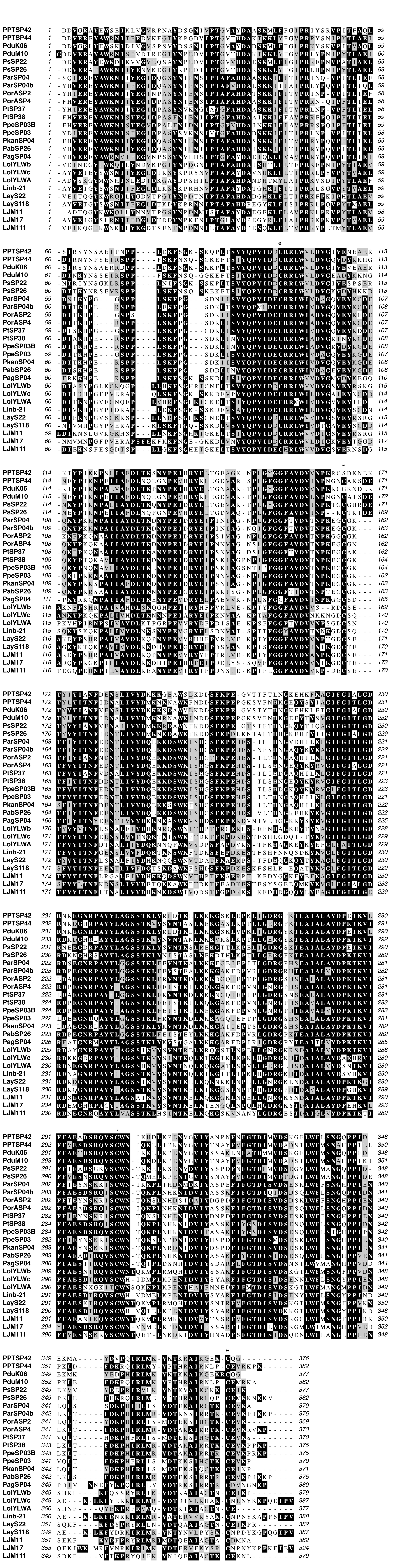

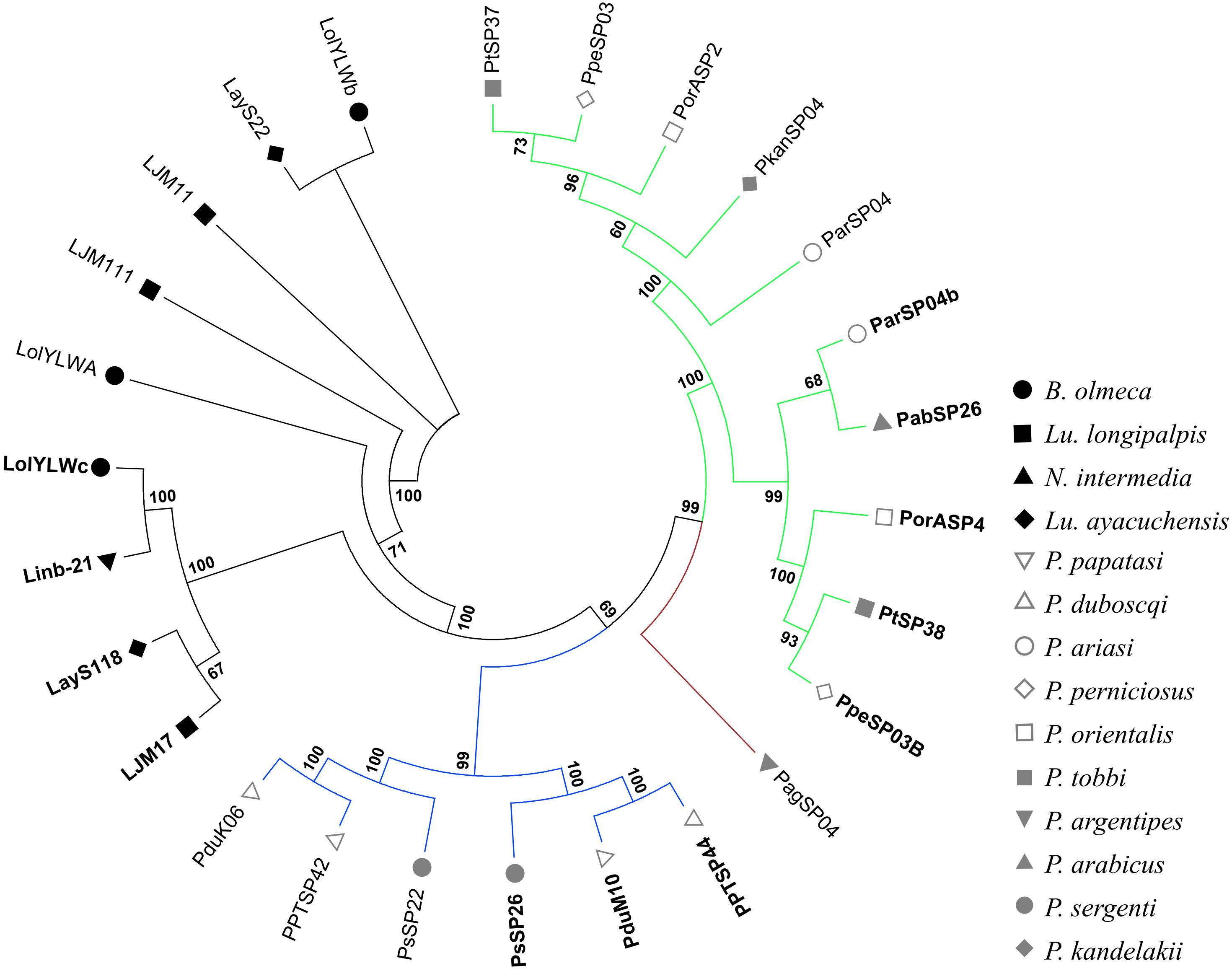

LolYLWb
LayS22
LJM11
LJM111
LolYLWA
LolYLWc
Linb-21
LayS118
LJM17
PduK06
PPTSP42
PsSP22
PsSP26
PduM10
PPTSP44
PagSP04
PpeSP03B
PtSP38
PorASP4
PabSP26
ParSP04b
ParSP04
PkanSP04
PorASP2
PpeSP03
PtSP37
B. olmeca
Lu. longipalpis
N. intermedia
Lu. ayacuchensis
P. papatasi
P. duboscqi
P. ariasi
P. perniciosus
P. orientalis
P. tobbi
P. argentipes
P. arabicus
P. sergenti
P. kandelakii
