# Supplementary material for: Comparative Evolution of Sand Fly Salivary Protein Families and Implications for Biomarkers of Vector Exposure and Salivary Vaccine Candidates
Source: Front Cell Infect Microbiol. 2018 Aug 29;8:290. doi: 10.3389/fcimb.2018.00290 (PMC6123390; doi:10.3389/fcimb.2018.00290)
Supplement: Supplementary Figure 14 — Multiple sequence alignment of the sand fly Adenosine deaminase salivary protein family. PduM73 (P. duboscqi), PpeAda (P. perniciosus), and LJLAda (L. longipalpis). Black background shading represents identical amino acids. Gray background shading represents similar amino acids. [file Image_14.PDF]

```
PduM73   1  VLDISNIKPKRDYENFLQKYAEYADDEVDRSVGSDITLSLKEKFVNQYLMDLKTEELKAG  60
PpeAda   1  -FDWTEFRMERKYKDYLQKRSYYLAEEEDRSVGSDIELTAKEQIVNERLMALKRKELAEG  59
LJLAda   1  -WDN-SWIMDMKYERYSQRRSYYLAEEEDRSVGSDIELTAKEQVVNERLMELKMTELKNG  58

PduM73  61  LKNPSQFIPSNHFFSVLDRINSSEIFKIIRRMPKGAILHAHDTALCSTDYVVSITYRDHL  120
PpeAda  60  LENPAGFIPWNHMFDVLNRINSSEIFDILRRMPKGGILHAHDTALCSTDYVISLTYEPNL  119
LJLAda  59  LQDPAGFIPWNHIFDVLYRINSSELFHIIQKMPKGGILHAHDTALCSTDYVISLTYEPNL  118

PduM73 121  WQCADPKTGALQFRFSKESPKNTDTCQWTPVSEERKNQGEEQYNSKLRSQLSLYNTDPIN  180
PpeAda 120  WQCTDPETGALSFKFSREAPTNTETCQWTSVAAERTKLGEENYNSGLRSQLSLYTTDPIN  179
LJLAda 119  WQCADPTTGAFQFLFSREAPTNTDTCTWTLVADERAKQGEENYNSALRSQLSMYNTNPIM  178

PduM73 181  RSRDVDSIWNDFMGLFGVNFGLLTYAPVWKDYYKQFLKEMMEDGVQYLELRGTLPPLYDL  240
PpeAda 180  HNRDVDSIWRQFMGIFGVNDGLLSYAPIWKAYYKQFLKEMMEDGVQYLELRGTLPPLYDL  239
LJLAda 179  HNRDVDSIWRQFMGIFGVNGGLLTYAPVWKAYYLQFLKEMFADGVQYLELRTTLPPLYDL  238

PduM73 241  DGKIYNEEQVVEIYYNVTEEFKKENSTFIGAKFIYAPVRFVNATGIKTLTTTVKQLHERF  300
PpeAda 240  DGKTYHEEEILHIYQDATREFKQENPTFIGAKFIYAPVRVVDDAGLPALMDKVRELHEQY  299
LJLAda 239  DGKTYNEVEIMQIYYDATKEFKKQNPTFIGAKIIYAPVRVVDDAGIPALMAKVRELHEKF  298

PduM73 301  PDFLAGFDLVGQEDKGGPLIGFSRELLELPESINFFFHSGETNWNGMTDDNLIAAVTLGT  360
PpeAda 300  PNFMAGFDLVGQEDKGRPLIDFSKEILGLPDSINFFFHAGETNWNGLTDDNLIDAVLLGT  359
LJLAda 299  PDFMAGFDLVGQEDKGRPLIAFSREILKLPNSIDFYFHAGETNWDGMTDDNLIDAVLLGT  358

PduM73 361  KRIGHGYALFKHPRVLKQVKKDKIAIEVCPISNQVLRLVADMRNHPGSILLANKKYPMVI  420
PpeAda 360  KRIGHGYAVLKHPRVLKQVKRDKIALEVCPVSNQVLRLVADMRNHPGAVLLANKEYPVVI  419
LJLAda 359  KRIGHGYAVLKHPRVLKEVKRNKIAIEVCPASNQVLRLVADYRNHPGSVLLANKEYPVVI  418

PduM73 421  SSDDPSFWEATPLSHDFYMAFMGLASYHQDLRMLKQLAINSLEYSSMTLEEKTNAMKLWE  480
PpeAda 420  SSDDPSFWEAKPLTHDFYMAFLGLASERQDLRLLKQLAINSIKYSAMTPLEKMNAMRLWE  479
LJLAda 419  SSDDPSFWEAKPLSHDFYMAFLGLASSRQDLRLLKQLAINSIKYSAMSPREKLQAMQMWE  478

PduM73 481  AEWEKFIKELETEVFSLLE  499
PpeAda 480  AEWKKFIEELSA-------  491
LJLAda 479  AEWKKFIDGFNA-------  490
```
