# Supplementary material for: Comparative Evolution of Sand Fly Salivary Protein Families and Implications for Biomarkers of Vector Exposure and Salivary Vaccine Candidates
Source: Front Cell Infect Microbiol. 2018 Aug 29;8:290. doi: 10.3389/fcimb.2018.00290 (PMC6123390; doi:10.3389/fcimb.2018.00290)
Supplement: Supplementary Figure 15 — Multiple sequence alignment and molecular phylogenetic analysis of the sand fly small Odorant Binding Protein (OBPs) salivary protein family. (Top) Multiple sequence alignment of OBPs. PPTSP12 and PPTSP14 and PPTSP14.2a and PPTSP15 (P. papatasi), PduM12 and PduM60 and PduM07 and PduM50 and PduM57 and PduM31 and PduM49 and PduM58 and PduM62 and PduM99 and PduM02 and PduM03 and PduM06 (P. duboscqi), PsSP14 and PsSP14 and PsSP55 and PsSP9 (P. sergenti), PtSP9 and PtSP17 and PtSP32 and PtSP31 and PtSP18 and PtSP23 (P. tobbi), PpeSP02 and PpeSP09 and PpeSP11 (P. perniciosus), PorASP28 and PorASP31 and PorASP37 and PorASP61 and PorASP61 and PorASP64 (P. orientalis), ParSP03 and ParSP01 and ParSP08 (P. ariasi), PkanSP05 and PkanSP06 and PkanSP07 (P. kandelakki), PabSP45 and PabSP2 (P. ariasi), PagSP93 and PagSP07 and PagSP01 and PagSP12 and PagSP02 and PagSP13 (P. argentipes), LolSOBPa and LolSOBPb and LolSOBPc (B. olmeca), Linb-7 and Linb-8 and Linb-28 (N. intermedia), LayS69 (Lu. ayacuchensis), and LuloOBP (Lu. longipalpis). Black background shading represents identical amino acids. Gray background shading represents similar amino acids. Asterisks indicate the conserved cysteine residues. (Bottom) The evolutionary history of OBPs salivary protein family was inferred by using the Maximum Likelihood method based on the Le_Gascuel_2008 model (Gomes et al., 2008). Sand fly species are indicated by the different symbols in the legend on the right. Tree branches were color-coded so as to represent specific taxon: Green color represents the Larroussius and Adlerius subgenera; Red color indicates the Euphlebotomus subgenus; Blue color points to proteins of the Phlebotomus and Paraphlebotomus subgenera; and Black color indicates the proteins belonging to New World sand flies. [file Image_15.PDF]

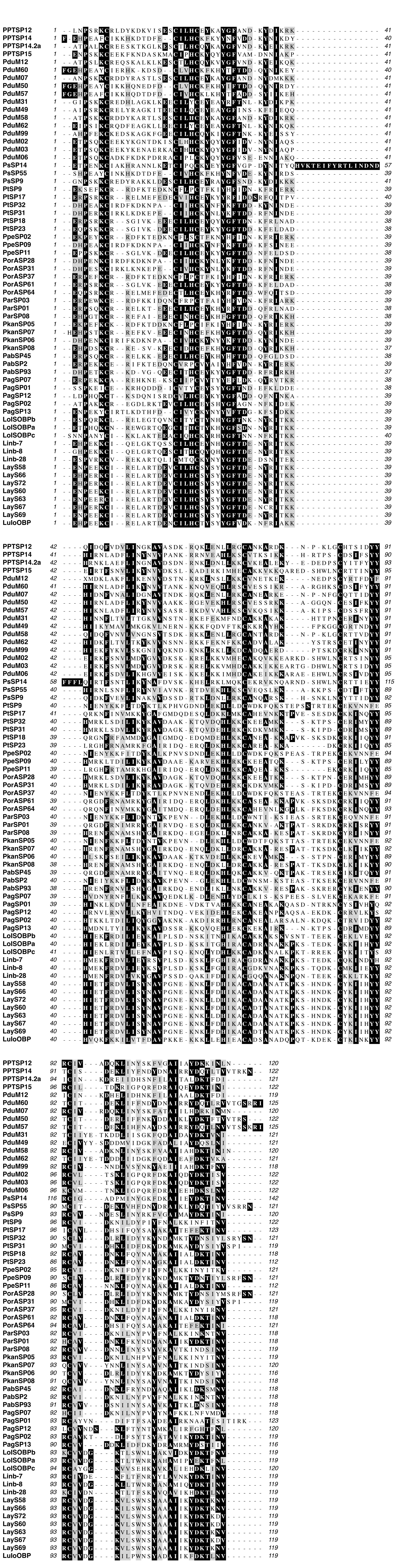
PPTSP12
PPTSP14
PPTSP14.2a
PPTSP15
PduM12
PduM60
PduM07
PduM50
PduM57
PduM31
PduM49
PduM58
PduM62
PduM99
PduM02
PduM03
PduM06
PsSP14
PsSP55
PsSP9
PtSP9
PtSP17
PtSP32
PtSP31
PtSP18
PtSP23
PpeSP02
PpeSP09
PpeSP11
PorASP28
PorASP31
PorASP37
PorASP61
PorASP64
ParSP03
ParSP01
ParSP08
PkanSP05
PkanSP07
PkanSP06
PkanSP08
PabSP45
PabSP2
PabSP93
PagSP07
PagSP01
PagSP12
PagSP02
PagSP13
LolSOBPb
LolSOBPa
LolSOBPc
Linb-7
Linb-8
Linb-28
LayS58
LayS66
LayS72
LayS60
LayS63
LayS67
LayS69
LuloOBP

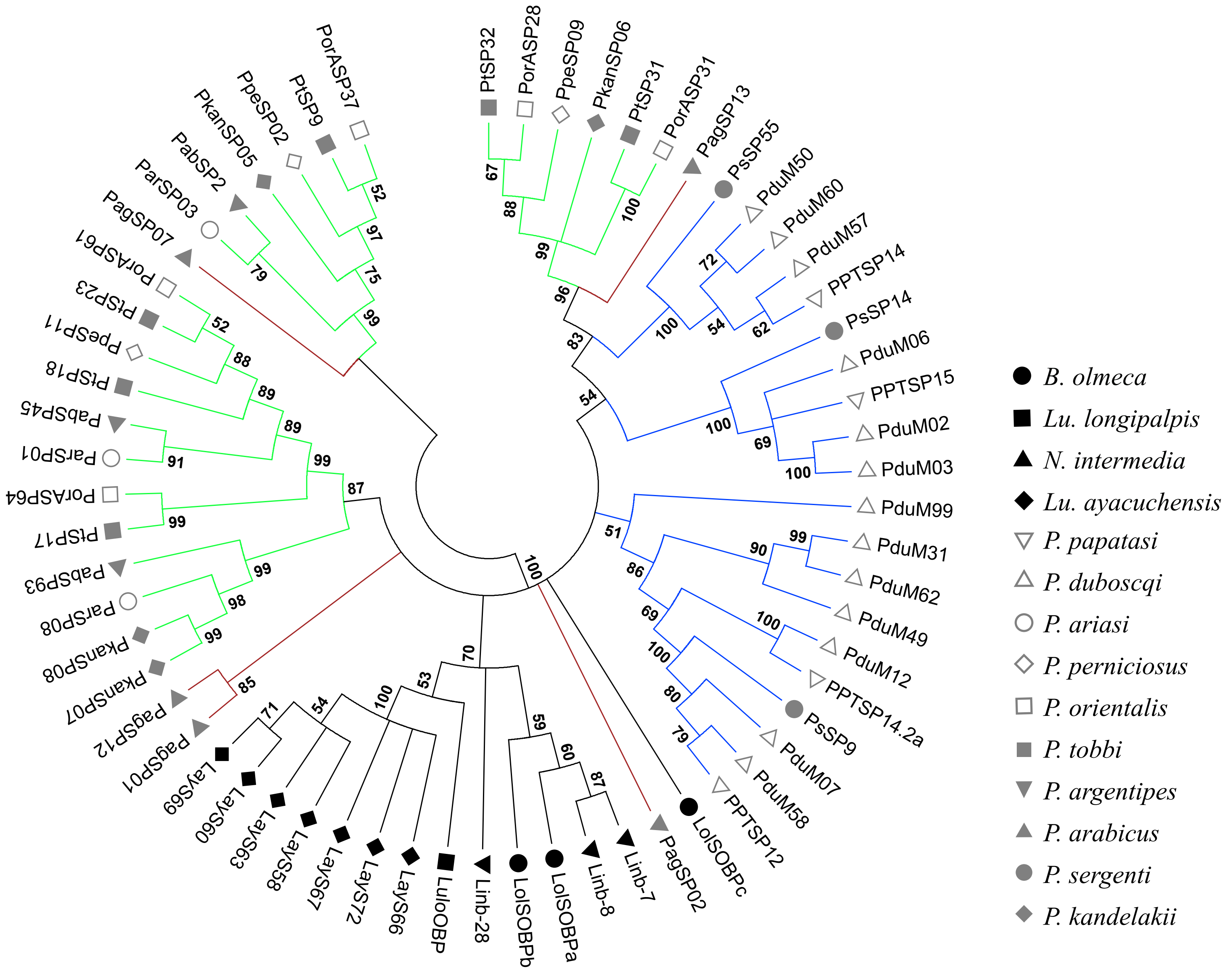
PtSP32
PorASP28
PpeSP09
PkanSP06
PtSP31
PorASP31
PagSP13
PsSP55
PduM50
PduM60
PduM57
PPTSP14
PsSP14
PduM06
PPTSP15
PduM02
PduM03
PduM99
PduM31
PduM62
PduM49
PduM12
PPTSP14.2a
PsSP9
PduM07
PduM58
PPTSP12
LolSOBPc
PagSP02
Linb-7
Linb-8
LolSOBPa
LolSOBPb
Linb-28
LuloOBP
LayS66
LayS72
LayS67
LayS58
LayS63
LayS60
LayS69
PagSP01
PagSP12
PkansP07
PkansP08
ParSP08
PabSP93
PtSP17
PorASP64
ParSP01
PabSP45
PtSP18
PpeSP11
PtSP23
PorASP61
PagSP07
ParSP03
PabSP2
PkanSP05
PpeSP02
PtSP9
PorASP37
B. olmeca
Lu. longipalpis
N. intermedia
Lu. ayacuchensis
P. papatasi
P. duboscqi
P. ariasi
P. perniciosus
P. orientalis
P. tobbi
P. argentipes
P. arabicus
P. sergenti
P. kandelakii
